# A multi-focus image fusion method via region mosaicking on Laplacian pyramids

**DOI:** 10.1371/journal.pone.0191085

**Published:** 2018-05-17

**Authors:** Liang Kou, Liguo Zhang, Kejia Zhang, Jianguo Sun, Qilong Han, Zilong Jin

**Affiliations:** 1 College of Computer Science and Technology, Harbin Engineering University, Harbin 150001, China; 2 School of Computer and Software, Nanjing University of Information Science & Technology, Nanjing 210044, China; Mar Ephraem College of Engineering & Technology, INDIA

## Abstract

In this paper, a method named Region Mosaicking on Laplacian Pyramids (RMLP) is proposed to fuse multi-focus images that is captured by microscope. First, the Sum-Modified-Laplacian is applied to measure the focus of multi-focus images. Then the density-based region growing algorithm is utilized to segment the focused region mask of each image. Finally, the mask is decomposed into a mask pyramid to supervise region mosaicking on a Laplacian pyramid. The region level pyramid keeps more original information than the pixel level. The experiment results show that RMLP has best performance in quantitative comparison with other methods. In addition, RMLP is insensitive to noise and can reduces the color distortion of the fused images on two datasets.

## Introduction

Because of limited depth of field, high magnification optical cameras, such as microscopes or macro-photography, cannot capture an object that is totally in focus. When capturing the object/scene with the camera, typically only one or a few small regions of the image lying in focus are clear. Multi-focus images fusion is a common remedy to solve this problem. A set of images with different focuses are captured, then fused to produce an ‘all-in-focus’ image that is clear everywhere [1] [2] [3] [4] [5] [6]. The process of synthesizing the all-in-focus image is called multi-focus image fusion. Multi-focus images fusion has been proven valuable in many applications such as microscope imaging [7], image deblurring [8], shape from focus [7] [9] and information forensics [10].

According to fusion methods, the multi-focus images fusion can be categorized into two types: transform domain based and spatial domain based methods. In the former one, all the source images are firstly transformed from spatial domain to transform domain by using Fourier transform, multi-scale decomposition or other methods. After being combined by certain guidelines, the fused coefficients are inversely transformed back to spatial domain to obtain the expected fusion image. Many transform domain based algorithms are brought forward, such as the Laplacian pyramid (LP) [11], the Discrete Wavelet Transform (DWT) [12] [13], the gradient pyramid [14] [15], the contrast pyramid (CP) [16], the ratio-of-low pass pyramid [17], the Shift-invariant DWT (SIDWT) [18], the Complex Wavelet Transform (CWT) [19] [20] and the Contourlet Transform [21] [22] [23].

Unlike transform domain based ones, the spatial domain based methods refer to the strategies that the source images are fused directly in gray space. These algorithms include weighted average fusion, weighted linear fusion, Principal Component Analysis (PCA) [24], Neutral Network, etc. The spatial domain based multi-focus image fusion can also be divided into two categories: pixel based and block based methods. In the pixel based methods, pixels of source images are fused directly after exactly matching source images. The pixel based fusion methods can significantly improve the visual effects of the fused image, however, it is sensitive to noise and misregistration [25] [26]. When applying the block based methods, source images are firstly divided into *N* × *M* blocks. After comparing the sharpness of the blocks at corresponding position, the blocks with higher sharpness are spliced together to produce an all-in-focus image [16]. This kind of methods may result in block artifacts. In addition, the size of blocks can significantly affect the image fusion quality. Compared with the spatial domain based ones, the transform domain based methods can produce better fusion effect, whose limitations are the high cost and complexity of its computation.

As a representative of transform domain methods, the pyramid based methods have already been researched widely. Since these methods belong to the pixel level image fusion in a broad sense, most of them are sensitive to noise. Noise pixels and sharp regions of image are both high frequency signal, so it is hard to distinguish them by their frequency bands. The noises can be falsely identified as the pixels on the focal plane. The transform domain based methods apply global information to produce all-in-focus image, therefore a small change (is caused by noise) of any coefficient in the transformed domain may lead to changes of all pixels in spatial domain [27]. To solve the problem of noisy sensitivity, gradient map filtering(a directional change in the intensity or color in an image) [14] and multiple coefficient selection principles [28] are proposed, however, their performance depends on fine-tuned parameters.

For image fusion, the weighted approaches are more intuitive than pyramid-based methods [29] [30] [31]. When using weighted approaches, each pixel is assigned a weight which is calculated based on the focus degree of the source image. Fused image is the weighted summation of all corresponding pixels. Region mosaicking is a special weighted linear fusion, which sets one to the weight of the pixels lying at the focusing region, zero to the weight of the other pixels. In [32] Agarwala *et* *al*. proposed an ‘iterative digital photomontage’ method, which uses an interactive framework to combine source images into a single image. In the process, the graph cut algorithm is employed to segment the optimized mask. Mosaic algorithms often introduce block artifacts, while it can preserve original information of source images. In recent years, the sparse reconstruction methods are utilized to fuse weighted multi-focus images [10] [27] [29]. Sparse coefficients with the over complete dictionary are used to represent the multi-focus images. The coefficients are then combined with a choose-max rule, from which the fused image is reconstructed with respect to the over-complete dictionary. In the experiment results, the sparse weighted fusion achieved the highest performance among the existing weighted linear approaches. Recently, an artificial neural network model, pulse coupled neural network (PCNN), developed by Eckhorn *et* *al*. [33] has been employed in many applications of image fusion including weighted linear fusion [1] [34] [35] [36]. PCNN can automatically measure focus on the source images and then adjust weights of pixels. This method exhibits good performances in both visual effect and objective evaluation criteria, but a large number of its parameters often results in computational inefficiency.

**Fig 1:**
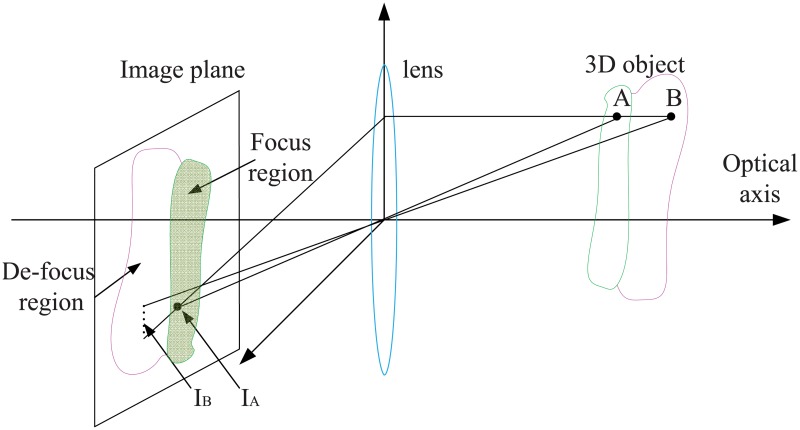
An illustration of 3D object imaging with an optical camera. *I*_*A*_ and *I*_*B*_ are image pixels of point *A* and *B* respectively. With the current focal length, the surface that point *A* lies on is focused while that of point *B* is not. It can be seen1 that in-focus pixels in the image plane form a continuous region. By adjusting the object distance to the lens, a series of defocused (part-in-focus) images could be obtained.

When observing micro object by microscope, the characteristic of the captured multi-focus images is that the focus regions of adjacent frames are continuous, as shown in Fig 1. The traditional fusion methods devote attention to discrete pixels on focus plane and do not fully utilize the continuity. For instance, when using pyramid method (such as Laplacian pyramid, contrast pyramid and gradient pyramid) to fuse a set of multi-focus images, a pixel and its neighbors often belong to different layers of pyramid. In this case, corresponding focally regions of different layers are not similar multiscale shapes, which suggest that a great many pixels of clear regions in source images are lost. In the final fused image fusion, errors and distortion may be produced because of the above problems. In [37], Hariharan *et* *al*. use adaptively segmentation of focally connected regions to synthesize an all-in-focus image. Their method employs overlapping focal regions to extend the depth of field, while retaining the visual verisimilitude of the scene.

This paper proposes a simple and effective approach, Region Mosaicking on Laplacian Pyramids (hereafter referred to as RMLP for short), to fuse multi-focus images. It is based on the observation that the in-focus pixels in a multi-focus image form continuous regions. RMLP uses Density-Based Region Growing (DBRG) to generate a focus region mask for all of the multi-focus images. In DBRG, both regions growing and regions filtering are used to identify appropriate focus regions and reduce the impact of noises. A segmented focused region mask is decomposed into a mask pyramid, which is then applied to supervise the region mosaicking on a Laplacian pyramid. RMLP also improves pixel level pyramid fusion at the region level, where the imaging characteristics of multi-focus images are utilized and the continuity of segmented focused regions is incorporated. In RMLP, decomposition values of a pixel at corresponding position of different pyramid layers are taken from the exactly same multi-focus image, which guarantees that distortion artifacts are reduced to minimum. In addition, RMLP can also significantly reduce the artifacts introduced by weighted linear fusion approaches.

The remainder of this paper is organized as follows. An overview of the proposed approach is introduced in Section 2. Then the focus region segmentation is described in Section 3. The image fusion procedure of RMLP is given in Section 4. After providing experimental results, the paper is concluded in Section 6.

## Overview of RMLP approach

**Fig 2:**
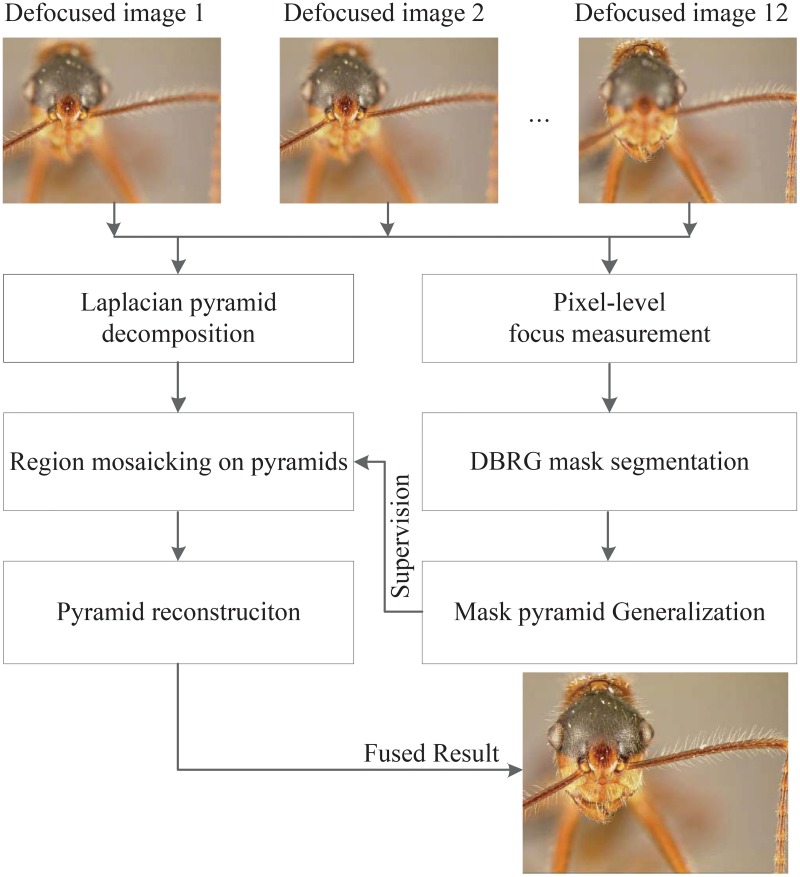
Flowchart of multi-focus images fusion based on RMLP approach.

The flowchart of RMLP is shown in Fig 2: the competition on Sum-Modified-Laplacian (SML) is used firstly to detect in-focus (clear) pixels in each image. The SML measurement is employed as the dominant cue for fusion, which guarantees that the information from multi-focus images is preserved. Fig 2 shows only three defocused images (the total number is 12) as the limitation of space.

DBRG is then applied to refine the extracted clear pixels, connect them into clear regions and form a focus region mask (Section 3). The focus region mask is then decomposed into a mask pyramid, corresponding to the Laplacian pyramids of multi-focus images. The mask pyramid contains label values of multi-layers, indicating which pyramid will be selected in the fusion procedure (Section 4).

Since the mask image contains continuous regions instead of disconnected pixels, in the fusion procedure, one segmented region will be is selected from each Laplacian pyramid image so that all the selected regions cover the pyramid. This procedure is referred as region mosaicking on pyramids. After region mosaicking, a reconstruction procedure is carried out to obtain the final ‘all-in-focus’ image as the output.

## Focus region segmentation

### Focus measurement based on Sum-Modified-Laplacian

For multi-focus image fusion, focus measurement needs to be done before focus region segmentation and image fusion. Therefore choosing an appropriate measurement method is crucial for subsequent process. In [38], Huang *et* *al*. contrastively analyzed several typical measurement methods, including Energy of image Gradient (EOG), Energy of Laplacian (EOL) of the image, Sum-Modified-Laplacian (SML), and spatial frequency (SF). In their experimental results, SML has the best performance in the aspect of image quality. Performance of EOL slightly worse than SML, but it is more computational efficient. For higher quality of fused image, SML is used to measure focus by RMLP. SML is presented by Nayar *et* *al*. in 1994 [39]. They firstly defined a modified Laplacian as

$$\begin{array}{c} \nabla_{M}^{2}I\left(x,y\right)=|\frac{\partial^{2}I}{\partial x^{2}}|+|\frac{\partial^{2}I}{\partial y^{2}}|. \end{array}$$ (1)

Where *I*(*x*, *y*) is a source image. In order to accommodate for possible variations in the size of texture elements, a variable spacing (*step*) between the pixels is used to compute the derivatives. In this paper, the difference between adjacent pixels is used to replace differential coefficient, i.e., *step* equals to one. The discrete approximation of Eq 1 is calculated as

$$\begin{array}{c} \begin{array}{c} \nabla_{M}^{2}I\left(x,y\right)=|2I(x,y)-I(x-1,y)-i(x+1,y)|\\ +|2I(x,y)-I(x,y-1)-i(x,y+1)| \end{array}. \end{array}$$ (2)

A small window of size (−*w*, *w*) is defined around a pixel (*i*, *j*), then compute the focus at the pixel (*i*, *j*) as the sum of modified Laplacian:

$$\begin{array}{c} SML=\sum_{x=i-w}^{i+w}\sum_{y=j-w}^{j+w}\nabla_{M}^{2}I\left(x,y\right),for\nabla_{M}^{2}I\left(x,y\right)\geq T. \end{array}$$ (3)

Where *T* is a discrimination threshold. In this paper, the size of windows is empirically set 3 × 3, i.e., *w* = 1.

### Focus region segmentation based on region growing

Focus regions have high sharpness, whose SML is much larger than the one in defocus regions. A mask image *M*_0_ is defined whose pixel values vary in [1, *N*]. The pixel-level mask image *M*_0_ is initially labeled with

$$\begin{array}{c} M_{0}\left(i,j\right)=\underset{n}{\arg\max}|SML_{n}\left(i,j\right)|,n\in \left[1,N\right]. \end{array}$$ (4)

Where *SML*_*n*_(*i*, *j*) is the SML of the *n*^*th*^ multi-focus image at pixel (*i*, *j*).

**Fig 3:**
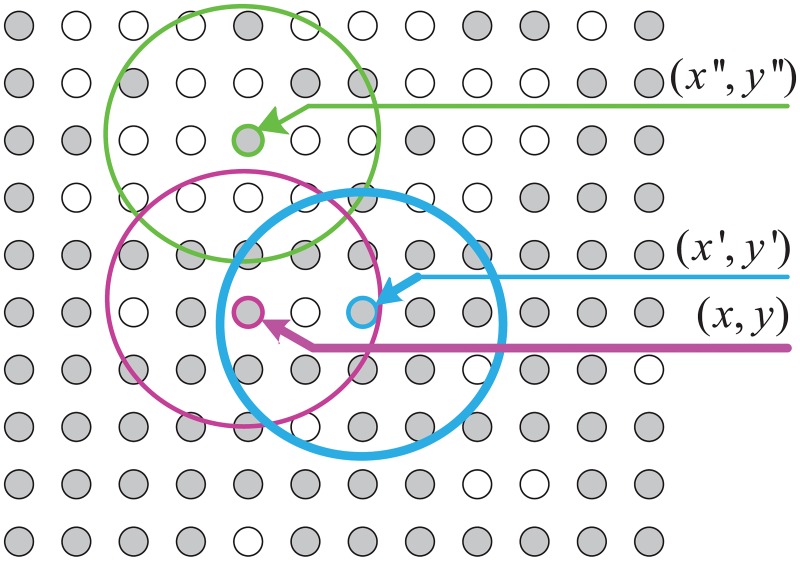
An illustration of density-connectivity with same mask label.

Image segmentation is applied in many scenarios as a common pretreatment method [40] [41]. The idea of Density-Based Region Growing (DBRG) image segmentation is that the pixels with same label are represented as a cluster. The density distribution of clusters is then analyzed. The spatial neighborhood Ω(*x*, *y*) of a given pixel (*i*, *j*) is defined as a circle centered at the pixel with radius *R*, where *R* is determined experimentally as shown in Fig 3 The density distribution in Ω(*x*, *y*) is defined as

$$\begin{array}{c} D_{\Omega (x,y)}\left(n\right)=(\frac{1}{\pi R^{2}}\sum_{\left(i,j)\in \Omega (x,y\right)}\delta (M_{0}(i,j)=n)). \end{array}$$ (5)

Where the Boolean function *δ*(⋅) is defined as

$$\begin{array}{c} \delta \left(x\right)=\{\begin{array}{c} 1,\;x\;\text{is}\;\text{true}\;\\ 0,\;\text{otherwise}\; \end{array} \end{array}$$ (6)

If the maximum density $\max_{n}(D_{\Omega (x,y)}\left(n\right))$ of Ω(*x*, *y*) is larger than a threshold 0.5, then the pixel (*i*, *j*) is called a seed pixel and Ω(*x*, *y*) forms a seed region, as shown by the gray pixels (circles) in Fig 3. In Fig 3 the pixel (*x*′, *y*′) is density-connected with pixel (*x*, *y*) if (*x*′, *y*′) is within the spatial neighborhood (mask) of (*x*, *y*). Since the pixel (*x*′′, *y*′′) does not belong to the spatial neighborhood, it is not the density-connected with (*x*, *y*) according to the density-connectivity defined above.

As a preprocessing of multi-focus image fusion algorithm, DBRG is to find clear focus regions with high sharpness, so segmented region must be close to the seed point and the smaller the segmented region the better. Therefore we employ simple “checker-board” distance as the distance metric. The “checker-board” distance is the most widely used metric in image segmentation. Euclidean distance is often used to express similarity measure between feature vectors of image. The topological distance can be used to segment maximum connected region of image [42]. However, considering too much noise will be introduced into the focus regions. DBRG segmentation algorithm takes a set of mask pixels as input and output a set of mask regions. The algorithm is presented in Algorithm 1.

**Algorithm 1**: Mask Segmentation

 **Input**: Pixel-level mask image *M*_0_

 **Output**: Region-level mask image *R*

**1** Set all of the pixels of *M*_0_ to unlabeled;

**2** Search the unlabeled pixels in *M*_0_ to find seed pixels and seed regions;

**3 for** *each seed pixel* (*x*, *y*) *and seed region* Ω(*x*, *y*) **do**

**4**  Create a new cluster with cluster label $R(x,y)=\underset{n}{\arg\max}(D_{\Omega (x,y)}\left(n\right))$

**5**  **for** *each unlabeled pixel* (*x*′, *y*′) *density-connected with* (*x*, *y*) **do**

**6**   Add (*x*′, *y*′) to the same cluster and mark (*x*′, *y*′) with label *R*(*x*, *y*);

**7**  **end**

**8 end**

**9 for** *each unlabeled pixel* (*x*″, *y*″) **do**

**10**  //noise pixels or pixels from smooth region.

**11**  Choose the labeled pixel most adjacent to (*x*″, *y*″); //let the pixel be (*x*, *y*).

**12**  Add (*x*″, *y*″) to the cluster of (*x*, *y*) and mark it with the corresponding label;

**13 end**

**14** Create a region-level mask image *R* based on clusters;

The density-connectivity among pixels is transitive due to density reachability, which is consistent with the imaging characteristics of multi-focus images. In defocus regions, focus measurements are not stable since the SML is small. Noisy pixels can have larger SML than the true focused pixels. The density based thresholding and growing can reduce the label errors on smooth regions or noisy pixels.

## Image fusion

After segmenting the focus region, the mask is generated with focused regions of source images. The mask is used to build the mask pyramid and supervise the fusion process of multi-focus images. This procedure is called region mosaicking on pyramid.

### Pyramid based fusion

The initial layer of Gaussian pyramid, *G*_0_, is the source image *I*(*x*, *y*). In [16], the *k*^*th*^ layer of Gaussian pyramid can be expressed as:

$$\begin{array}{c} \begin{array}{c} G_{k}(i,j)=\sum_{m=-2}^{2}\sum_{n=-2}^{2}w(m,n)G_{k-1}(2i+m,2j+n),.\\ k\in \left[1,K\right),i\in \left[0,R_{k}\right),j\in \left[0,C_{k}\right) \end{array} \end{array}$$ (7)

Where *w*(*m*, *n*) is a window function with Gaussian low-pass filter, *K* is the number of layers, *R*_*k*_ and *C*_*k*_ are the number of columns and rows in the *k*^*th*^ layer pyramid respectively. The bottom pyramid *G*_0_ constitute the Gaussian pyramid with other pyramids *G*_1_, …, *G*_*K*-1_.

Laplacian pyramid can be obtained through calculating difference between two adjacent layers of Gaussian pyramid. It is defined as

$$\{\begin{array}{l} LP_{k}=G_{k}-G_{k+1}^{*},k\in \left[0,K\right)\\ LP_{k}=G_{k},k=K \end{array}$$ (8)

$G_{k+1}^{*}$ is expanded *G*_*k*+ 1_, whose size is same with *G*_*k*_.

The classical Laplacian pyramid (LP) algorithm exploits a pixel level competition and fusion on pyramids as

$$\{\begin{array}{l} F_{k}\left(x,y\right)=LP_{k}^{\hat{n}}\left(x,y\right),\;k\in \left[0,K\right)\\ \hat{n}=\text{arg}\underset{n}{\;\text{max}}\left|LP_{k}^{n}\left(x,y\right)\right|,n\in \left[1,N\right] \end{array}$$ (9)

Where *F*_*k*_ denotes the *k*^*th*^ layer of the fused LP, $LP_{k}^{n}(x,y)$ denotes LP value of pixel (*x*, *y*) at *k*^*th*^ layer of the *n*^*th*^ multi-focus image and *N* denotes the total number of multi-focus images.

### Region mosaicking on Laplacian pyramid

According to Eq 9, the pixel-level fusion pyramid values are selected according to the difference-of-Gaussian magnitudes of their source images. It is found that regions selected for fusion in different pyramid layers are not similar figures, and the pixels which need to be reconstructed often come from more than one multi-focus images. As a result, the original clear information is lost and distortion is introduced. Therefore, the region based Laplacian pyramid fusion scheme, i.e., RMLP is proposed as following.

**Fig 4:**
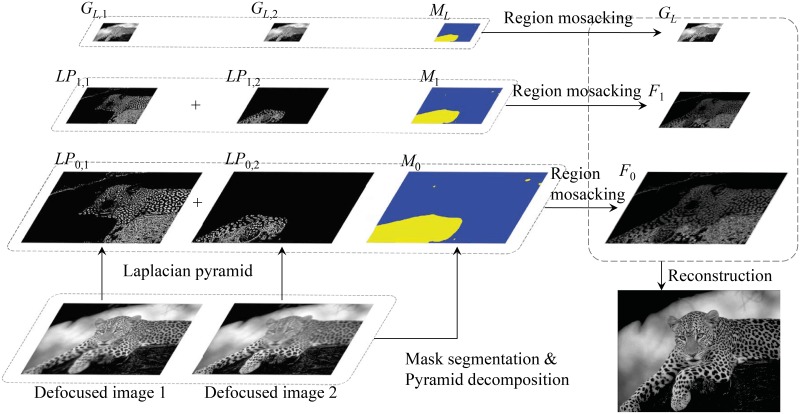
Illustration of RMLP with two multi-focus images and three pyramid layers.

In Fig 4, the focus label mask is also decomposed into the pyramid of three layers, *M*_*l*_, *l* = 0, 1, 2. In *M*_*l*_ blue and yellow regions indicate respectively focused regions of the defocused image 1 and 2. *LP*_*l*, 1_ and *LP*_*l*, 2_, (*l* = 0, 1) are the Laplacian pyramid of two defocused images, *G*_*L*, 1_ and *G*_*L*, 2_ are the base images of two Gaussian pyramids. *F*_0_ and *F*_1_ denote the fused Laplacian pyramids. The operator ‘+’ denotes the fusion operation of RMLP.

In RMLP, the segmentation mask *M*(*i*, *j*) is firstly decomposed into a mask pyramid, *M*_*k*_(*i*, *j*), *k* ∈ [0, *K*), which is then used to supervise a region level fusion of LP. The corresponding formulation is as follows,

$$\begin{array}{c} F_{k}\left(i,j\right)=LP_{k,M_{k}\left(i,j\right)}\left(i,j\right),\;k\in \left[0,K\right). \end{array}$$ (10)

Where *F*_*k*_(*i*, *j*) denotes the fusion result of pixel (*i*, *j*) at the *k*^*th*^ layer, *M*_*k*_(*i*, *j*) denotes the mask label of the *k*^*th*^ layer LP, i.e., which multi-focus image should be selected for fusion at pixel (*i*, *j*).

According to Eqs 8–10 the image fusion of the top pyramid is performed as

$$\begin{array}{c} F_{K-1}\left(i,j\right)=G_{K-1,M_{K-1}\left(i,j\right)}\left(i,j\right). \end{array}$$ (11)

Where *K* − 1=*L*=2 is the number of layers of *LP*, *F*_*K*−1_(*i*, *j*) denotes the fusion result of pixel (*i*, *j*) in the top layer image (*K*^*th*^ layer). *G*_*K*−1, *M*_*K*−1_(*i*, *j*)_(*i*, *j*) denotes the value of (*i*, *j*) of the base image. $M_{K-1}\left(i,j\right)=\underset{n}{\arg\max}|SML_{n}^{K-1}\left(i,j\right)|,n\in \left[1,N\right]$ denotes the label of pixel (*i*, *j*) of *K*^*th*^ layer mask image, calculated by the DBRG segmentation algorithm in Section 3.

In the region based fusion procedure, *M*_*k*+ 1_ is the down-sampling copy of *M*_*k*_, so corresponding regions in *M*_*k*_ and *M*_*k*+ 1_ are same figures. Consequently, most of the pixels (except for the pixels that lie on region transitive zones) in the fused image are reconstructed with pixel values from the same multi-focus images. As a result, much of the original clear information is kept and distortion is reduced.

When reconstructing the boundary areas, information from more than one multi-focus image is used. This may induce slight focus information loss in the transitive zones around the boundary areas. However, the usage of more than one multi-focus image information guarantees the gradual transformation of a transitive zone so that the block artifacts of the fused images is eliminated.

With the fusion results in the fused LPs(*F*_*k*_, *k* ∈ [0, *K*)), the low-pass-filtered each layer of Gaussian pyramid (*G*_*k*_), and the fused top layer image of LP (*F*_*K*−1_(*i*, *j*)), a reconstruction procedure is then carried out with

$$\begin{array}{c} \left\{ \begin{array}{c} G_{K-1}(i,j)=LP_{K-1}\\ G_{k}(i,j)=LP_{k}+G_{k+1}^{*},k\in \left[0,K-1\right) \end{array}.\right. \end{array}$$ (12)

Where *G*_*k*_ and G* are derived from Eqs 7 and 8. The reconstruction starts from the bottom layer Gaussian pyramid, *G*_0_; then it iteratively calculates the *G*_*K*−1_. According to the Eq 12, a Gaussian pyramid can be iteratively calculated from the top layer of the Laplacian pyramid. Finally, the bottom image of the Gaussian pyramid, *G*_0_, is precise reconstructed all-in-focus image.

## Experiment results

A great number of the fusion approaches are carried in grayscale image. For preserving more information, however, the proposed RMLP is developed for color image fusion. RMLP is utilized to fuse the multi-focus images on R, G and B channels of RGB color image, respectively. Then an all-in-focus image is synthesized from the fused results of three channels. In this section, our proposed approach is evaluated and compared with the others in captured two datasets by a microscope.

### Datasets

Two datasets (S1 and S2 Datasets) are collected including fifteen defocused images of the top of a bullet and twelve defocused images of a bee’s body, respectively. The S1 Dataset is composed of image sequence that is captured at various depths by the microscope. The S2 Dataset is a sequence of synthetic images. Both S1 and S2 Datasets are used for subjective evaluation and objective evaluation respectively. As photographing equipment, the maximum magnification of the microscope of magnification 200 is used, which is made from the objective of the magnification 10 and the eyepiece of the magnification 20. The two datasets belong in the typical applications of forensic and biological fields.

### Subject evaluation

**Fig 5:**
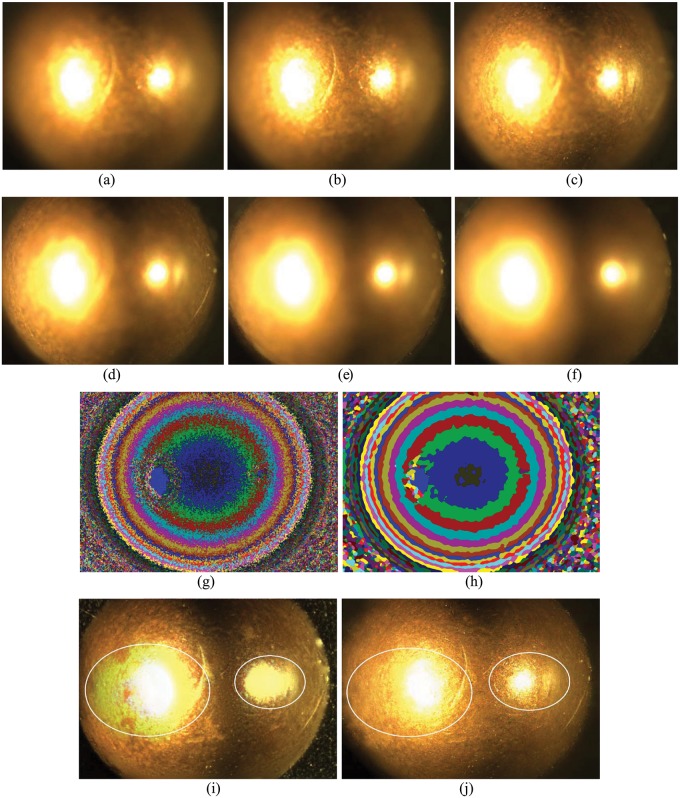
Illustration of the S1 Dataset and its fusion results. (a)-(f) are six random sampled examples from fifty multi-focus images, (g) is the mask image with only EOF measurement, (h) is the mask image with the proposed DBRG segmentation algorithm. (i) is the fusion result of the Laplacian pyramid (LP) method [11] and (j)is the fusion result of the proposed RMLP.

Fig 5(a)–5(f) shows six samples of the first dataset, which are the image sequence under different focus parameters of the bullet. For the depth variation of the ‘top of bullet’ object, each image has only a stripe region in focus as indicated by a color stripe in the mask image (Fig 5(h)). When the pixel level focus measurement is used to calculate focus pixels, it can be seen in Fig 5(g) that the mask image is noisy and have many errors.

**Fig 6:**
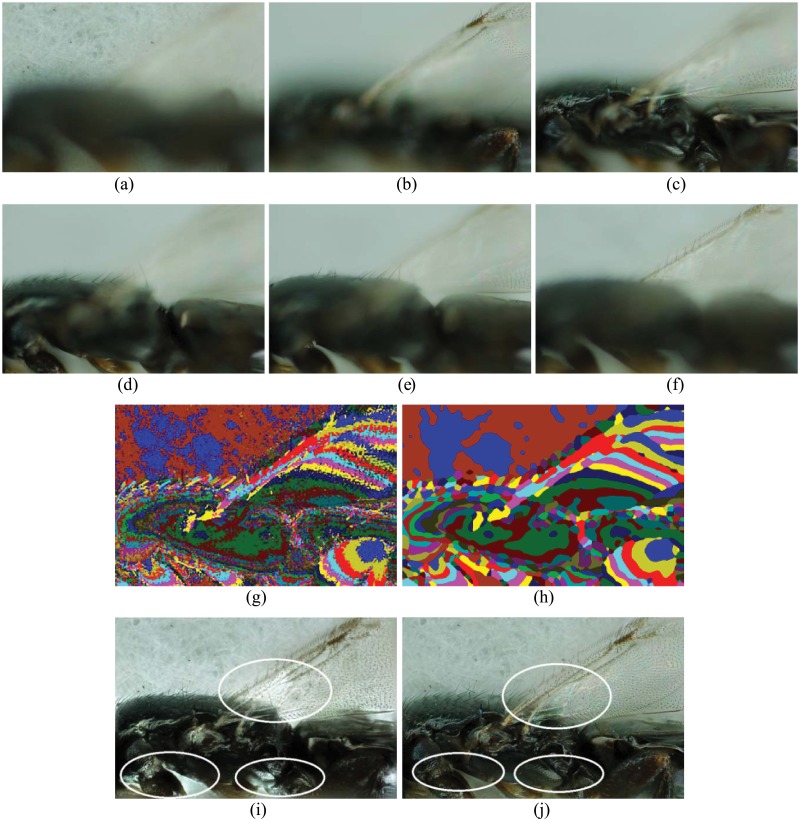
Illustration of the S2 Dataset and its fusion results. (a)-(f) are six random sampled examples from sixty multi-focus images, (g) is the mask image with only EOF measurement, (h) is the mask image with the proposed DBRG segmentation algorithm. (i) is the fusion result of the Laplacian pyramid (LP) method and (j)is the fusion result of the proposed RMLP.

When the DBRG algorithm of a proper *R* (see Eq 5) parameter is employed, clear focus regions are segmented, as shown in Fig 5(h). In Fig 5(i) and 5(j) the fusion results of the Laplacian Pyramid (LP) method [11] and the proposed RMLP approach are compared. It can be seen that in the highlighted regions with two circles in Fig 5(i) and 5(j), the fusion result of LP method has some color distortion, while the result of RMLP approach only has minimal distortion which validates the effectiveness of the proposed region mosaicing strategy in reducing distortion. Like Figs 5 and 6 shows also six samples of the second dataset with more complex texture feature and 3D shape. The results and comparison in Fig 6(i) and 6(j) demonstrate the performance of the RMLP approach over pixel level LP approach.

### Objective evaluation

For objective evaluation, the images of the second dataset are fused and computed the difference between fused result and the ground-truth. The fusion precisions are evaluated by Root Mean Squared Error (RMSE) and Structural Similarity (SSIM) [43] [44] [45]. RMSE is defined as

$$\begin{array}{c} RMSE={\left(\frac{\sum_{x=1}^{X}\sum_{y=1}^{Y}{\left(I(x,y)-I^{\prime }(x,y)\right)}^{2}}{X\cdot Y}\right)}^{\frac{1}{2}}. \end{array}$$ (13)

Where *X* and *Y* are the width and height of the fused image, respectively. *I*(*x*, *y*) denotes the value of the pixel (*x*, *y*) in the fused image and *I*′(*x*, *y*) is the ground-truth of the pixel (*x*, *y*) as the reference value. RMSE is inversely proportional to the performance of the fusion algorithm. If a perfect ‘all-in-focus’ image is achieved, the RMSE will be close to zero.

SSIM can be calculated as:

$$\begin{array}{c} SSIM=\frac{\left(2\mu_{g}\mu_{f}+C_{1})(2\sigma_{gf}+C_{2}\right)}{\left(\mu_{g}^{2}+\mu_{f}^{2}+C_{1})(\sigma_{g}^{2}+\sigma_{f}^{2}+C_{2}\right)}. \end{array}$$ (14)

Where *μ*_*g*_ denotes the mean intensity of all pixels in a sliding window of the ground-truth, *μ*_*g*_ denotes the mean intensity of all pixels in corresponding window of fused image, *σ*_*g*_ and *σ*_*f*_ are the statistical dispersions of all pixels the two windows, *σ*_*gf*_ is their covariance, *C*_1_ = (*K*_1_ × *L*) and *C*_2_ = (*K*_2_ × *L*) are two variables to stabilize the division with weak denominator. *K*_1_ and *K*_2_ are two constants, *L* is the dynamic range of the pixel values. In the experiments, *K*_1_, *K*_2_ and *L* are set to 0.01, 0.03 and 255 [44]. As a similarity measure, SSIM compares fused image and the ground-truth. A lager value of SSIM indicates that the result is more consist with the ground-truth.

**Fig 7:**
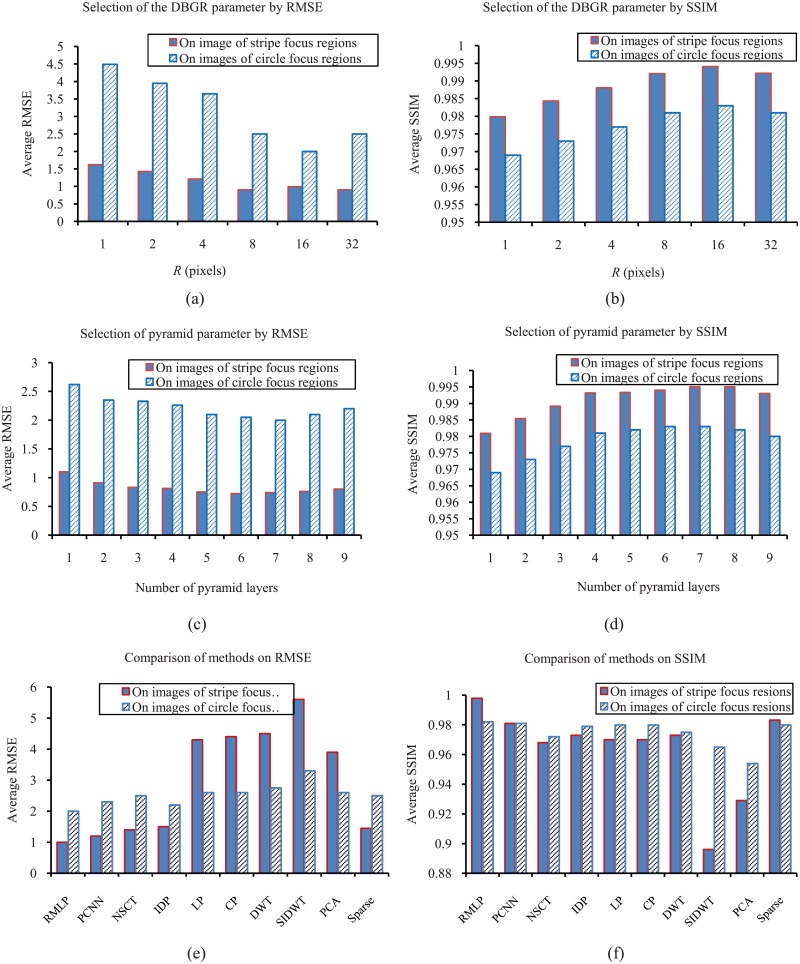
Illustration of quantitative evaluation (a) RMSE and (b) SSIM under different DBRG radius for the focus region mask segmentation. (c) RMSE and (d) SSIM with different pyramid layers. (e) and (f) are comparisons of nine methods.

In Fig 7 two types of mask image, stripe and circle, are used to fuse multi-focus image. In Fig 7(a) and 7(b), the results of RMSE and SSIM are used to determine the value of parameter *R*. It can be seen in Fig 7(a) that when *R* = 8 ∼ 16, the highest RMSE is obtained. It can be seen in Fig 7(b) that when *R* = 8, the largest SSIM is obtained. According to the above observations, *R* ∈ [8, 16] is selected as a fine turned parameter. It should be mentioned that the parameters are selected for an image with 720 × 480 pixels. It has already been observed in experiments that the value of *R* should be scaled proportional to the image size. A larger value of *R* not only reduces the accuracy but also delay the fusion process.

Fig 7(c) and 7(d) show that RMSE and SSIM have the lowest and highest values respectively if the pyramid has 6 or 7 layers. More or less layer cannot achieve optimizations for the fused results. Therefore it is crucial that we must set the appropriate number of layers for the objective quality of the fused image.

In Fig 7(e) and 7(f), RMLP is compared with six state of the art methods that include pulse coupled neural network (PCNN) [1], nonsubsampled contourlet transform (NSCT) [21], interactive digital photomontage (IDP) [32], Laplacian pyramid (LP) [11], the contrast pyramid (CP) [16], Discrete Wavelet Transform (DWT) [46], the Shift-invariant Discrete Wavelet Transform (SIDWT) [25], Principal Component Analysis(PCA) [24] and sparse reconstruction method [29]. PCNN is implemented according to the descriptions in [1]. The programs of other approaches are downloaded from the authors’ websites [47] [48].

In Fig 7(e), the RMSE of RMLP is the lowest comparing with other methods, which indicates that RMSE has highest precision for fused images. In Fig 7(f), both the fusion result of RMLP and ground-truth have highest similarity among seven approaches, which shows that RMLP retains the most information in the original image. To sum up, the proposed approach, RMLP, improves the performance of the state of the arts.

**Fig 8:**
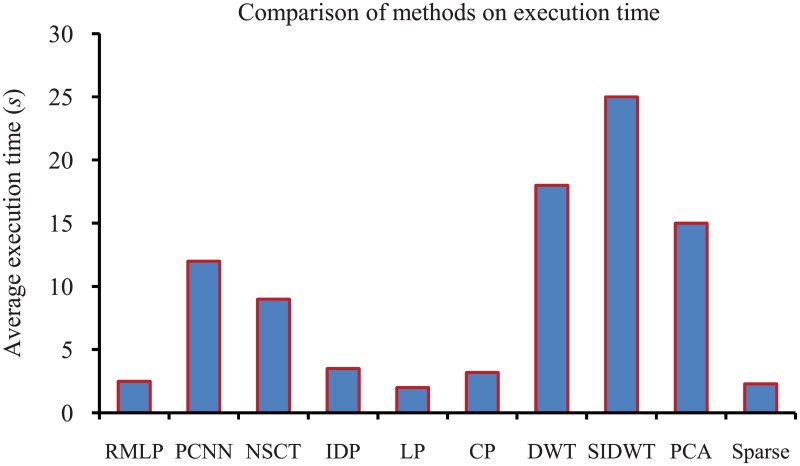
Average execution time of RMLP and other methods.

Considering the computation issue, the existing PCNN method has significantly higher computational complexity for iterative operations. By contrast, our RMLP approach is significantly simpler and more efficient. The average execution time is also compared, which is shown in Fig 8. The average execution time of PCNN approach is about 12.0 seconds to fuse an image of 720 × 480 pixels on an Intel Core i3-2130CPU of 3.4 GHz. The proposed approach, RMLP, however, spends just 1.35 seconds. In contrast, the IDP approach that uses a graph-cut optimal algorithm to calculate focus region mask spends 2.6 seconds on average.

## Conclusions

Pyramid based image fusion approaches, such as Wavelet or Contrast Pyramids, are classic methods for image fusion. When these methods are applied to multi-focus image fusion, the region continuity characteristics are not fully explored. In this paper we propose a new multi-focus image fusion approach, called RMLP, which explores unique characteristics of multi-focus imaging and transfers the classical pixel-based pyramid fusion to region-based. The performance of RMLP is evaluated in both objective and subjective datasets. The experiment results show that RMLP has lowest error and best robustness comparing with the existing methods.

A well-known problem of existing popular approaches is that all multi-focus images need to be aligned with each other. The miss-alignment of images may cause failure on the fusion results. In order to insure alignment, the multi-focus images of our data set were captured by a stable platform (a desktop microscope).

## Supporting information

S1 Dataset Image sequence captured at various depths by the microscope. (ZIP)

S2 Dataset Image sequence of synthetic images. (ZIP)
