# Supplementary figures and images for: A multi-focus image fusion method via region mosaicking on Laplacian pyramids (part 1 of 2)
Source: PLoS One. 2018 May 17;13(5):e0191085. doi: 10.1371/journal.pone.0191085 (PMC5957432; doi:10.1371/journal.pone.0191085)

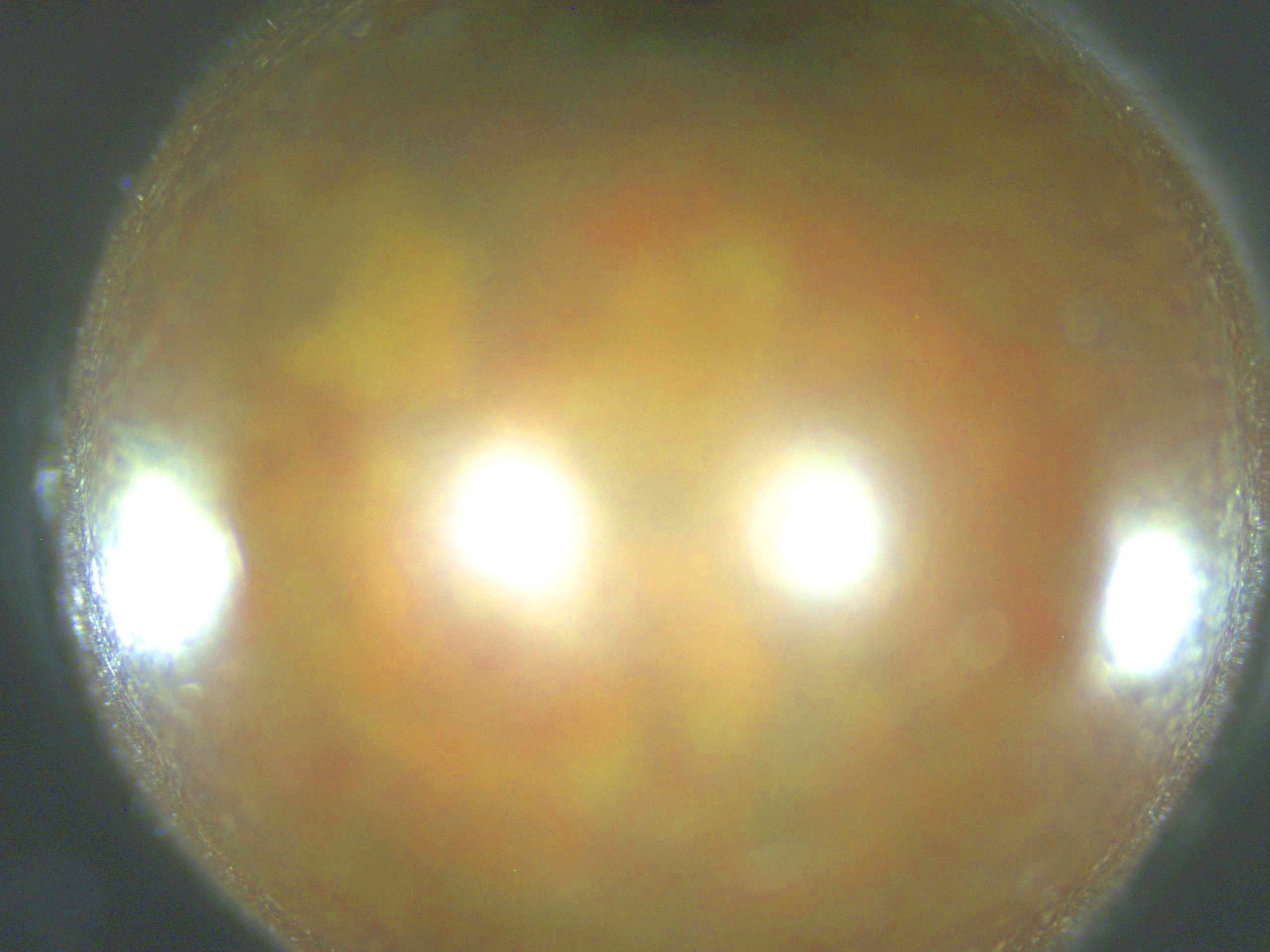

Supplement: S1 Dataset — (ZIP) [file pone.0191085.s001.zip › data set 1/FD138.jpg]

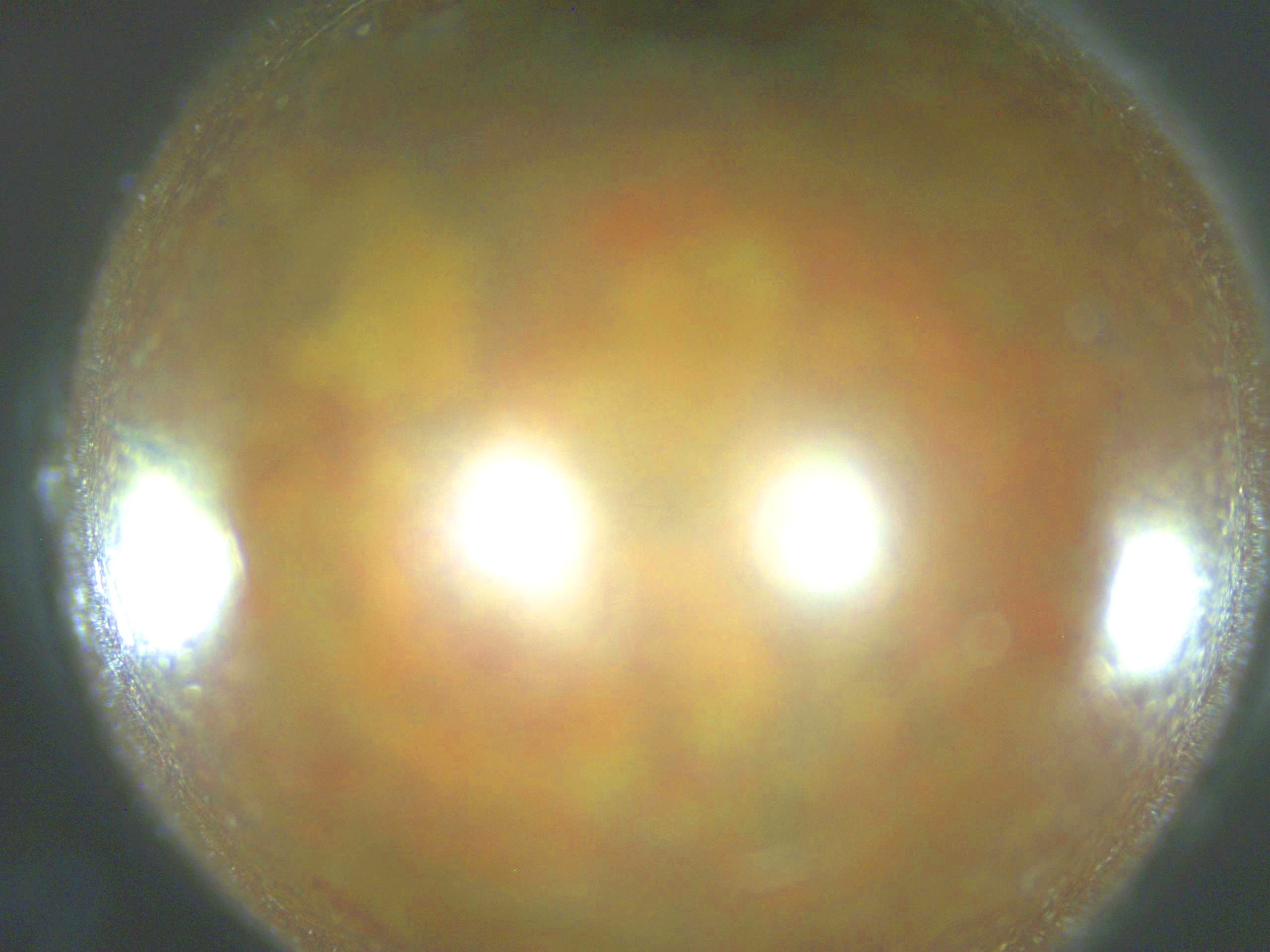

Supplement: S1 Dataset — (ZIP) [file pone.0191085.s001.zip › data set 1/FD139.jpg]

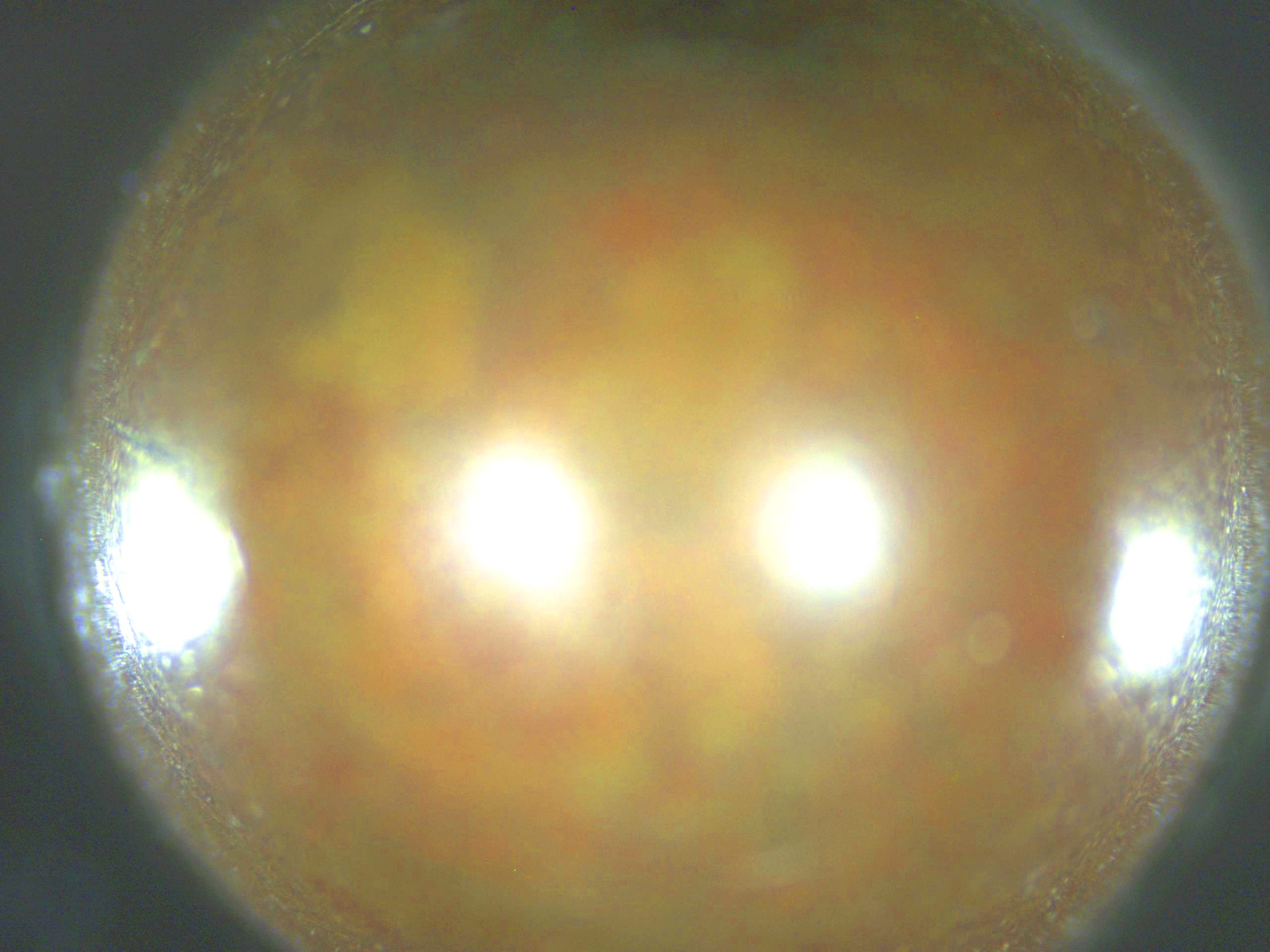

Supplement: S1 Dataset — (ZIP) [file pone.0191085.s001.zip › data set 1/FD140.jpg]

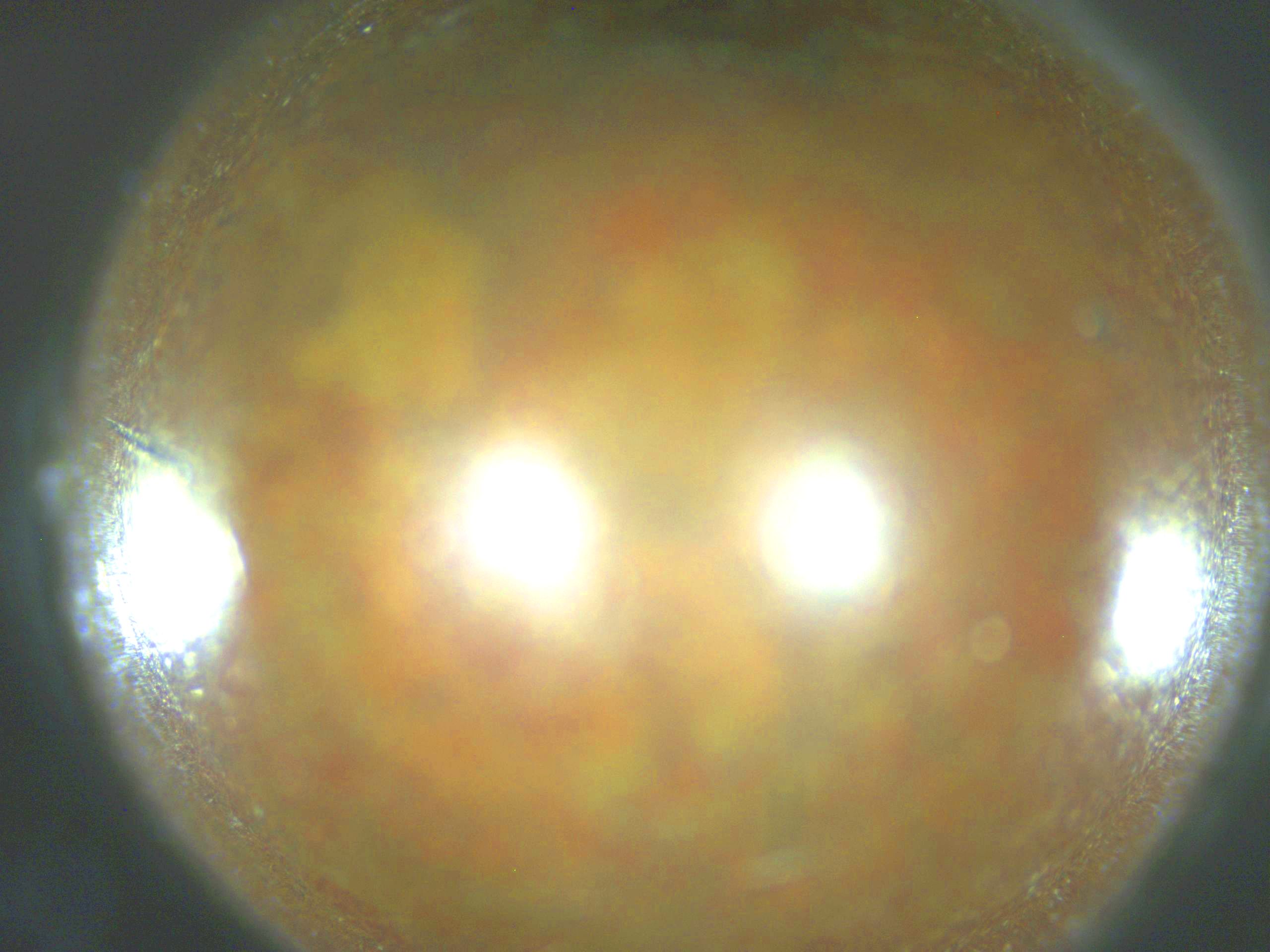

Supplement: S1 Dataset — (ZIP) [file pone.0191085.s001.zip › data set 1/FD141.jpg]

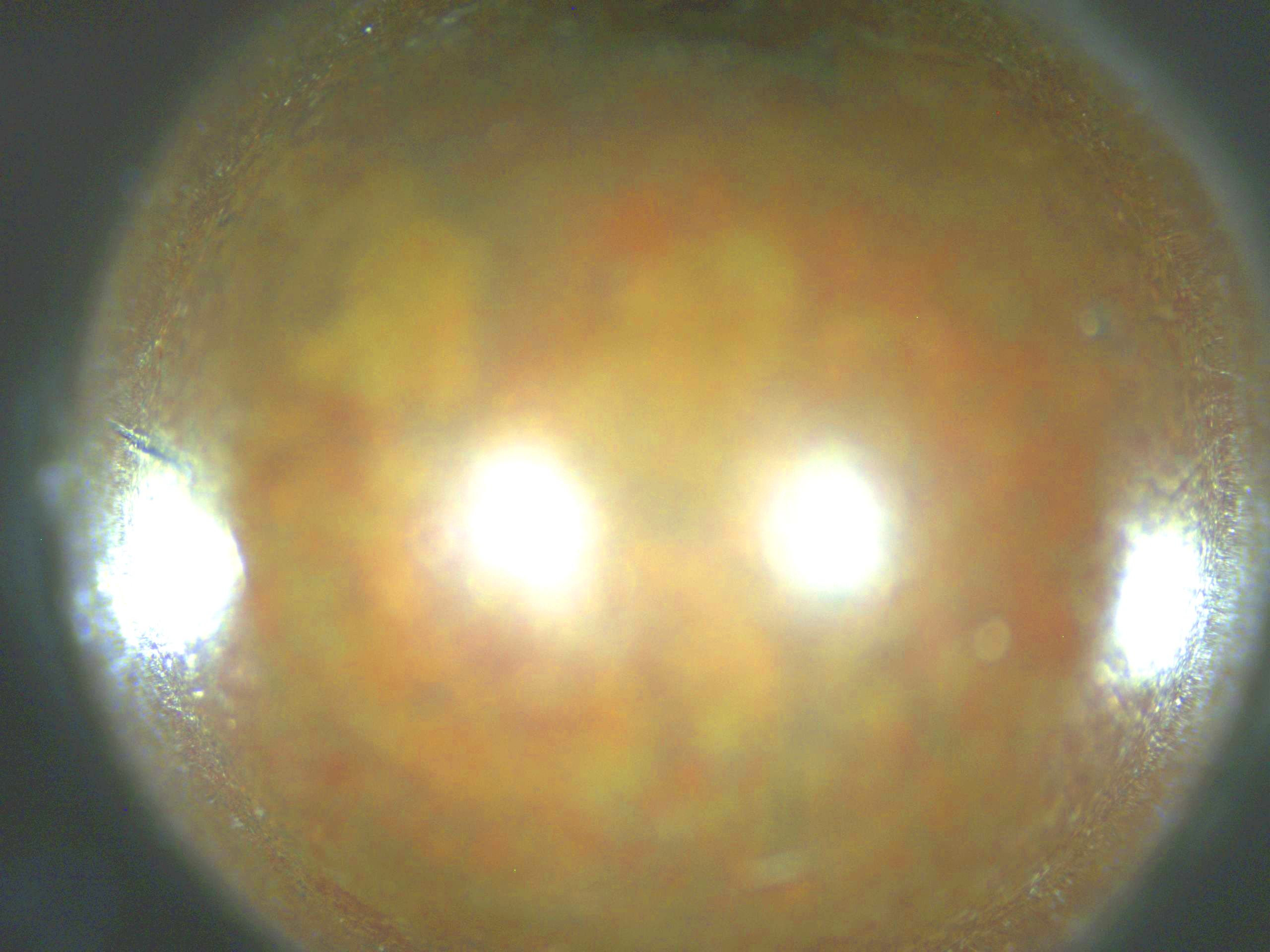

Supplement: S1 Dataset — (ZIP) [file pone.0191085.s001.zip › data set 1/FD142.jpg]

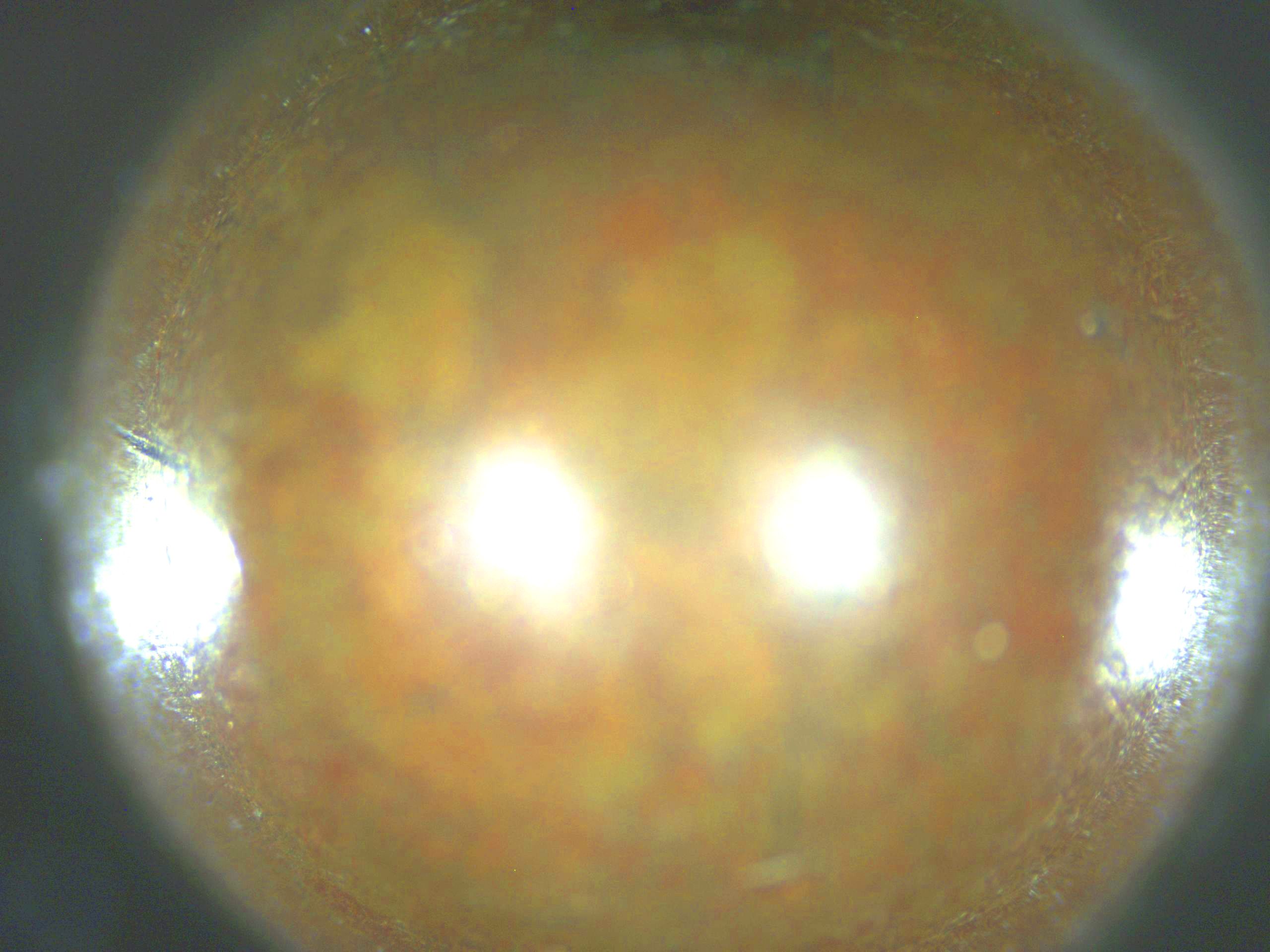

Supplement: S1 Dataset — (ZIP) [file pone.0191085.s001.zip › data set 1/FD143.jpg]

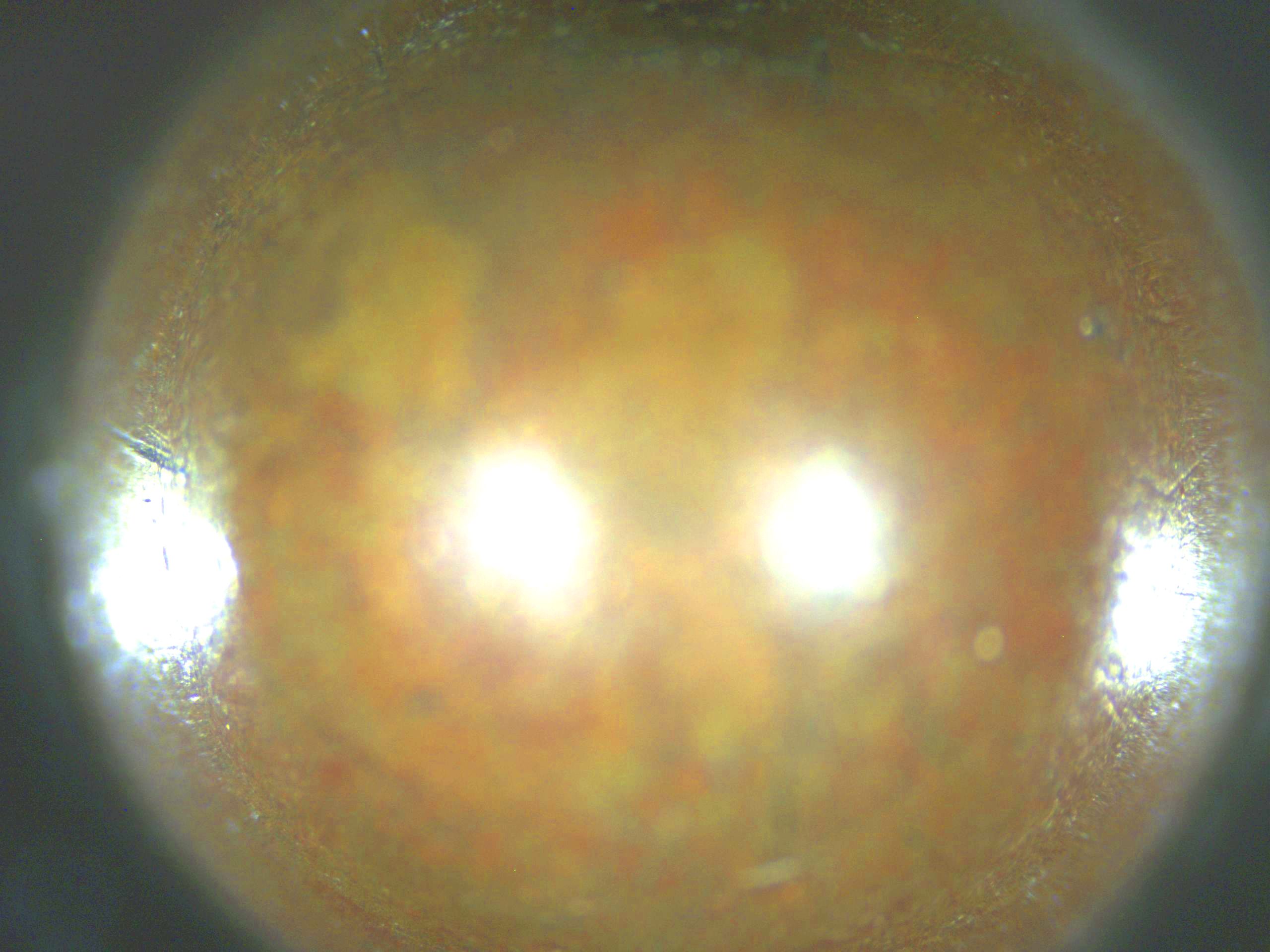

Supplement: S1 Dataset — (ZIP) [file pone.0191085.s001.zip › data set 1/FD144.jpg]

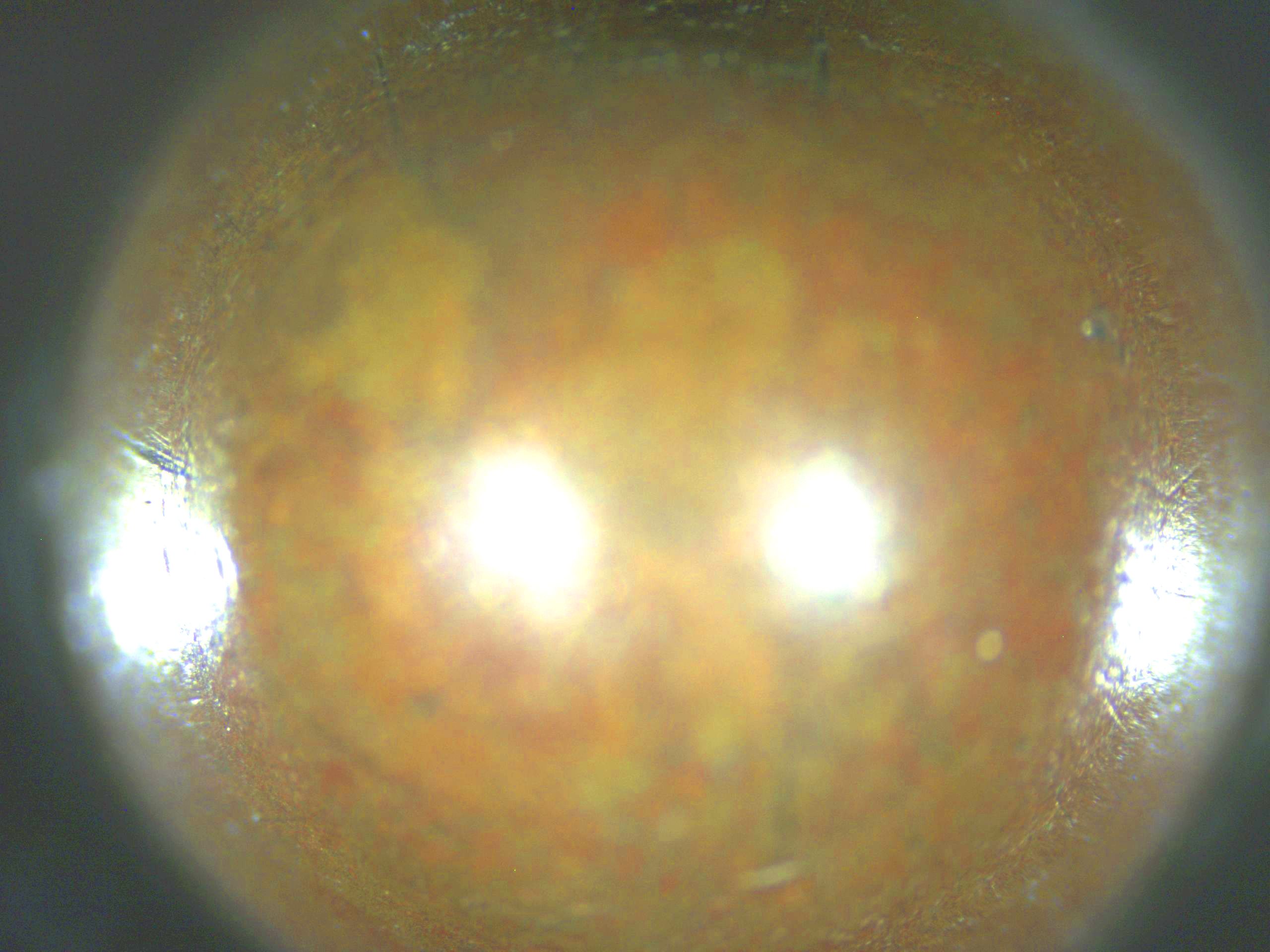

Supplement: S1 Dataset — (ZIP) [file pone.0191085.s001.zip › data set 1/FD145.jpg]

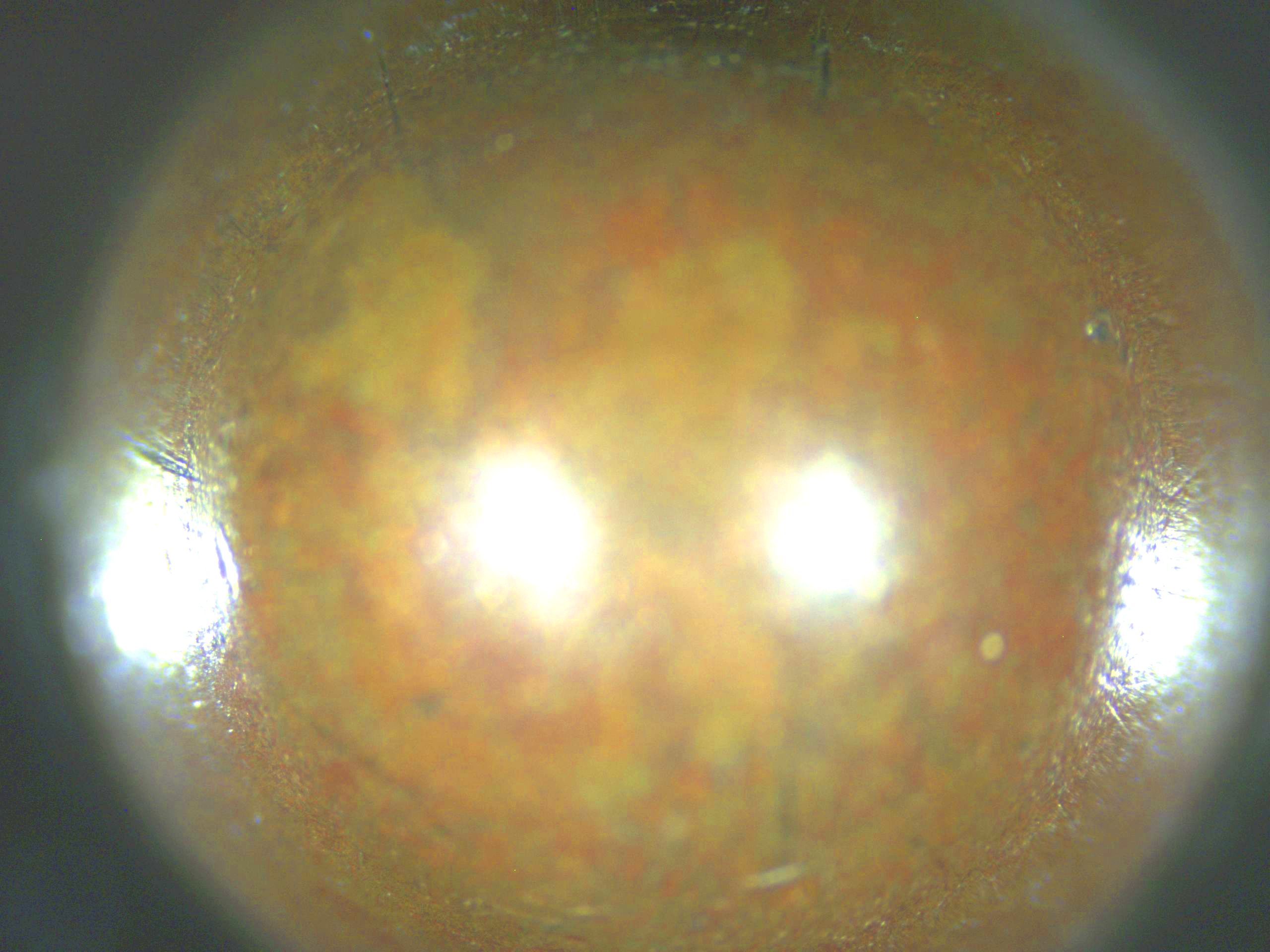

Supplement: S1 Dataset — (ZIP) [file pone.0191085.s001.zip › data set 1/FD146.jpg]

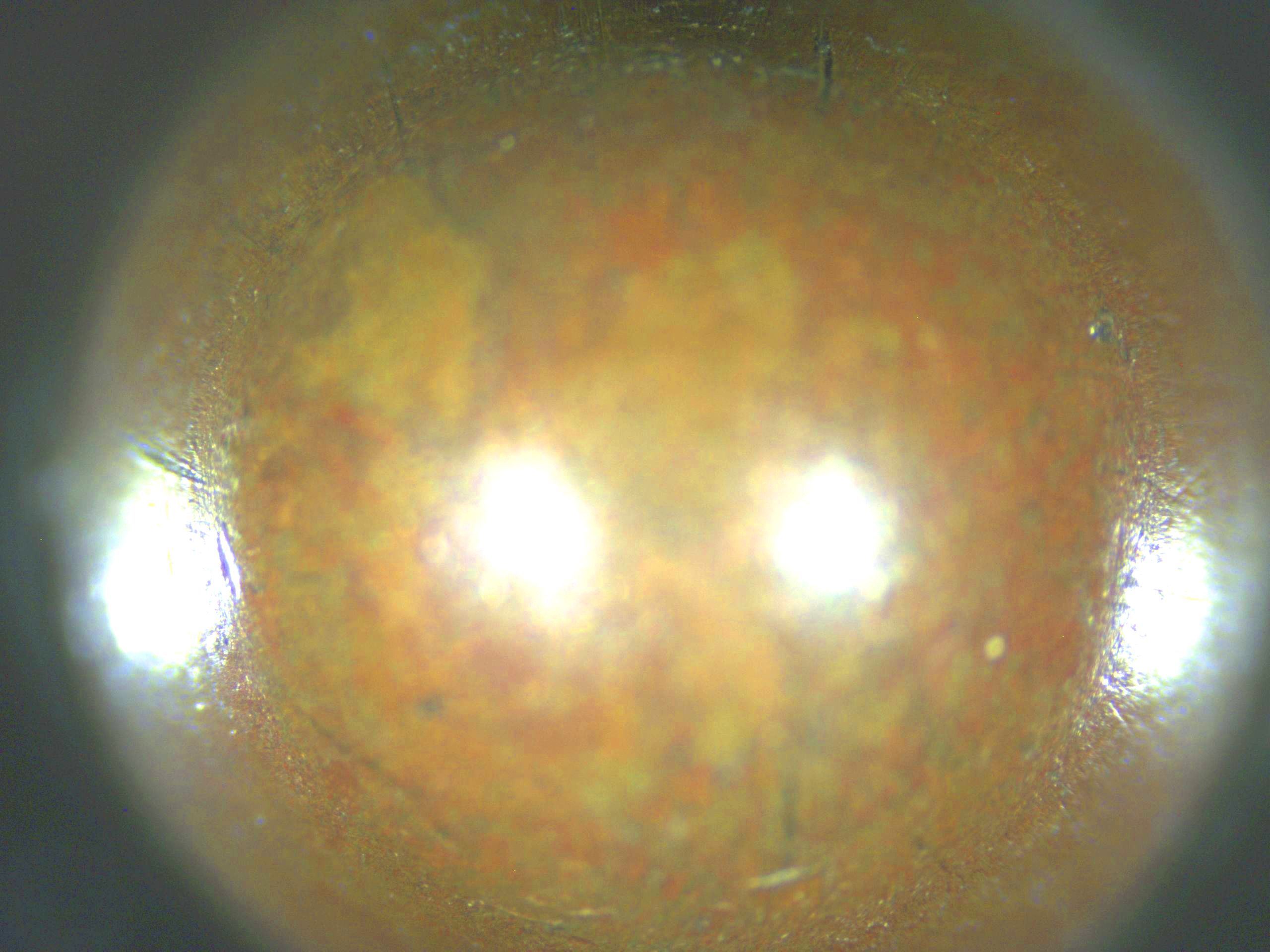

Supplement: S1 Dataset — (ZIP) [file pone.0191085.s001.zip › data set 1/FD147.jpg]

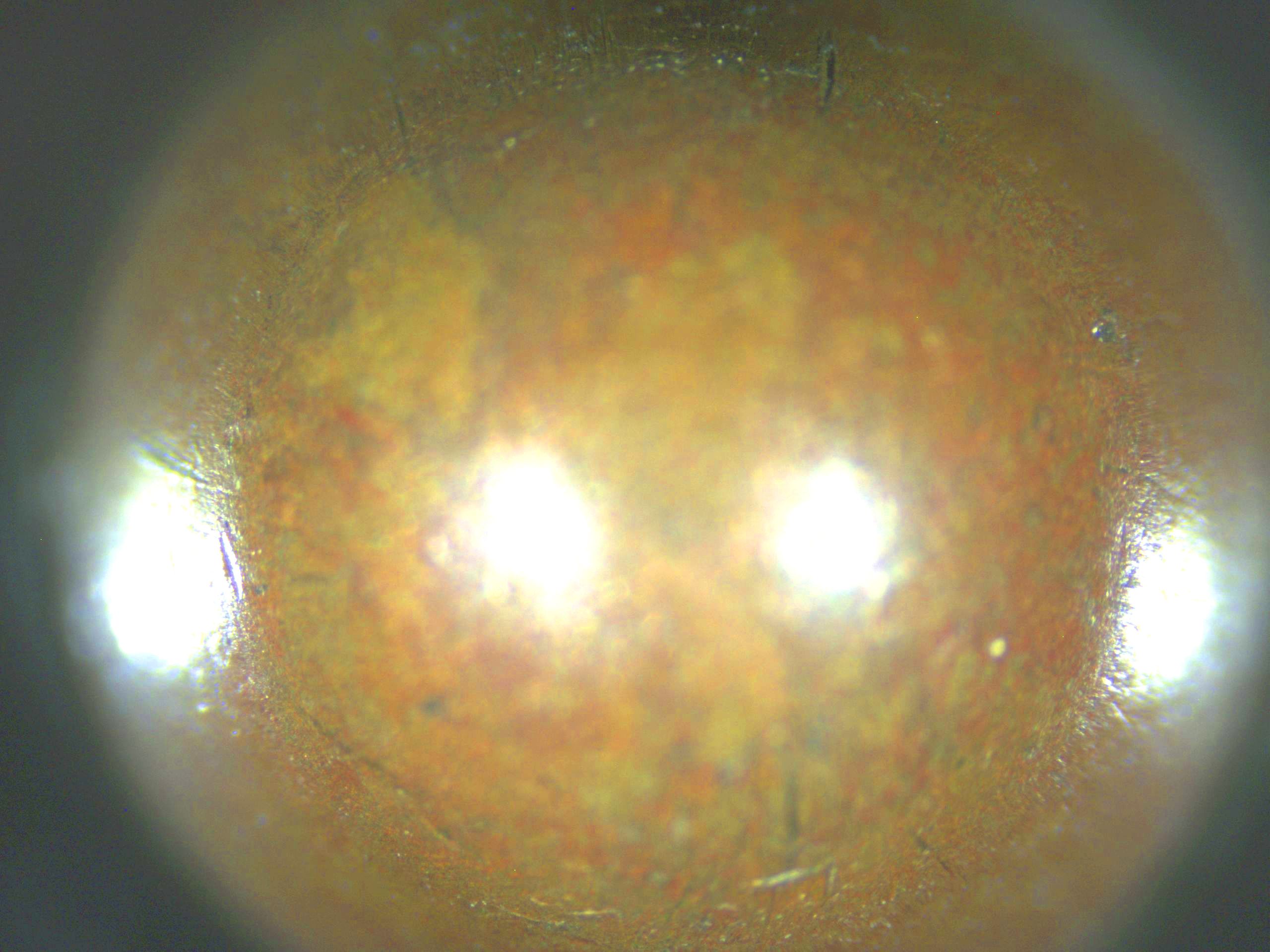

Supplement: S1 Dataset — (ZIP) [file pone.0191085.s001.zip › data set 1/FD148.jpg]

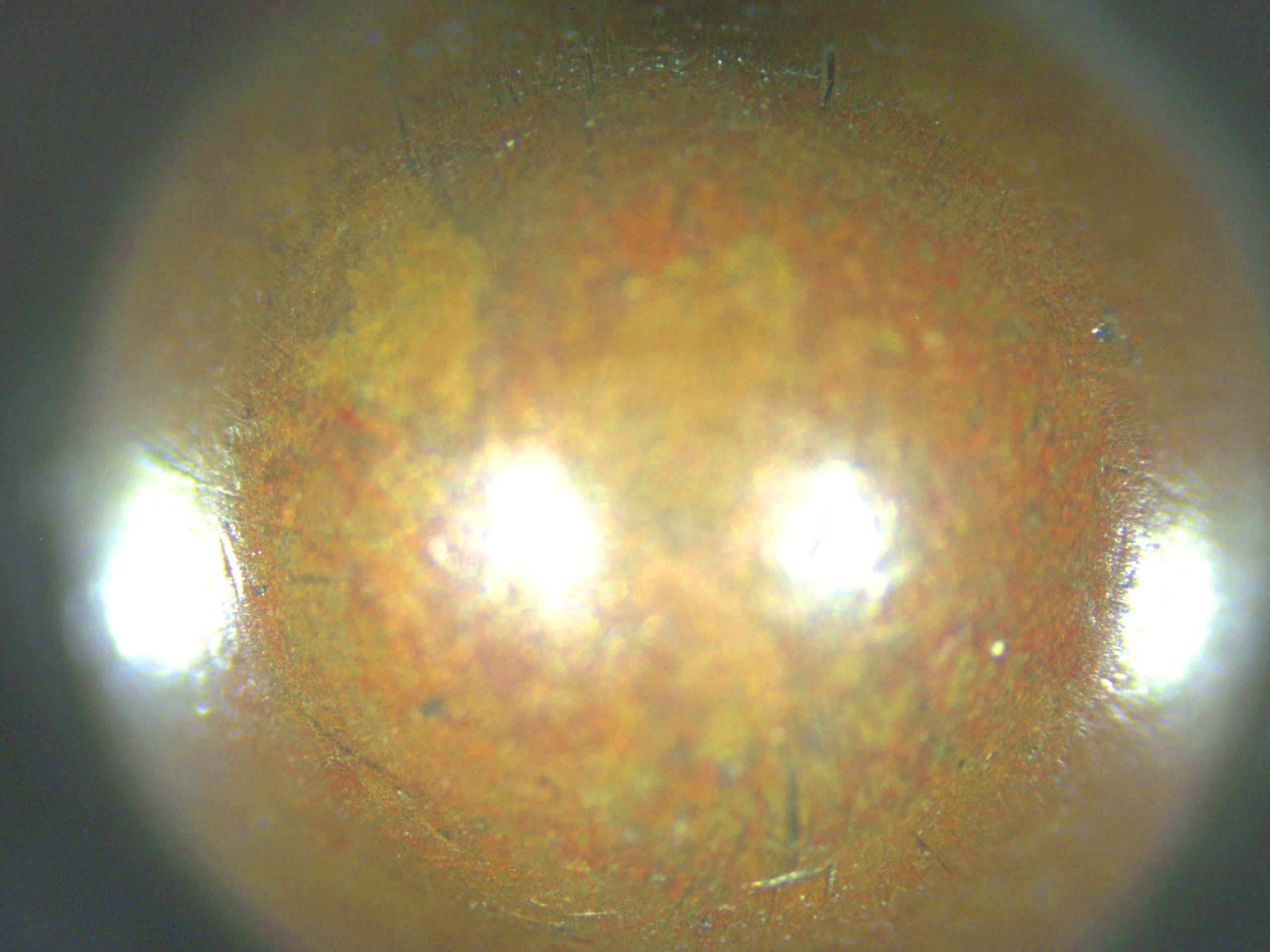

Supplement: S1 Dataset — (ZIP) [file pone.0191085.s001.zip › data set 1/FD149.jpg]

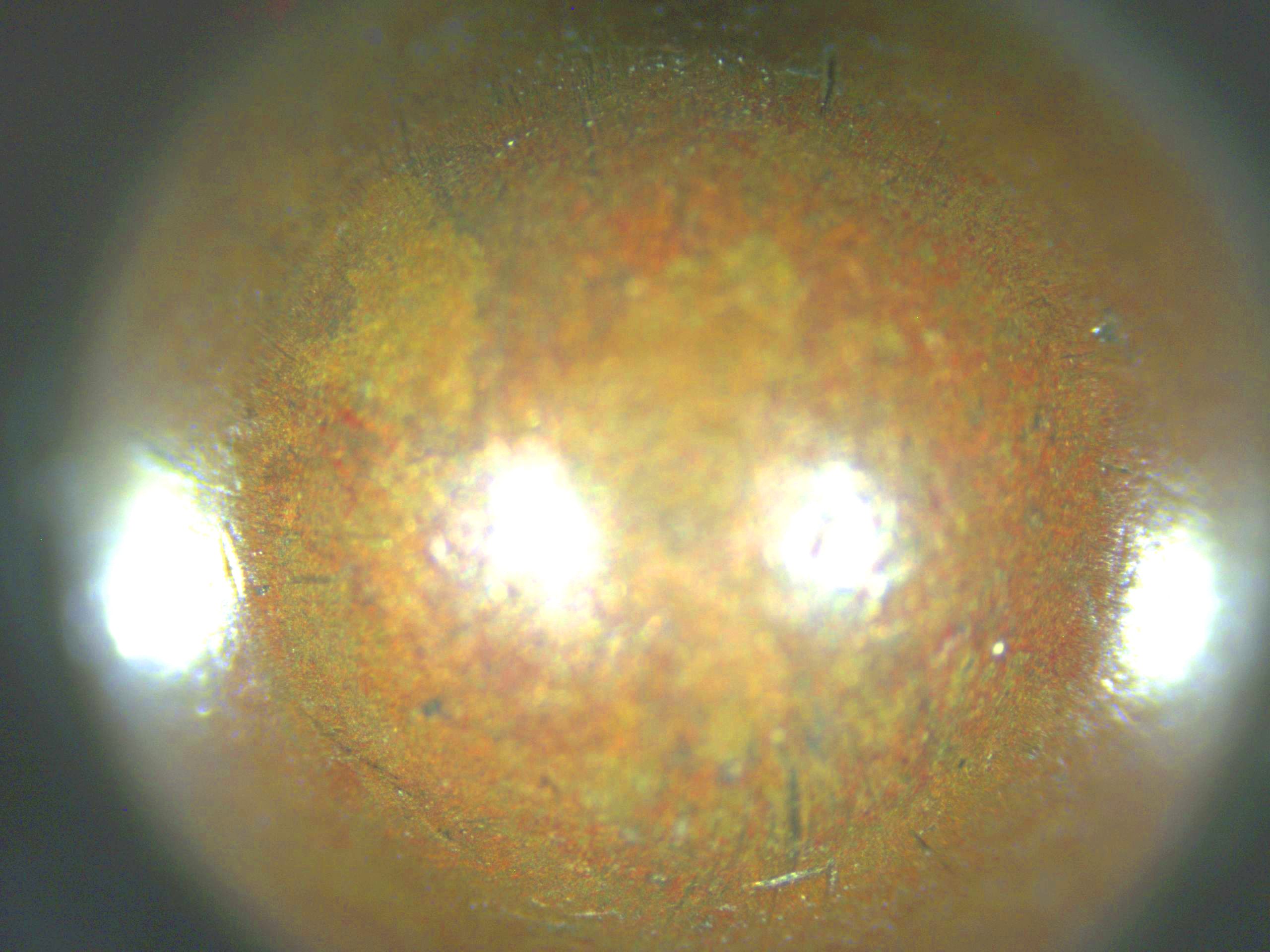

Supplement: S1 Dataset — (ZIP) [file pone.0191085.s001.zip › data set 1/FD150.jpg]

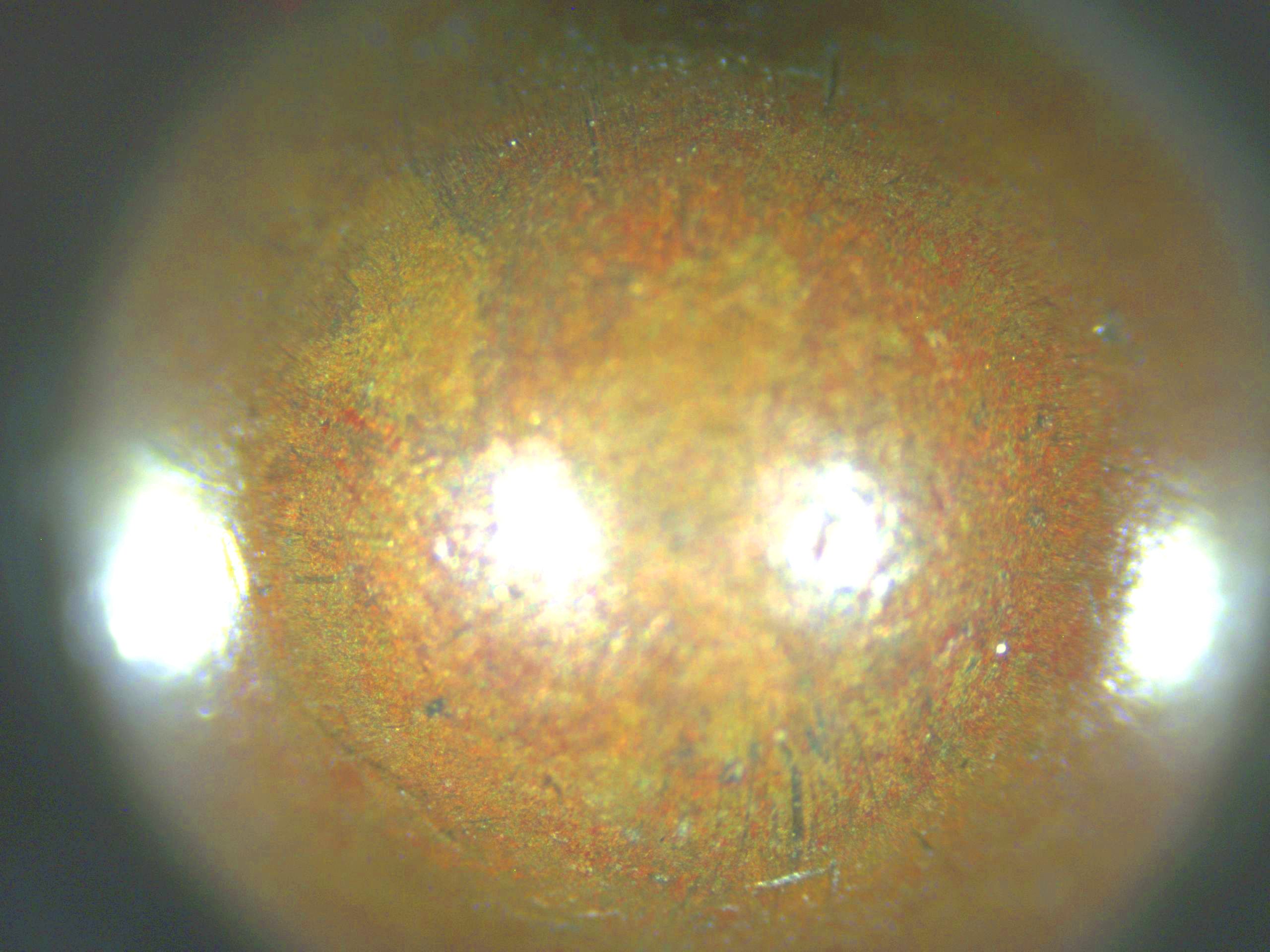

Supplement: S1 Dataset — (ZIP) [file pone.0191085.s001.zip › data set 1/FD151.jpg]

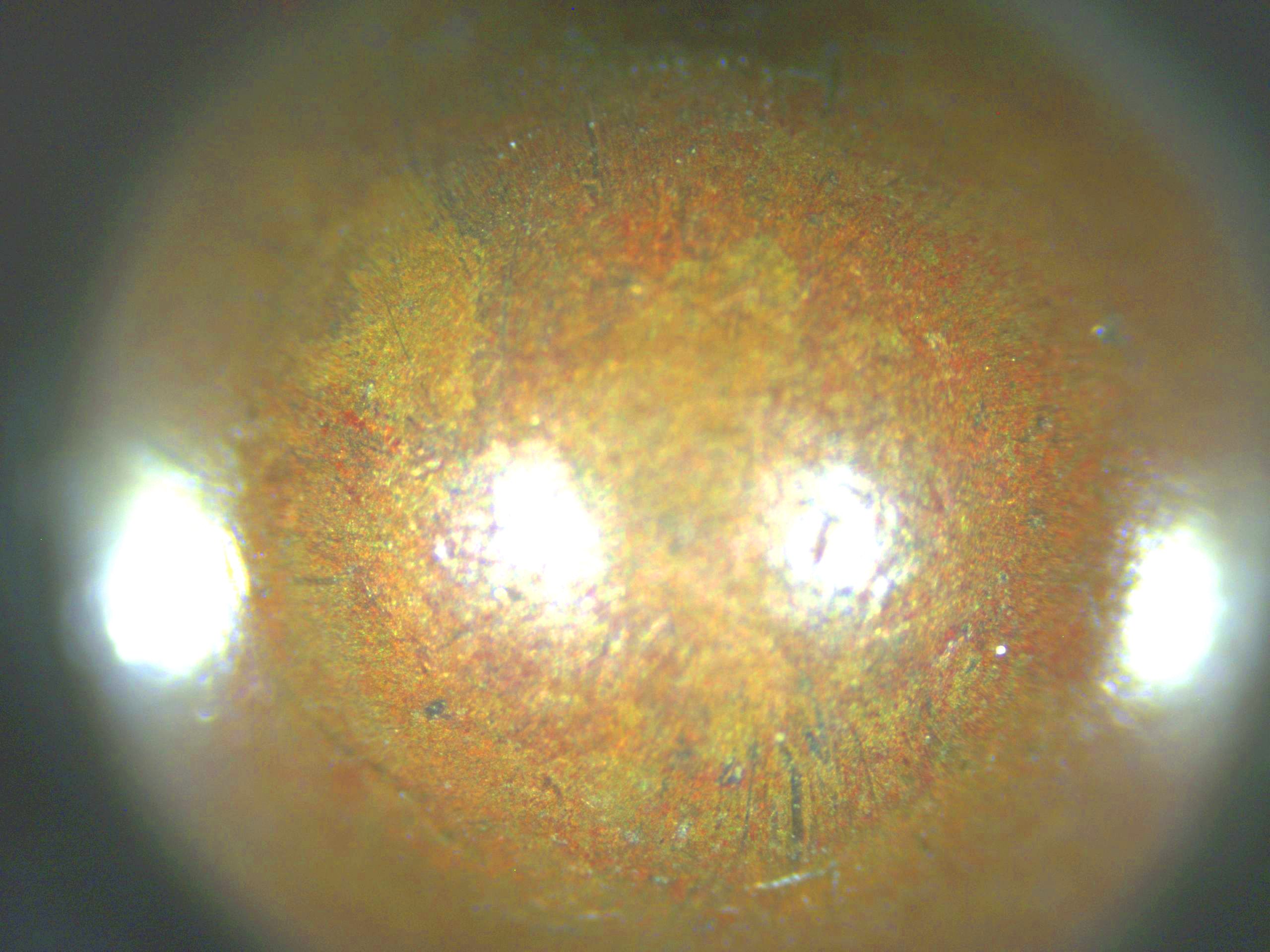

Supplement: S1 Dataset — (ZIP) [file pone.0191085.s001.zip › data set 1/FD152.jpg]

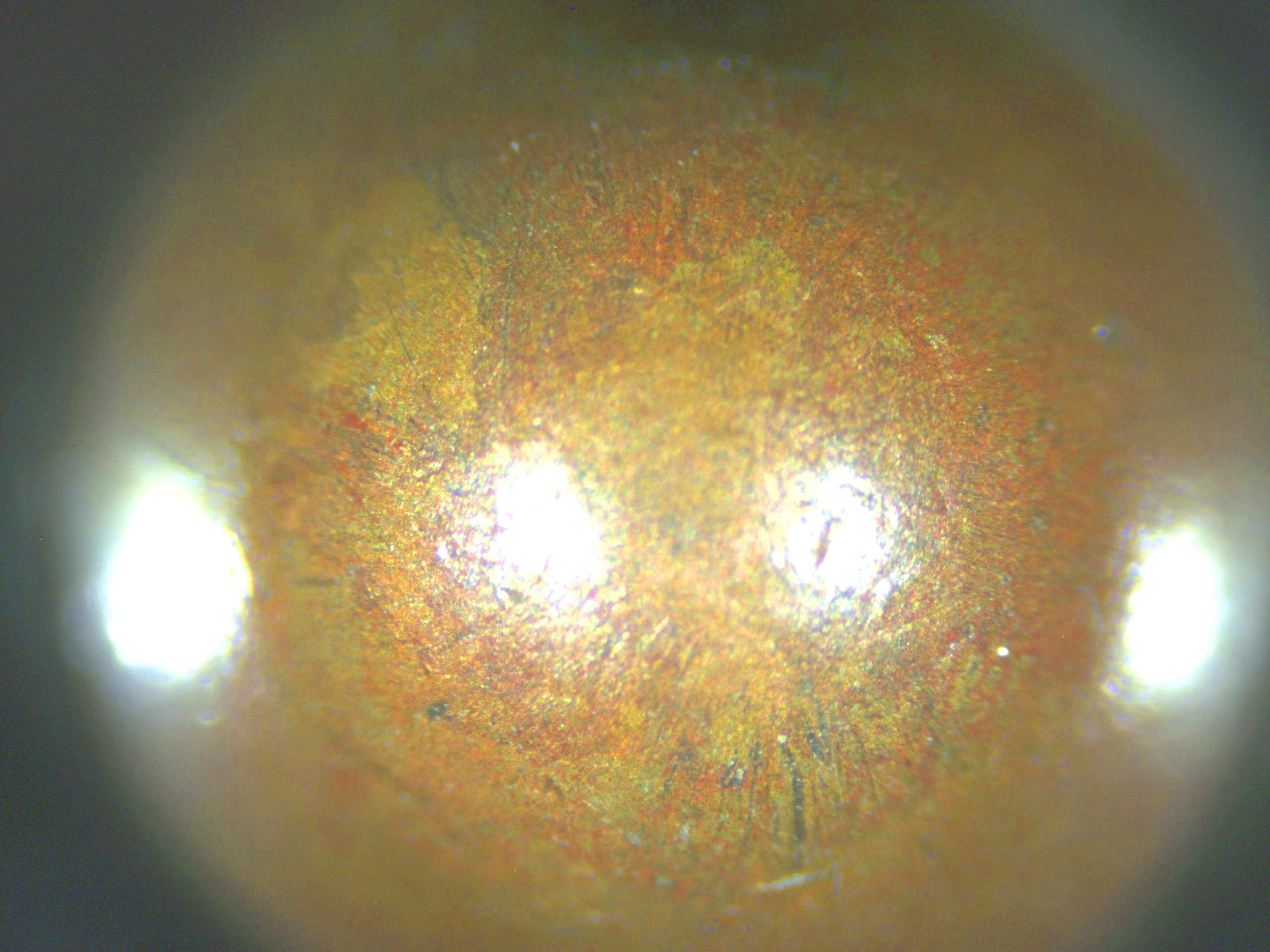

Supplement: S1 Dataset — (ZIP) [file pone.0191085.s001.zip › data set 1/FD153.jpg]

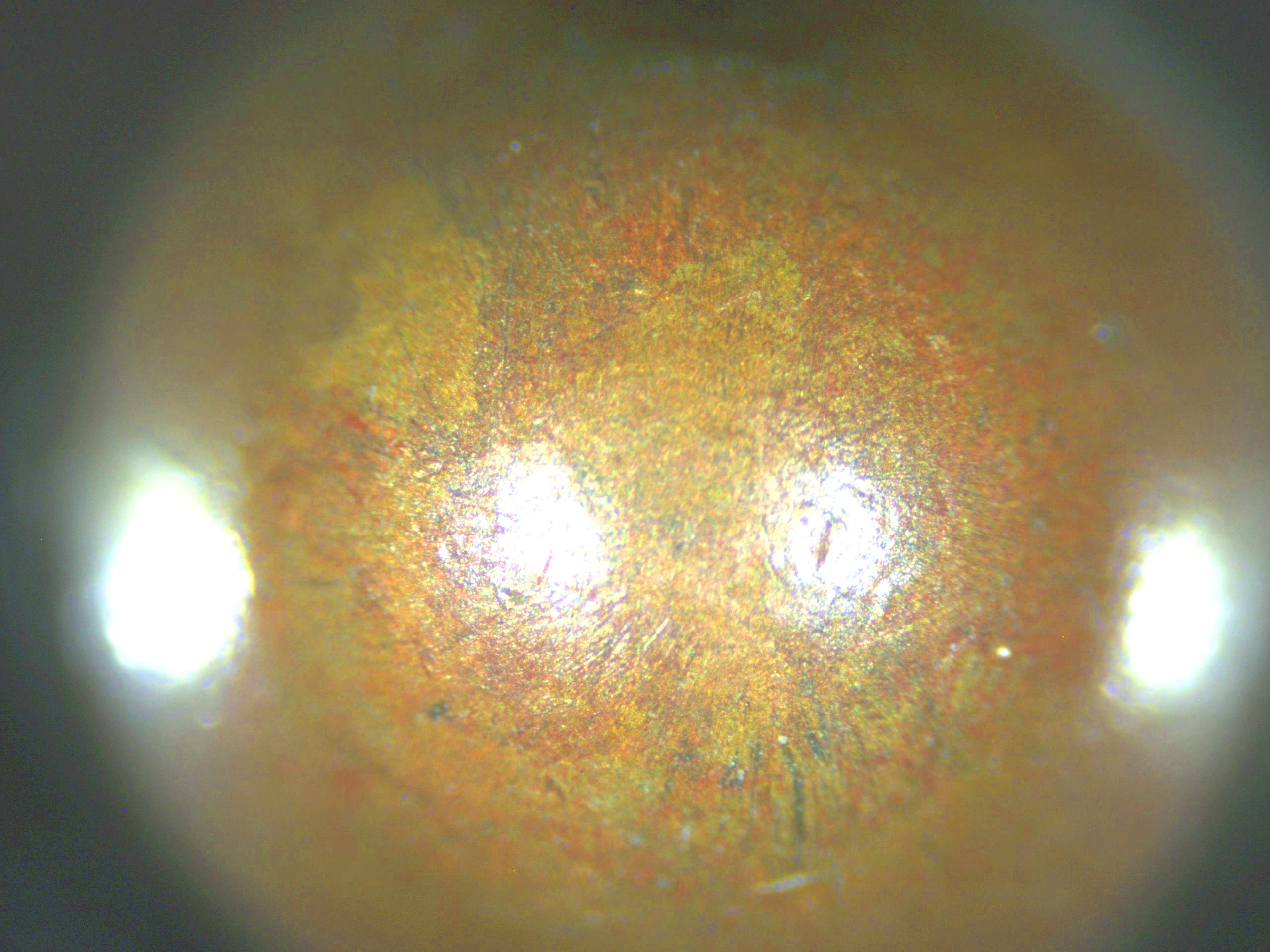

Supplement: S1 Dataset — (ZIP) [file pone.0191085.s001.zip › data set 1/FD154.jpg]

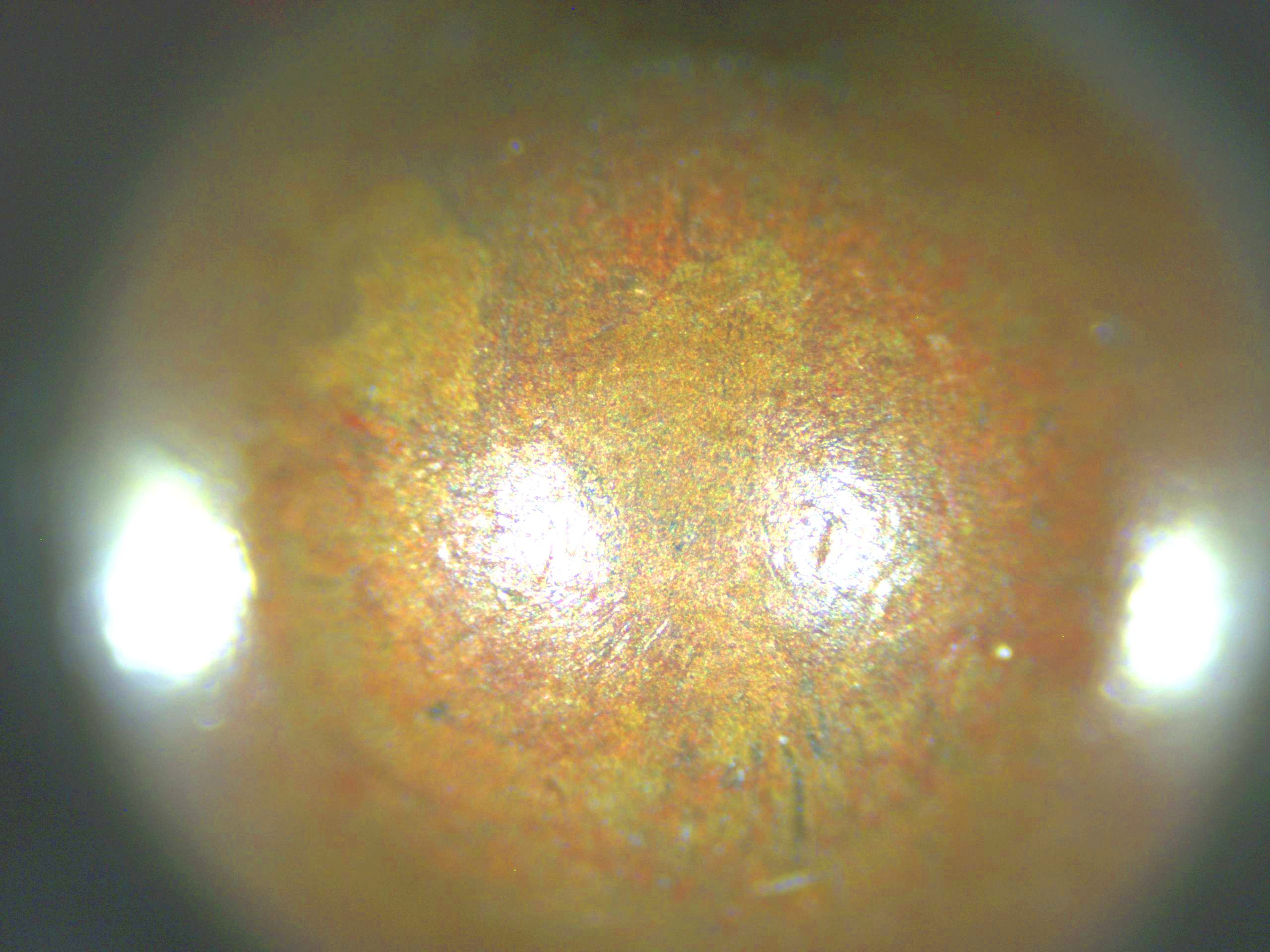

Supplement: S1 Dataset — (ZIP) [file pone.0191085.s001.zip › data set 1/FD155.jpg]

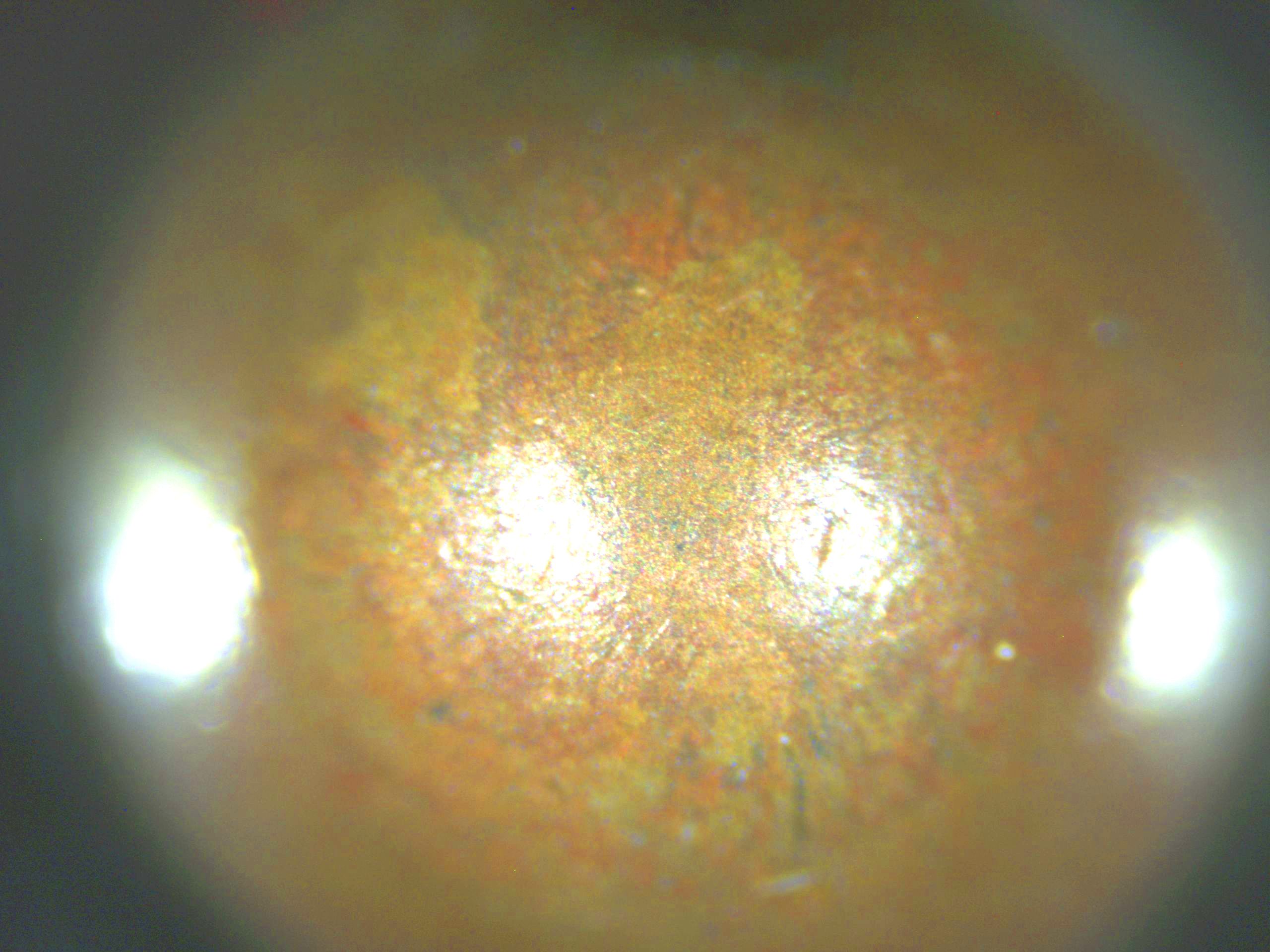

Supplement: S1 Dataset — (ZIP) [file pone.0191085.s001.zip › data set 1/FD156.jpg]

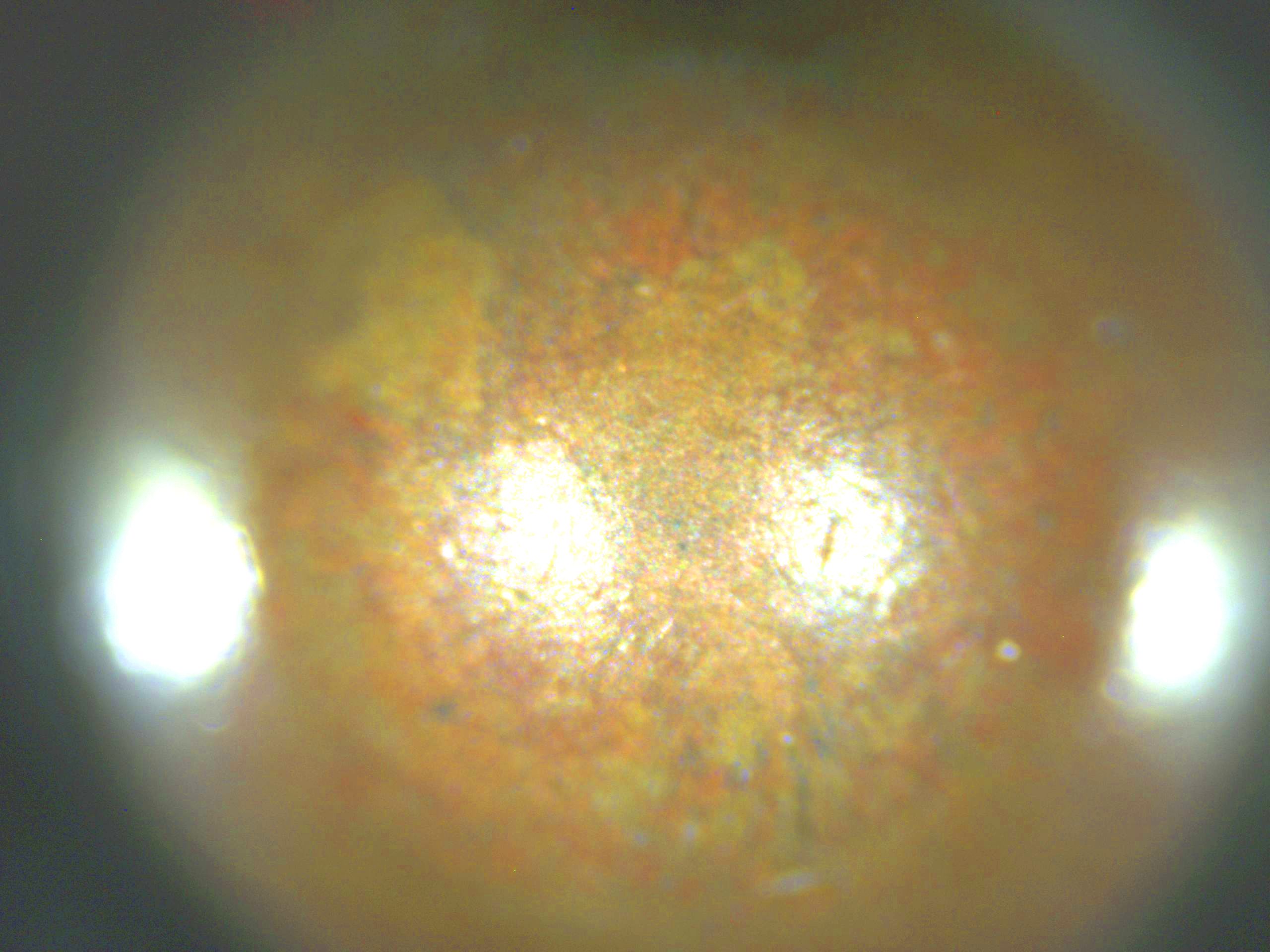

Supplement: S1 Dataset — (ZIP) [file pone.0191085.s001.zip › data set 1/FD157.jpg]

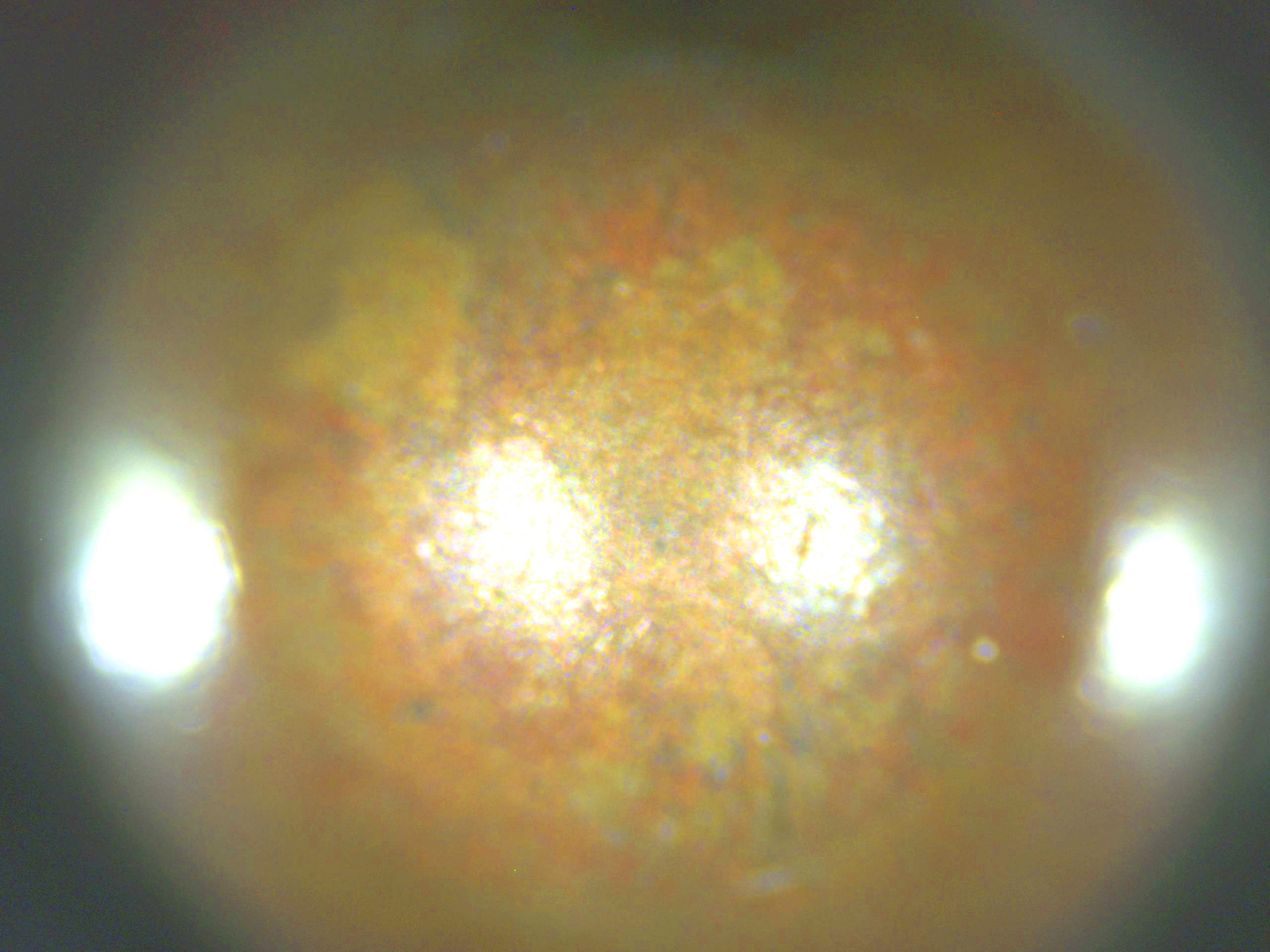

Supplement: S1 Dataset — (ZIP) [file pone.0191085.s001.zip › data set 1/FD158.jpg]

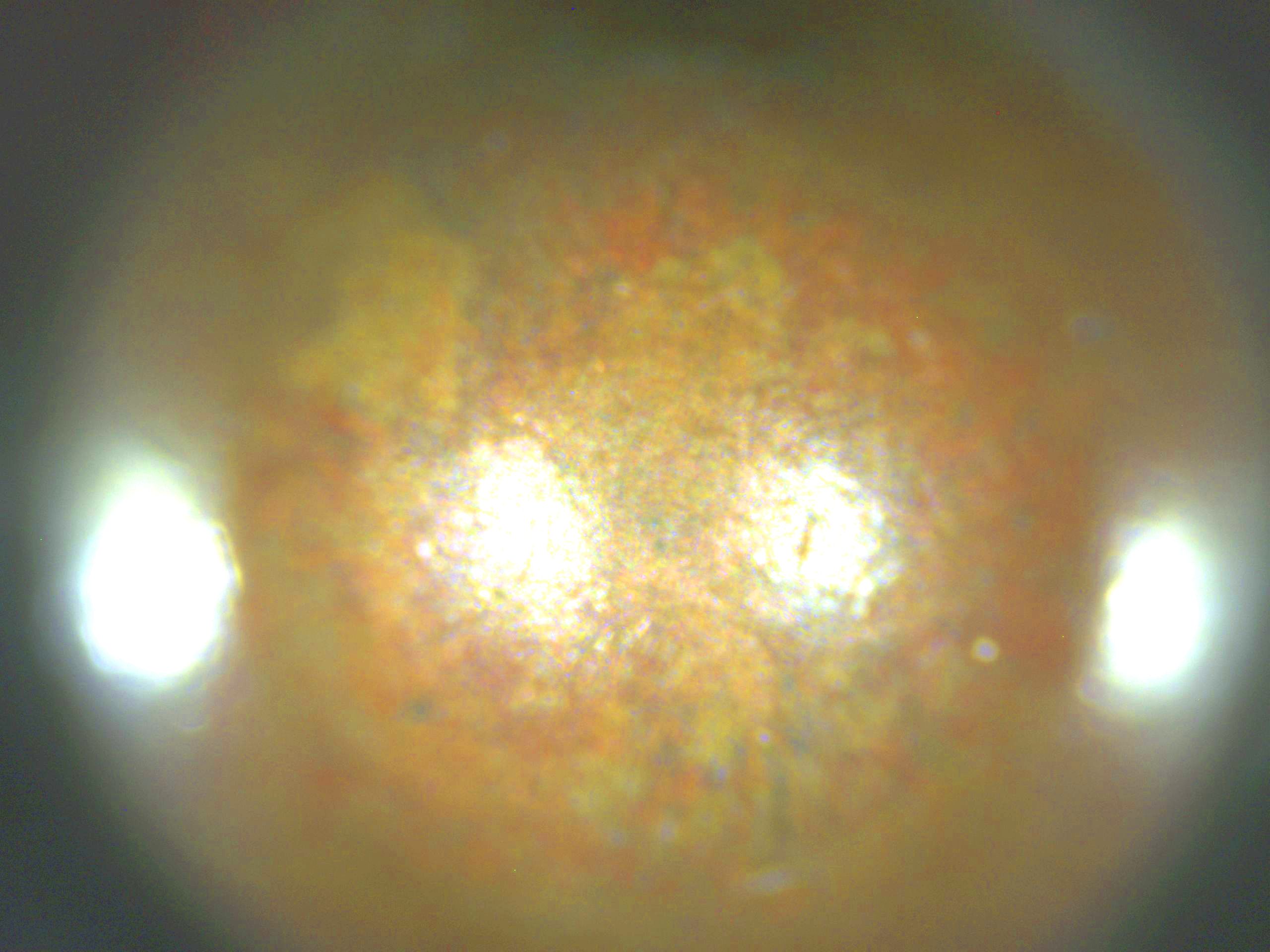

Supplement: S1 Dataset — (ZIP) [file pone.0191085.s001.zip › data set 1/FD259.jpg]

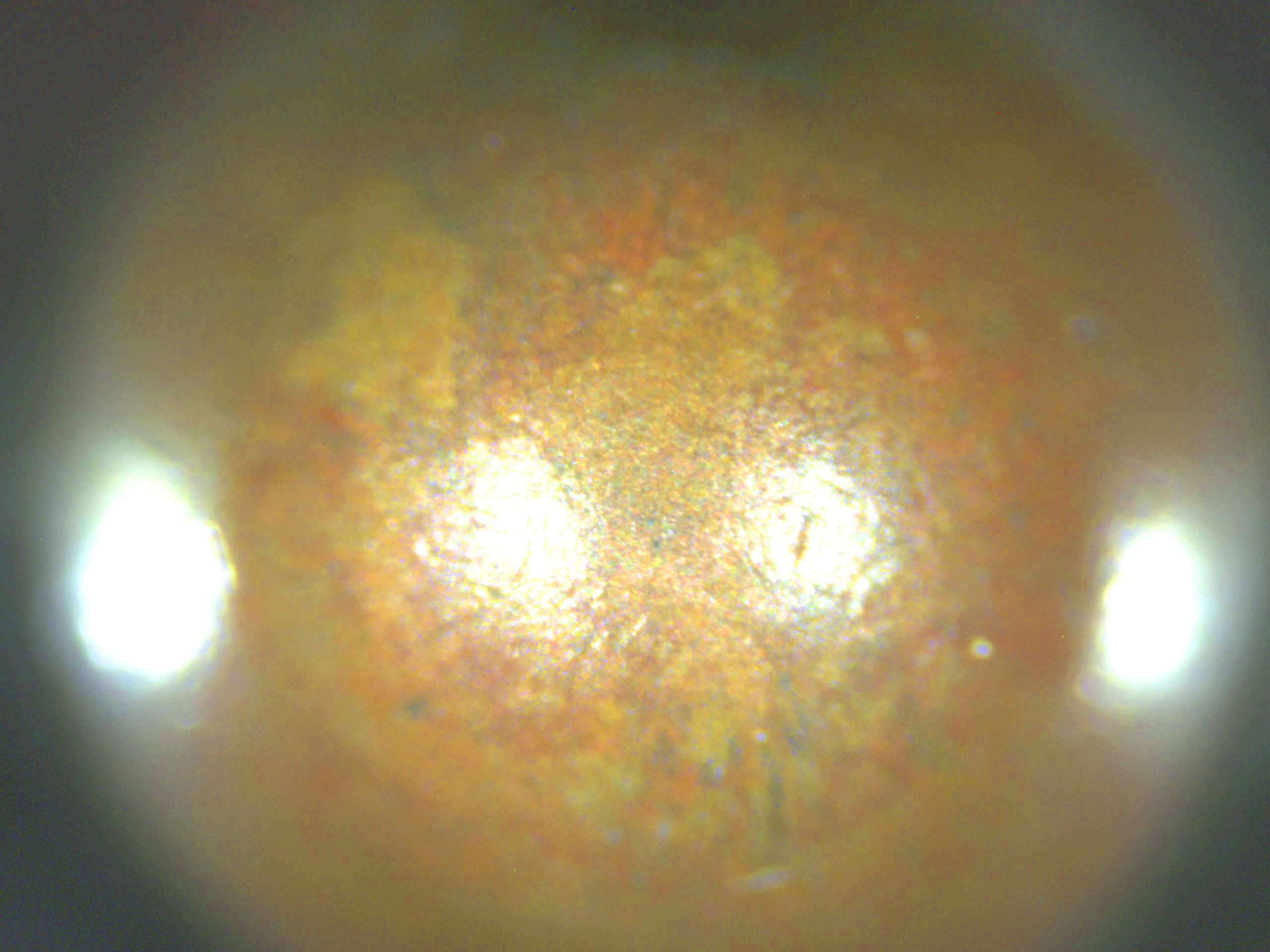

Supplement: S1 Dataset — (ZIP) [file pone.0191085.s001.zip › data set 1/FD260.jpg]

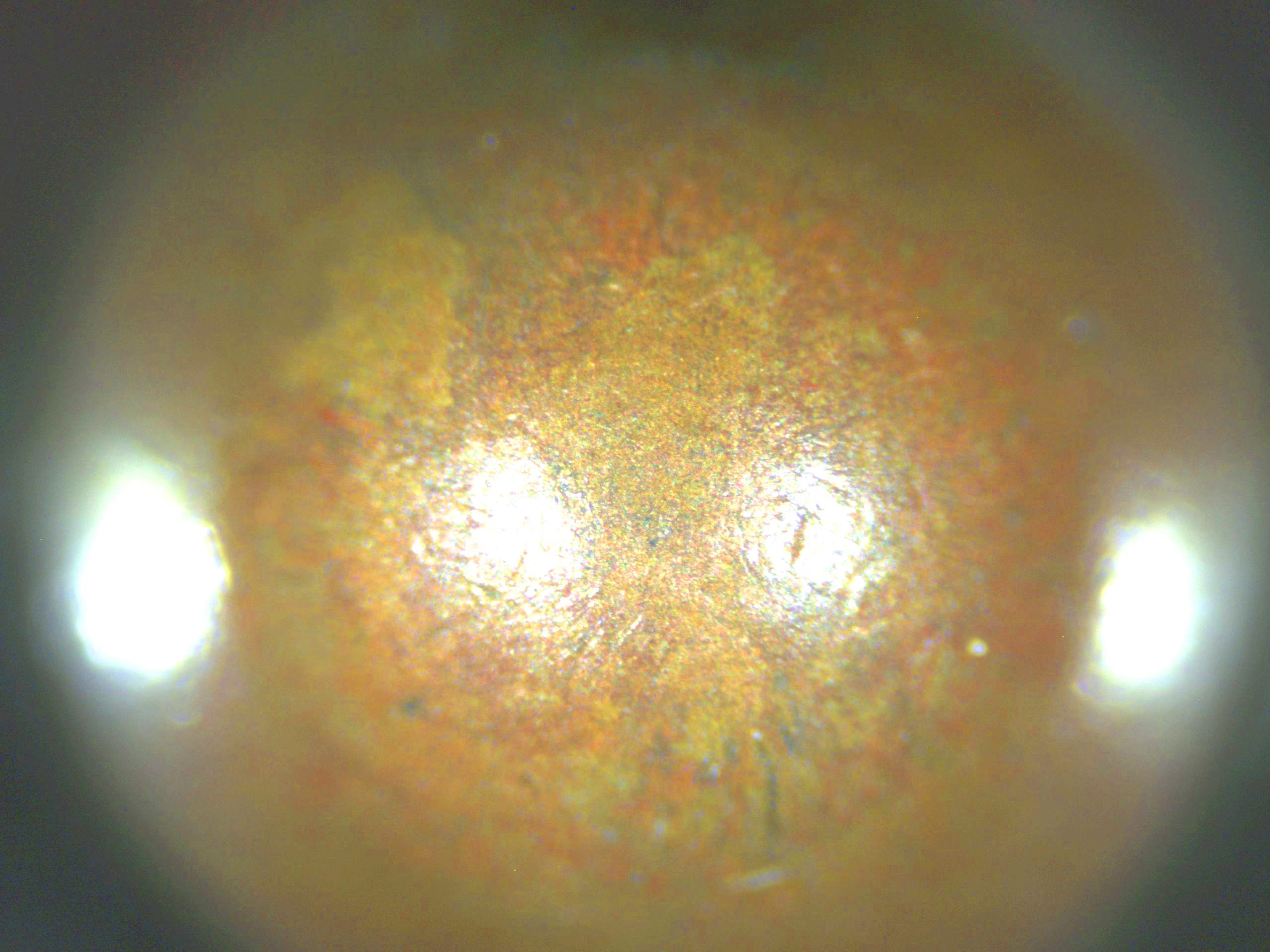

Supplement: S1 Dataset — (ZIP) [file pone.0191085.s001.zip › data set 1/FD261.jpg]

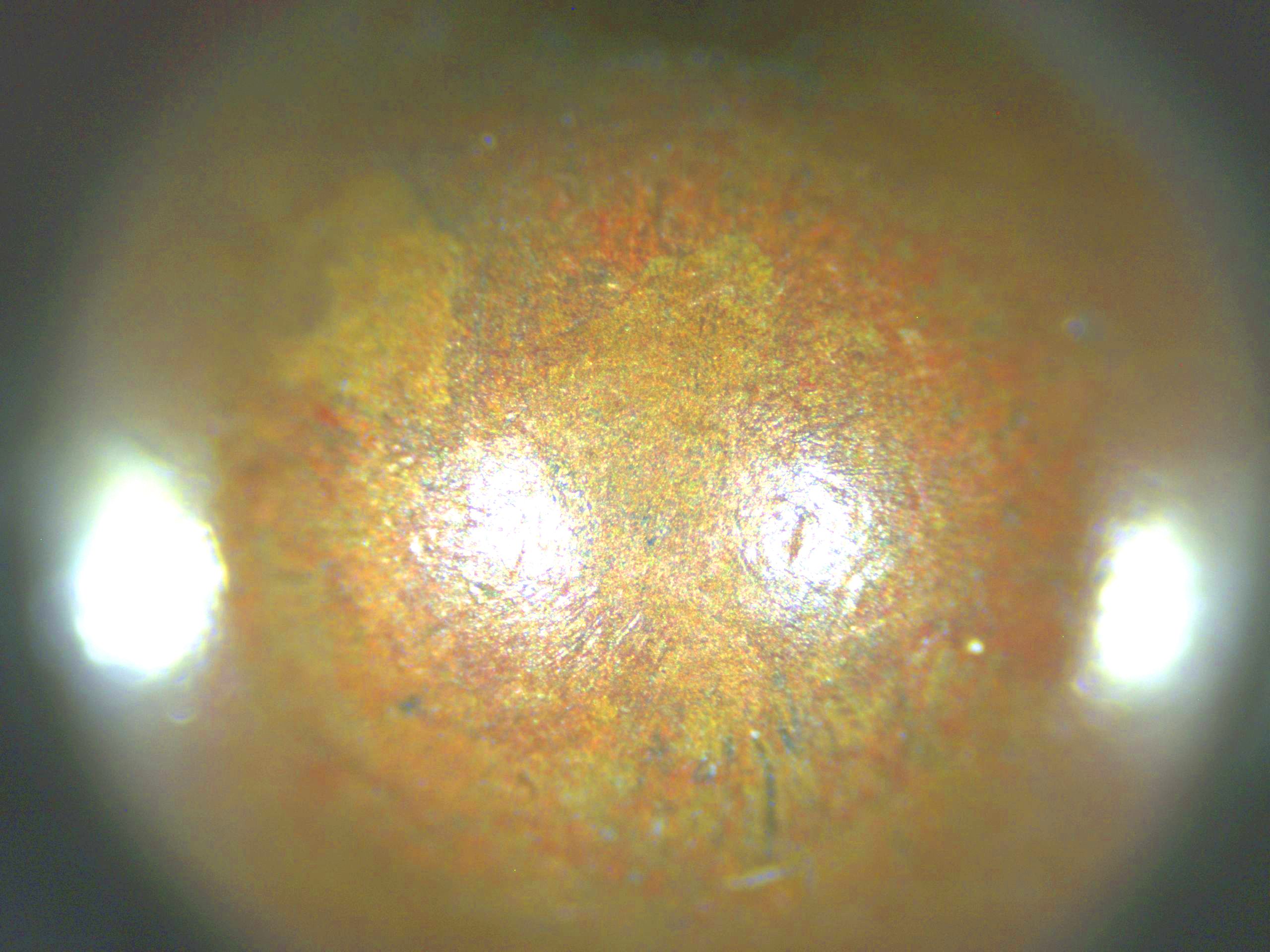

Supplement: S1 Dataset — (ZIP) [file pone.0191085.s001.zip › data set 1/FD262.jpg]

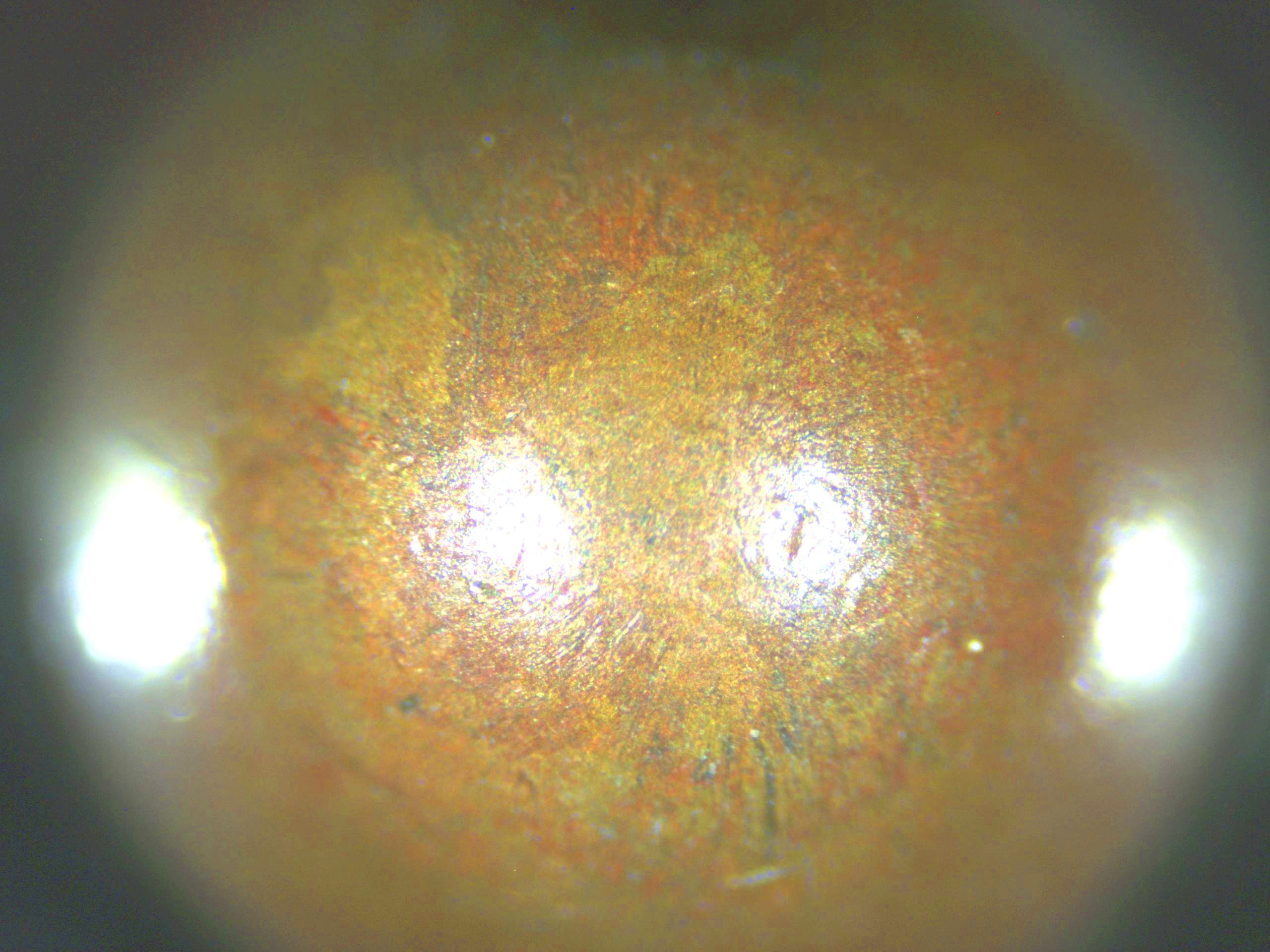

Supplement: S1 Dataset — (ZIP) [file pone.0191085.s001.zip › data set 1/FD263.jpg]

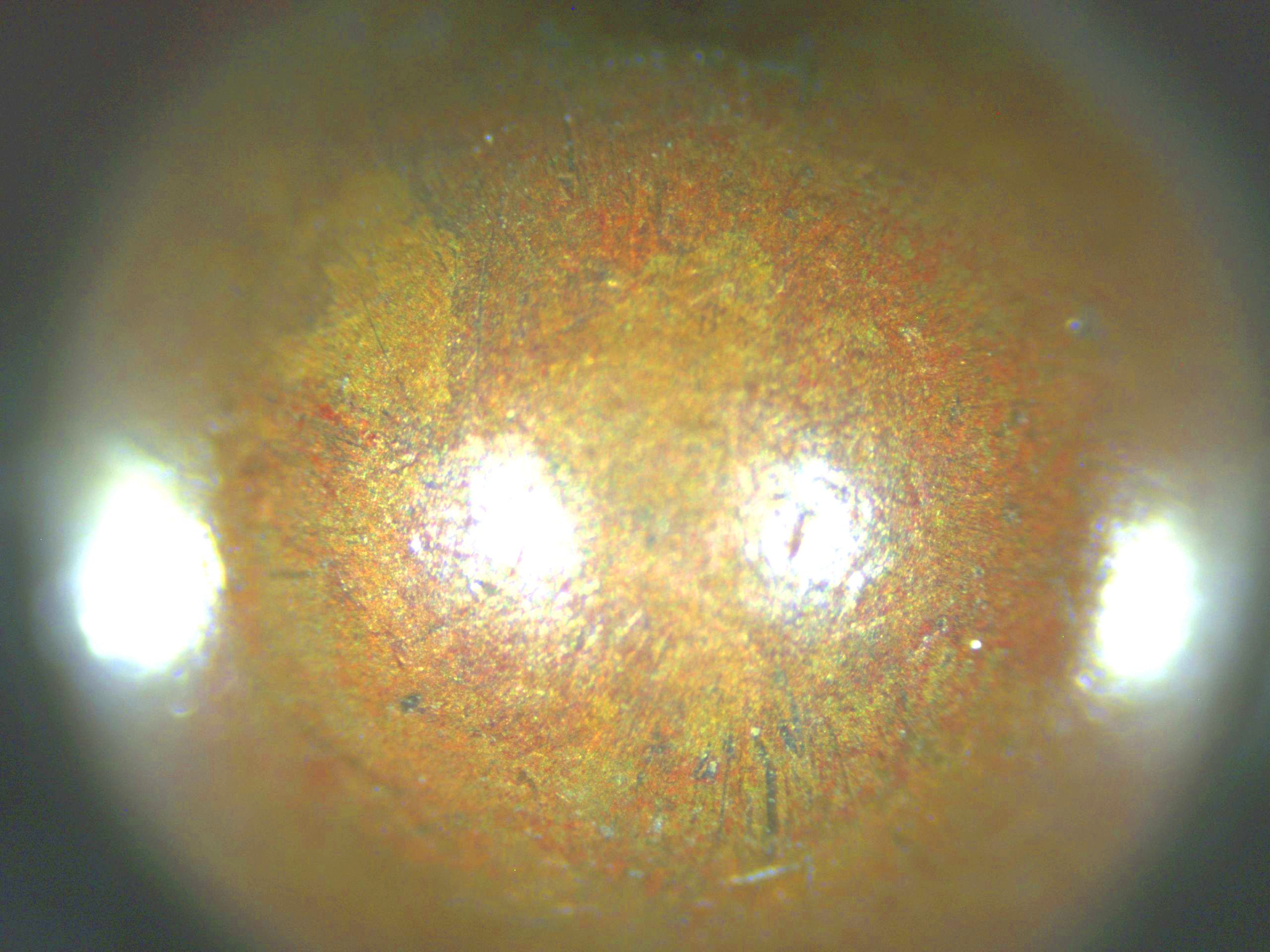

Supplement: S1 Dataset — (ZIP) [file pone.0191085.s001.zip › data set 1/FD265.jpg]

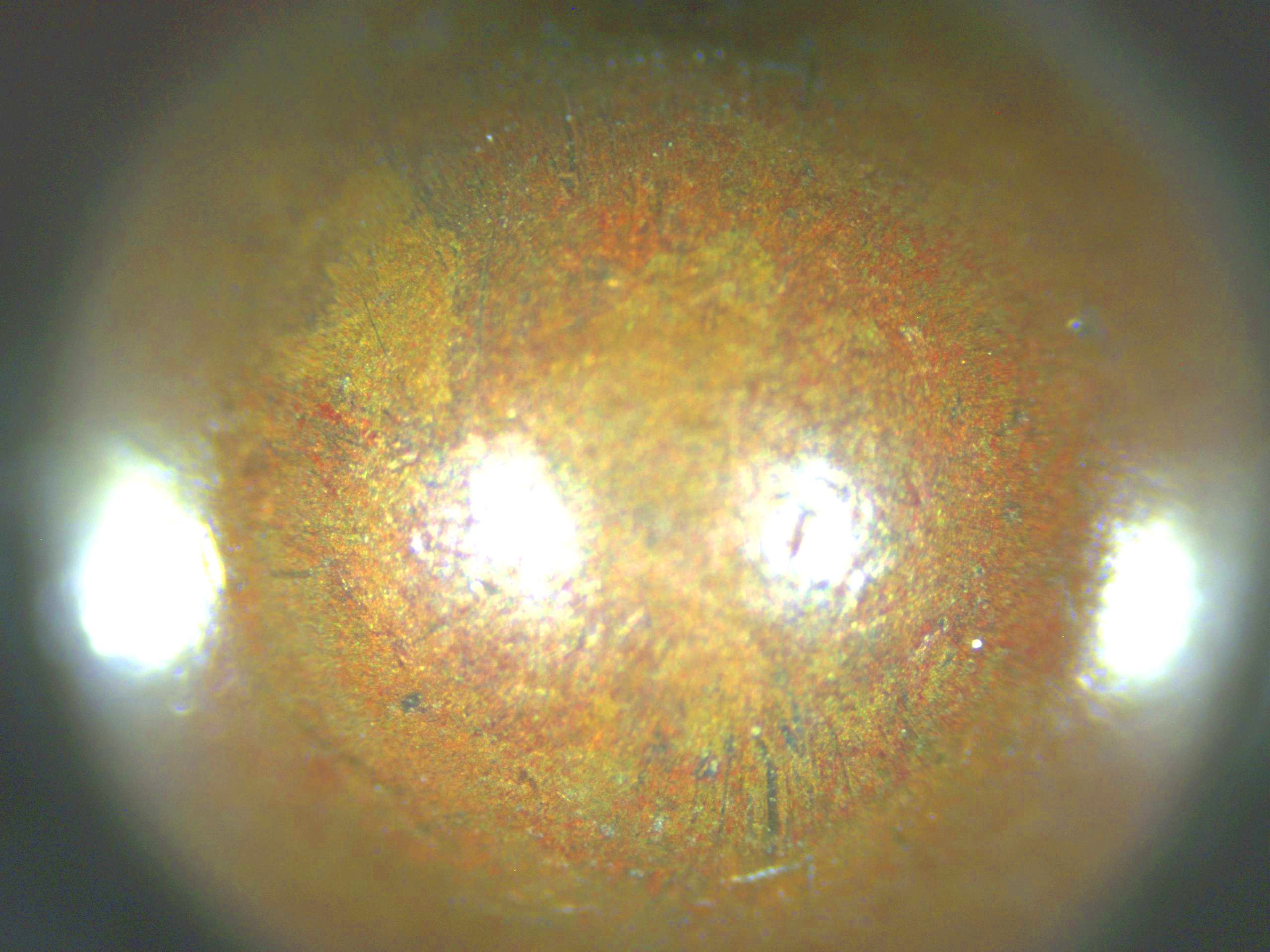

Supplement: S1 Dataset — (ZIP) [file pone.0191085.s001.zip › data set 1/FD266.jpg]

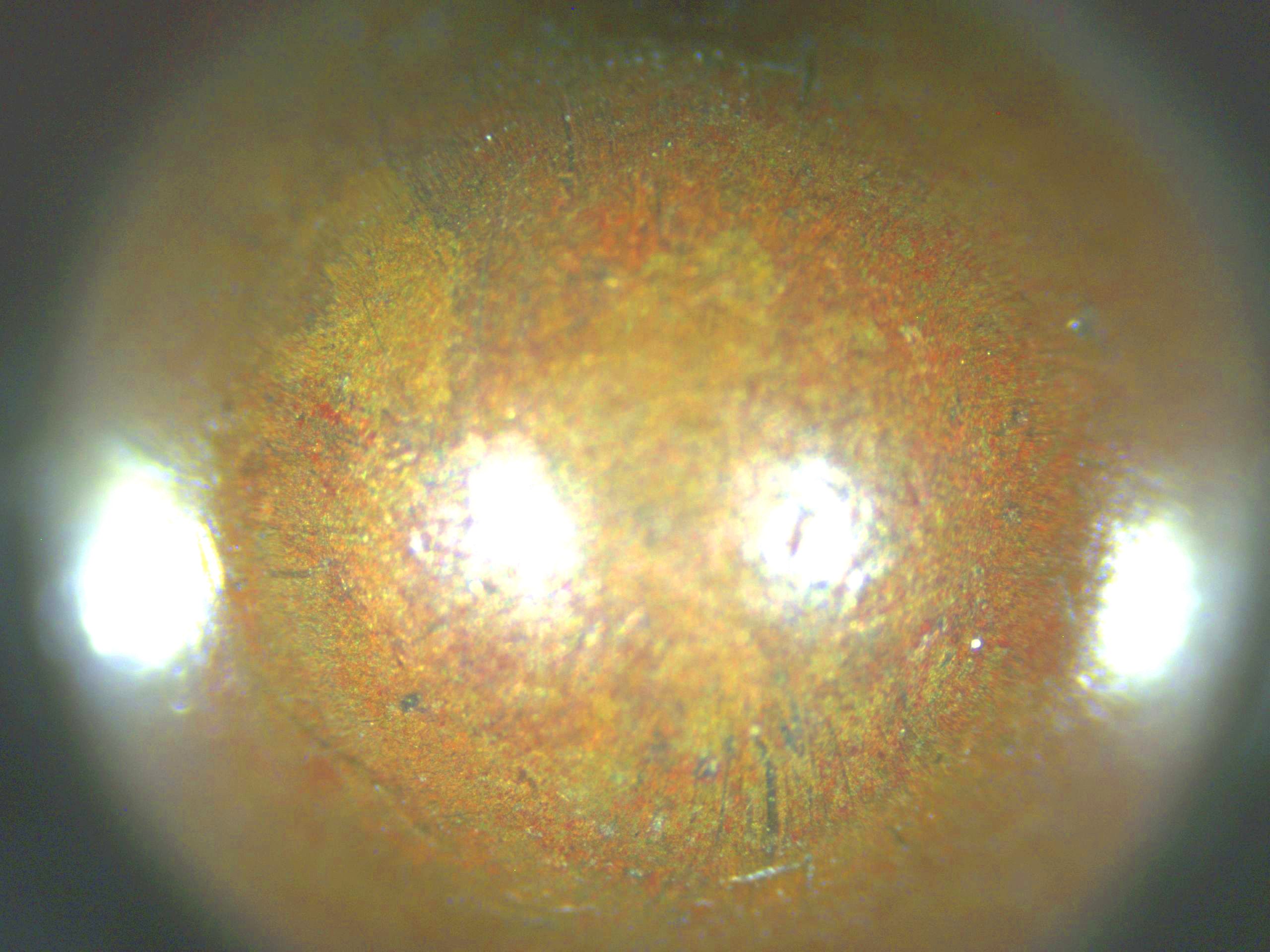

Supplement: S1 Dataset — (ZIP) [file pone.0191085.s001.zip › data set 1/FD267.jpg]

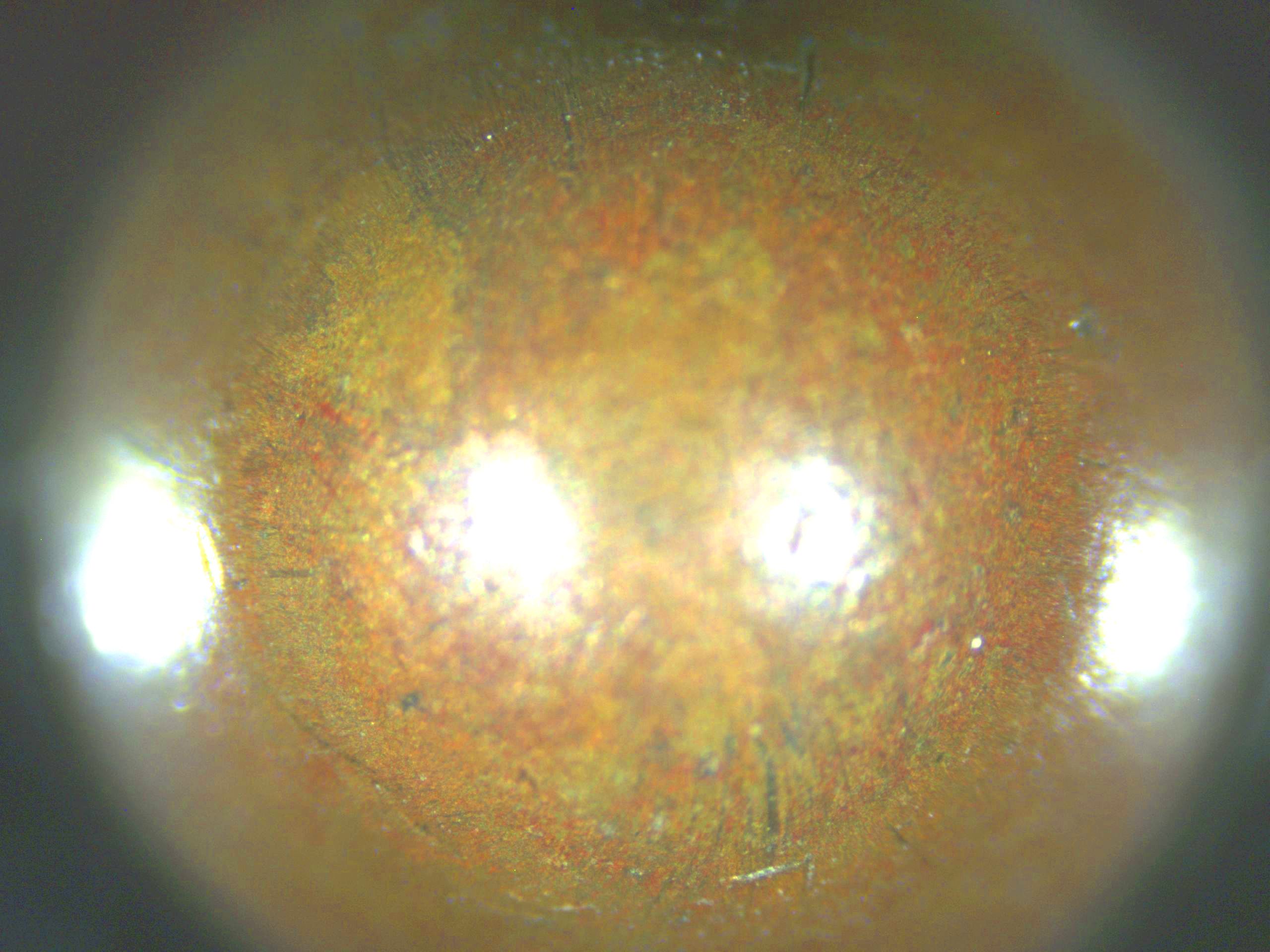

Supplement: S1 Dataset — (ZIP) [file pone.0191085.s001.zip › data set 1/FD268.jpg]

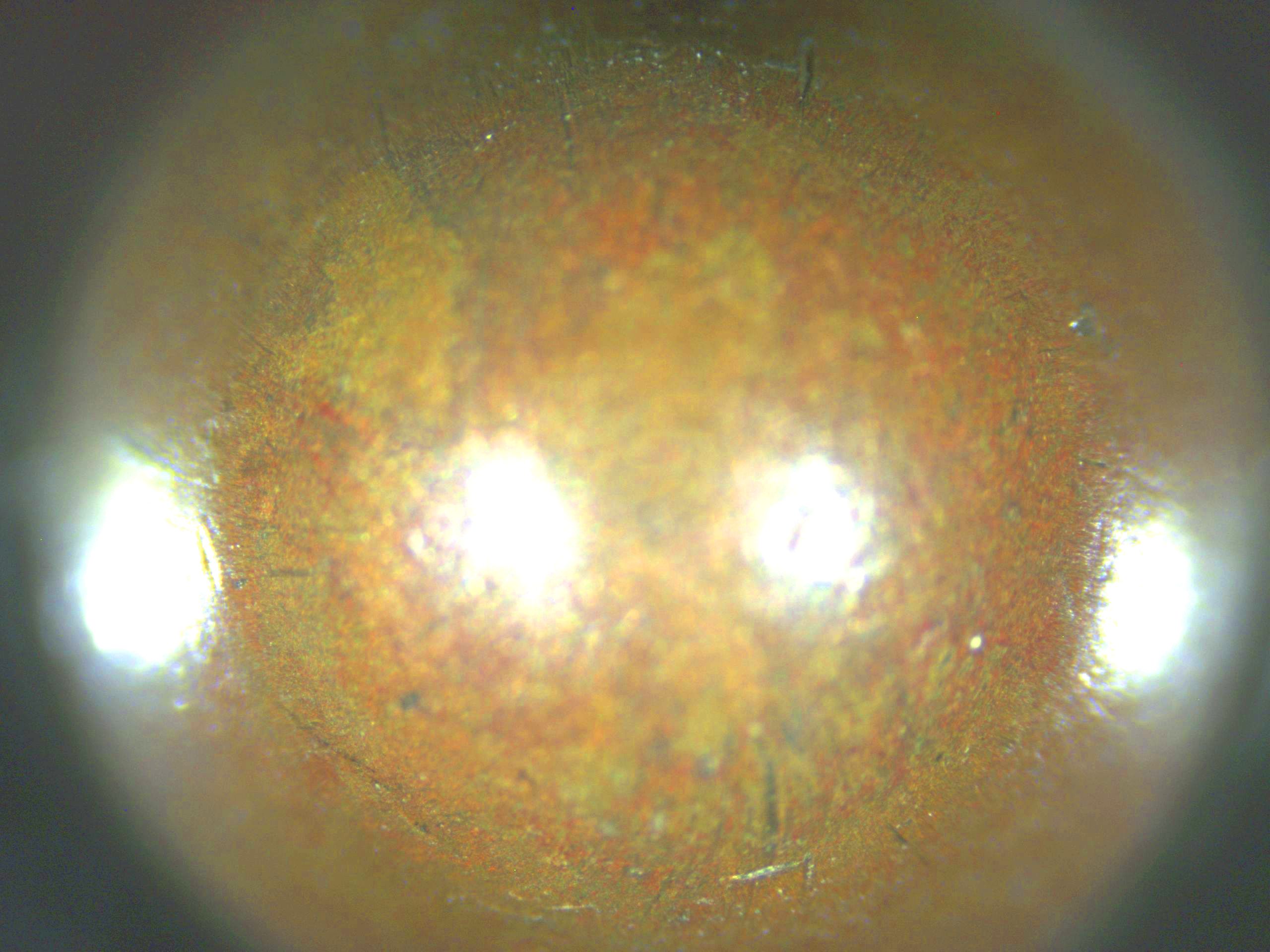

Supplement: S1 Dataset — (ZIP) [file pone.0191085.s001.zip › data set 1/FD269.jpg]

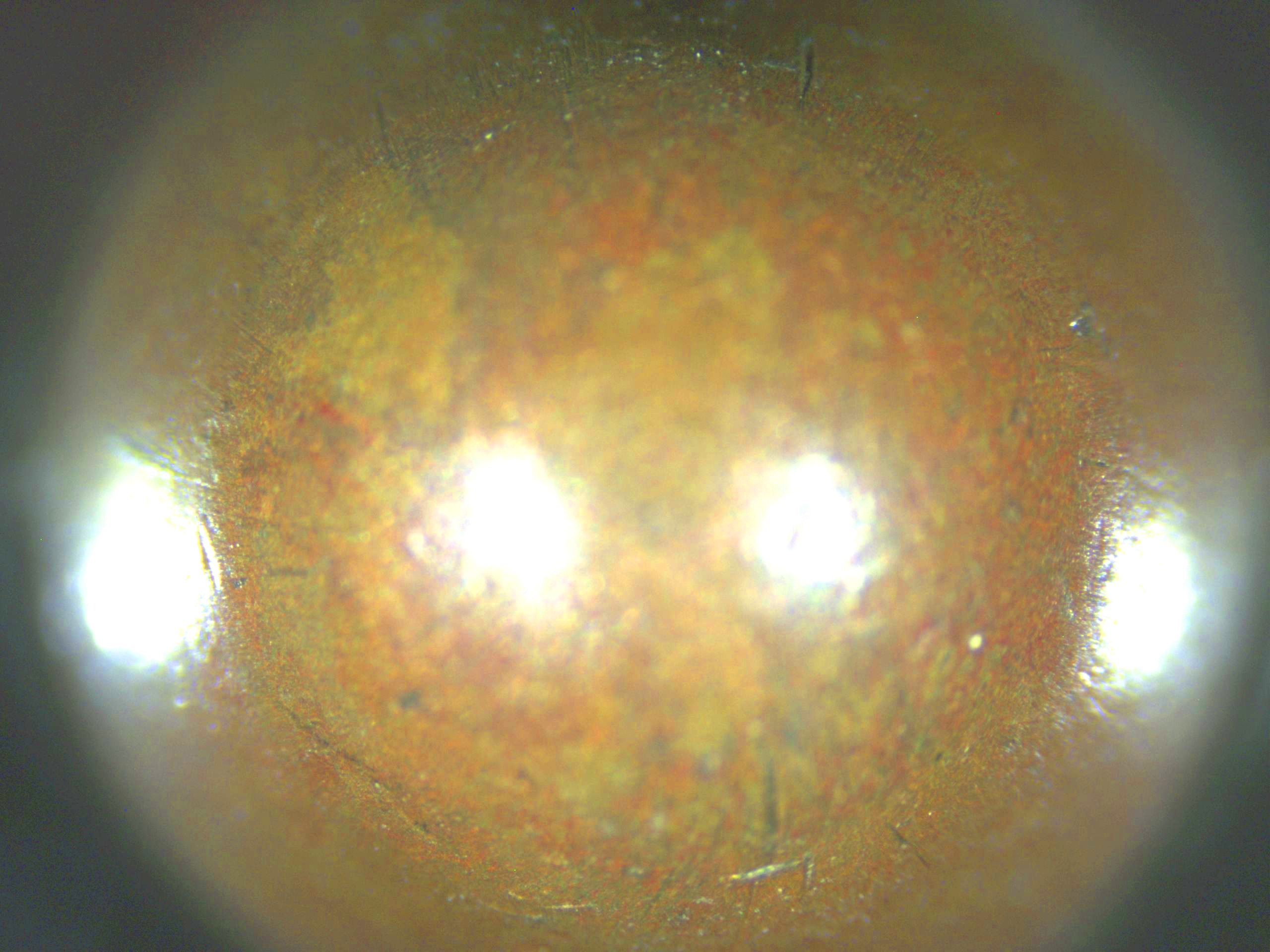

Supplement: S1 Dataset — (ZIP) [file pone.0191085.s001.zip › data set 1/FD270.jpg]

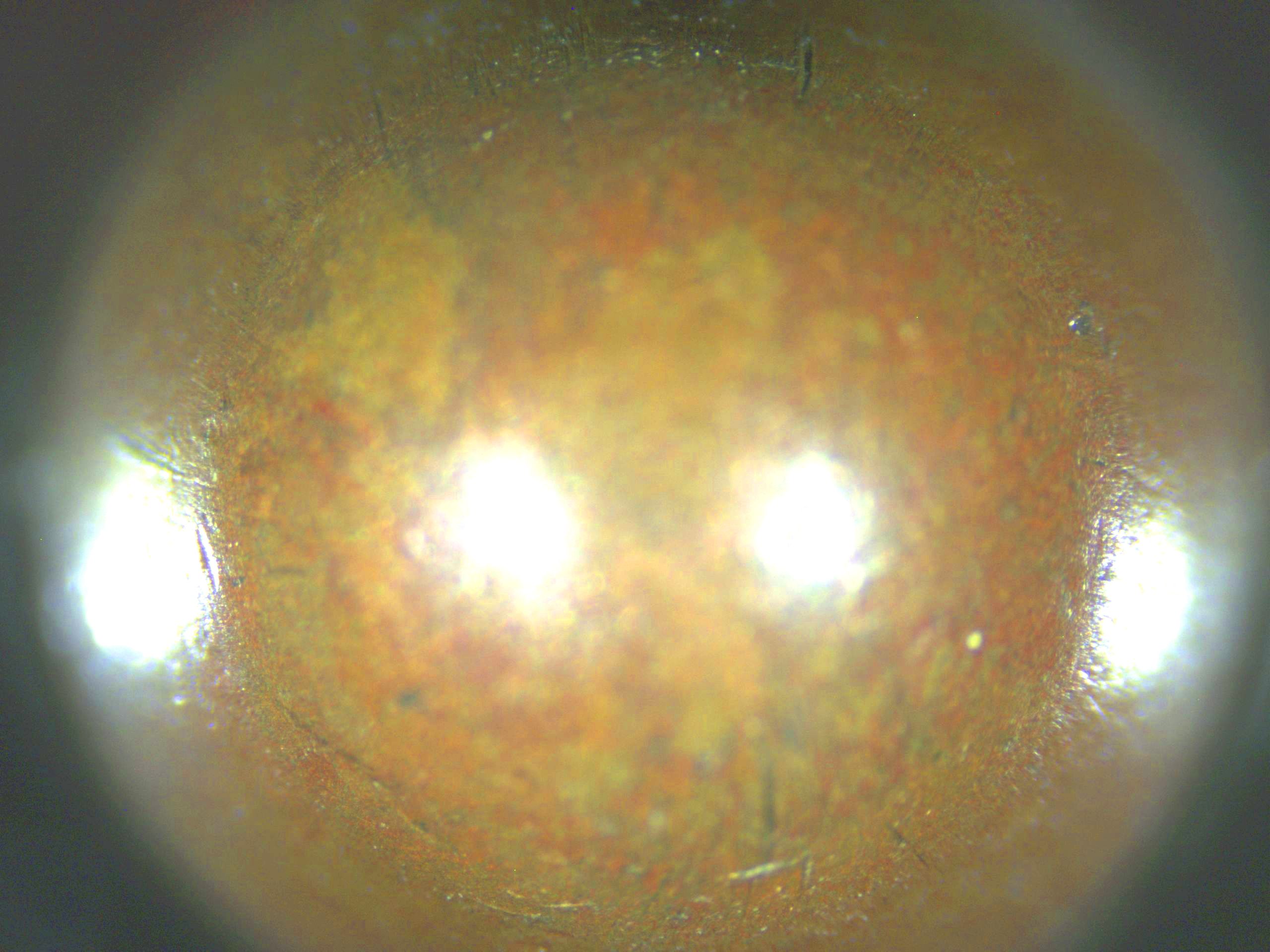

Supplement: S1 Dataset — (ZIP) [file pone.0191085.s001.zip › data set 1/FD271.jpg]

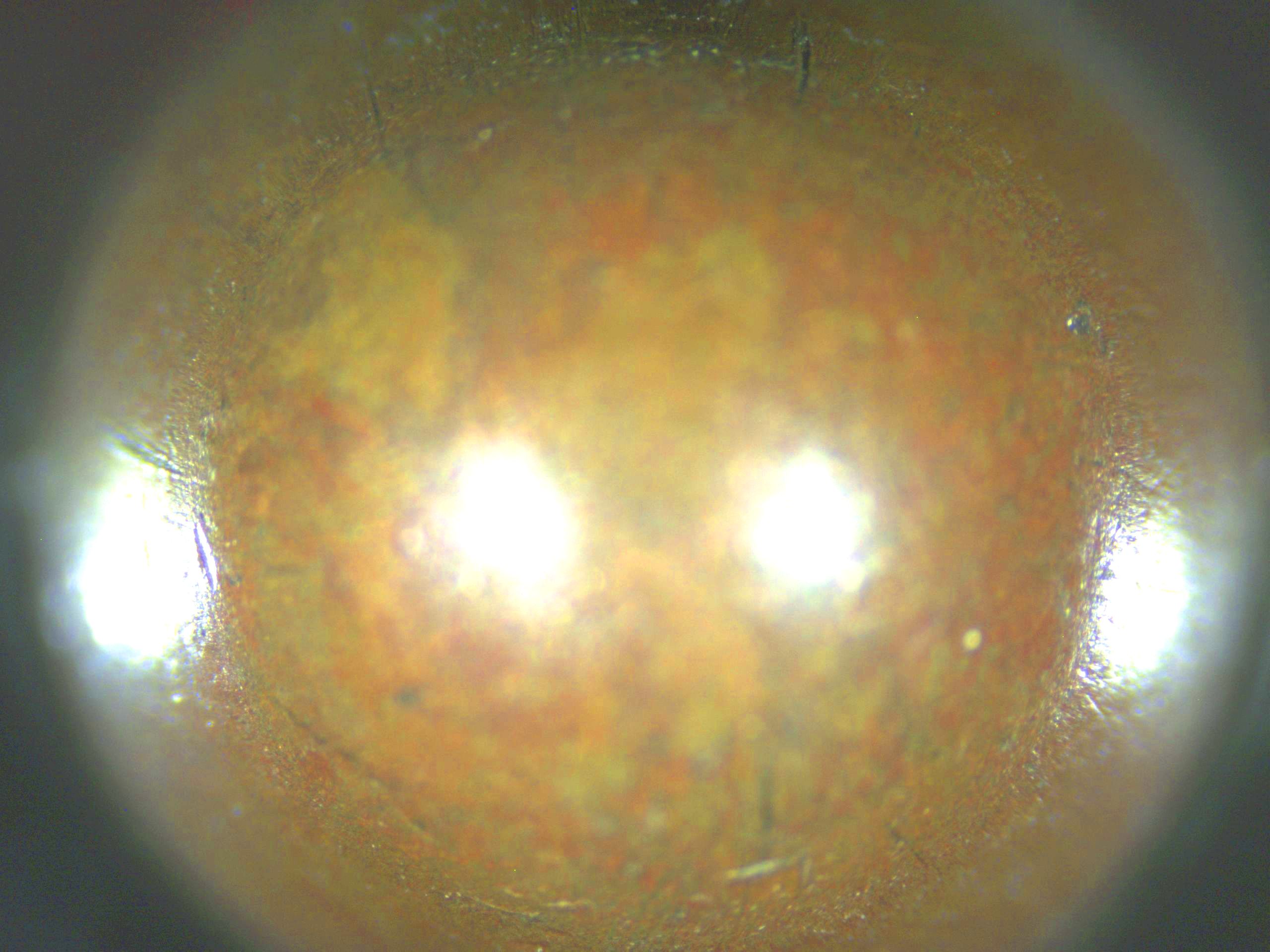

Supplement: S1 Dataset — (ZIP) [file pone.0191085.s001.zip › data set 1/FD272.jpg]

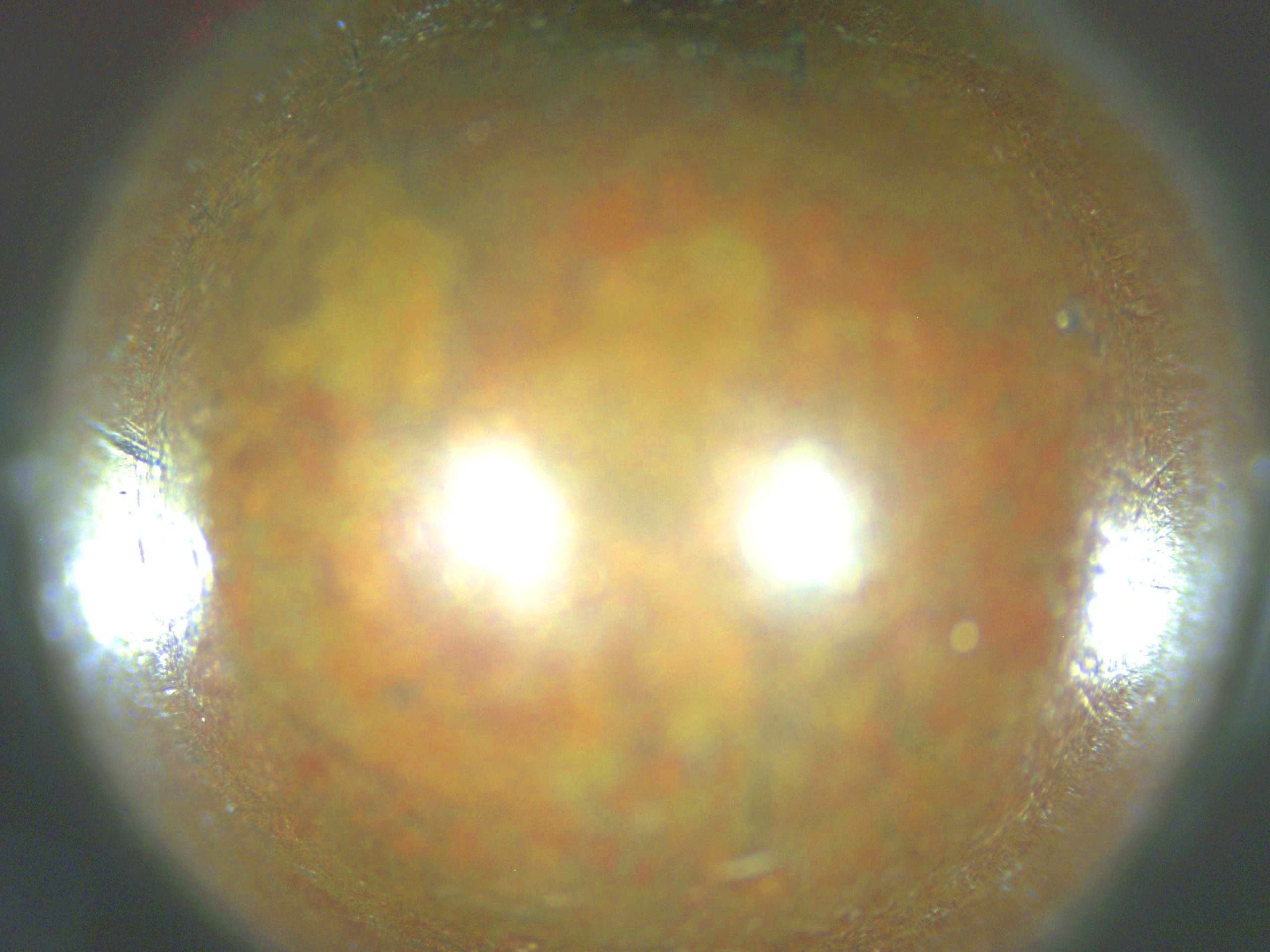

Supplement: S1 Dataset — (ZIP) [file pone.0191085.s001.zip › data set 1/FD274.jpg]

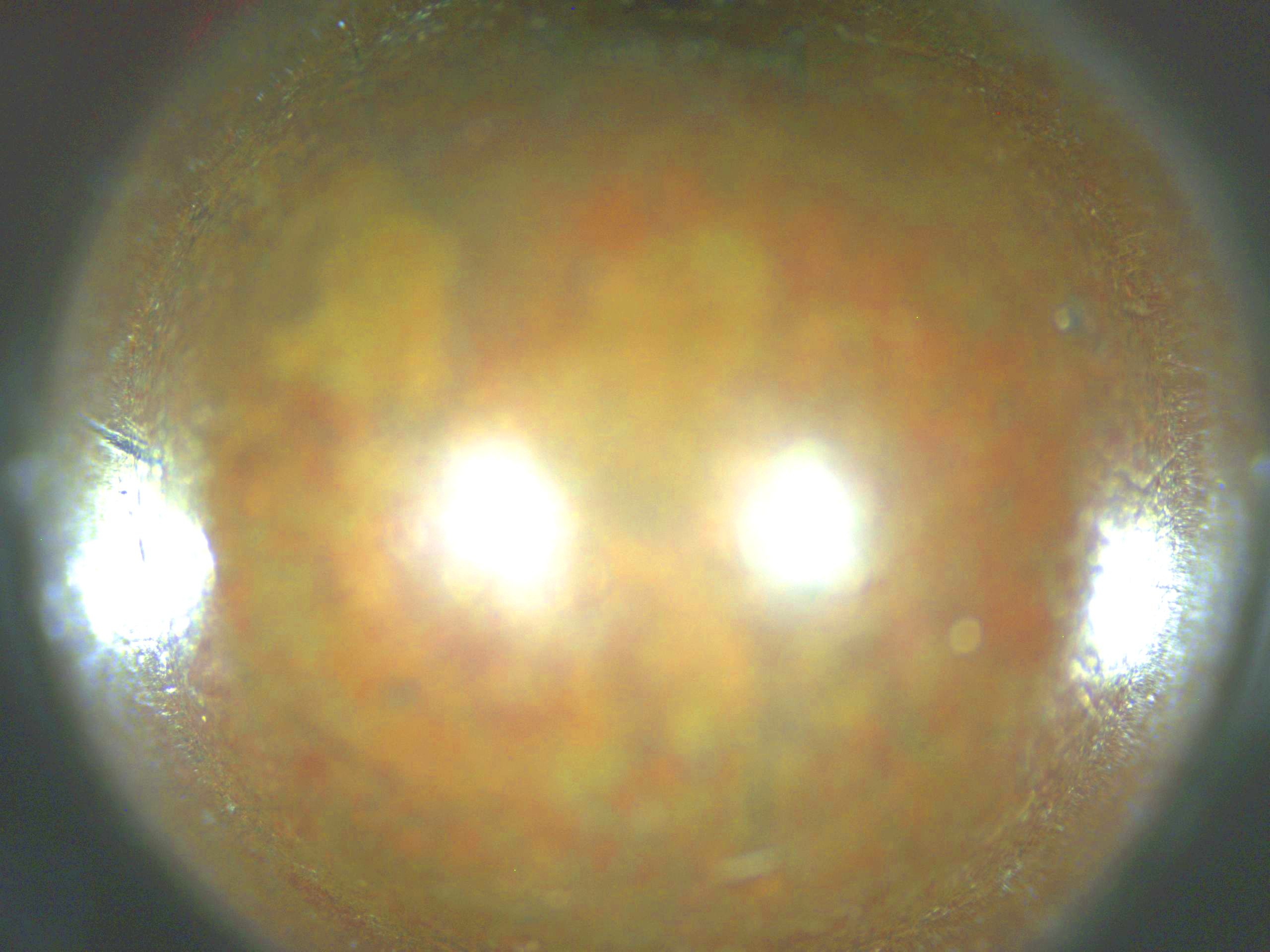

Supplement: S1 Dataset — (ZIP) [file pone.0191085.s001.zip › data set 1/FD275.jpg]

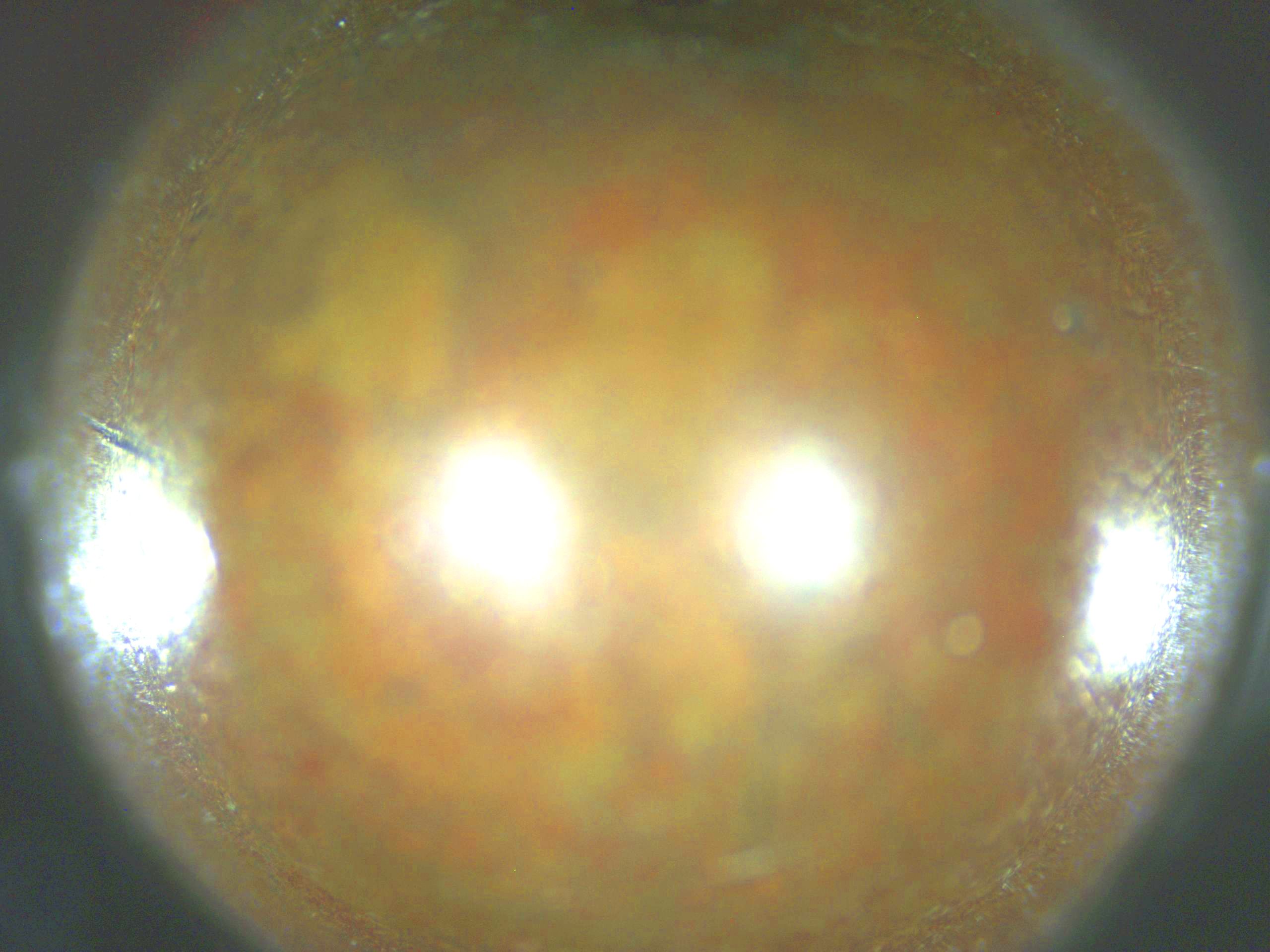

Supplement: S1 Dataset — (ZIP) [file pone.0191085.s001.zip › data set 1/FD276.jpg]

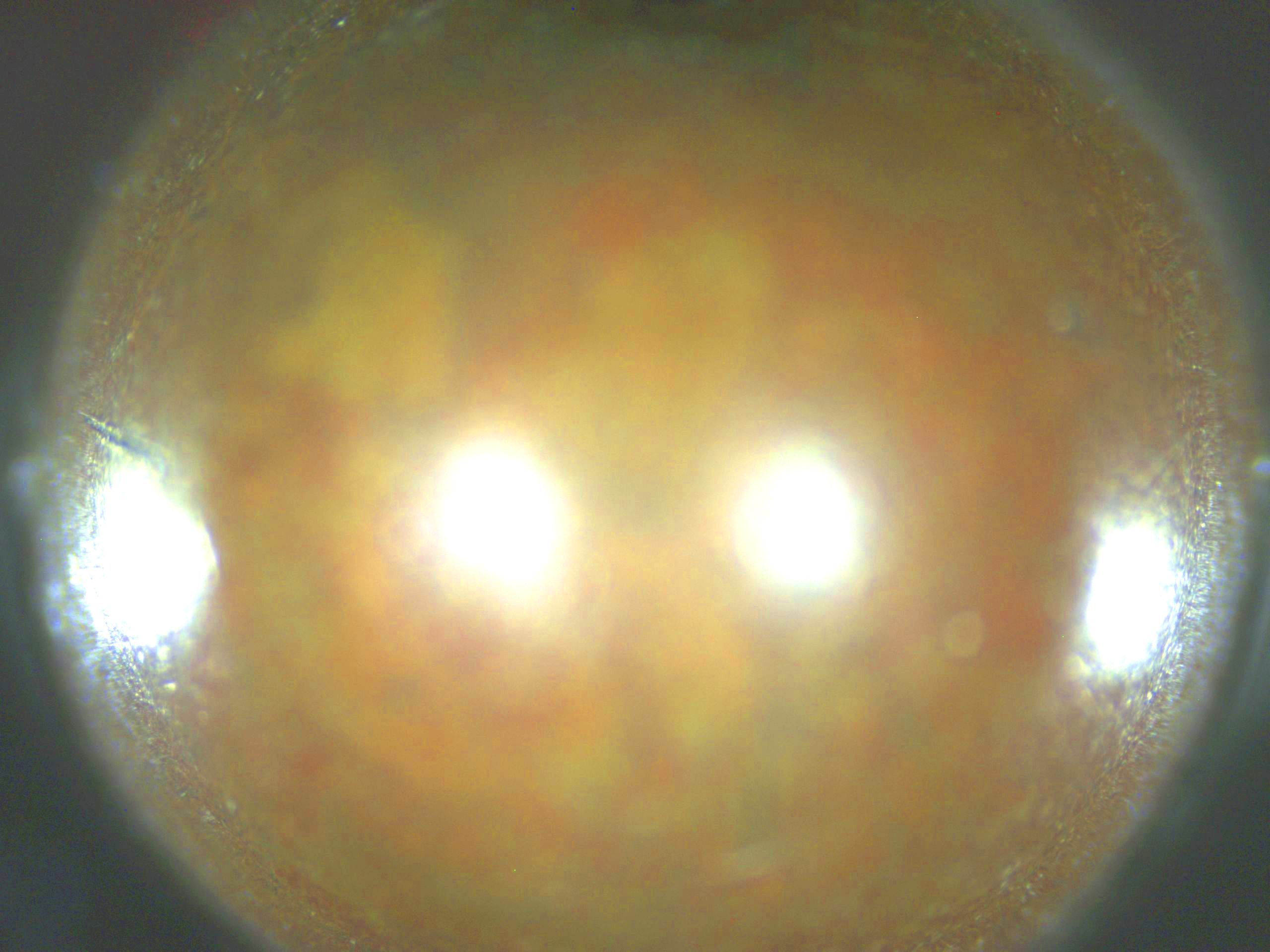

Supplement: S1 Dataset — (ZIP) [file pone.0191085.s001.zip › data set 1/FD277.jpg]

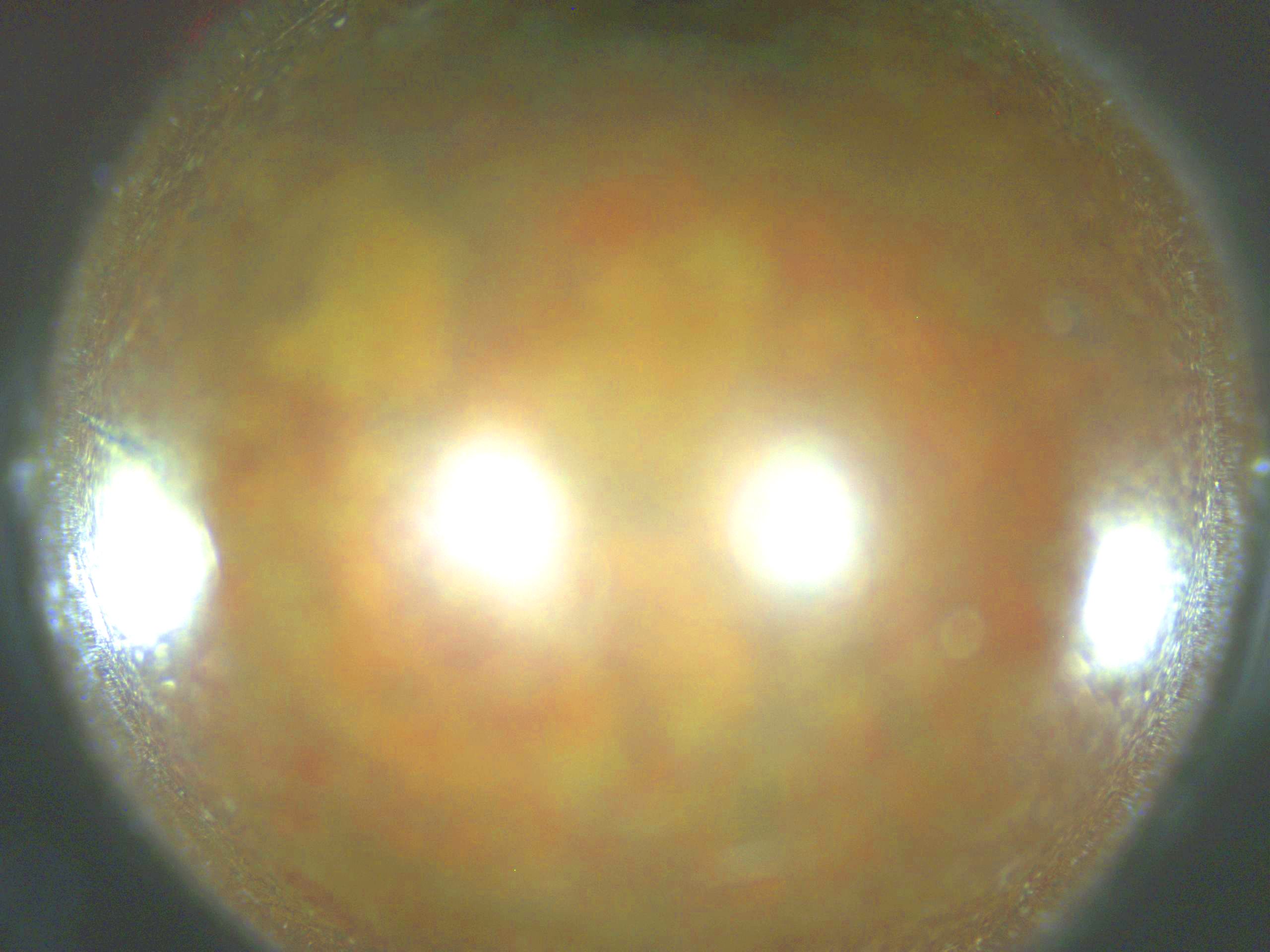

Supplement: S1 Dataset — (ZIP) [file pone.0191085.s001.zip › data set 1/FD278.jpg]

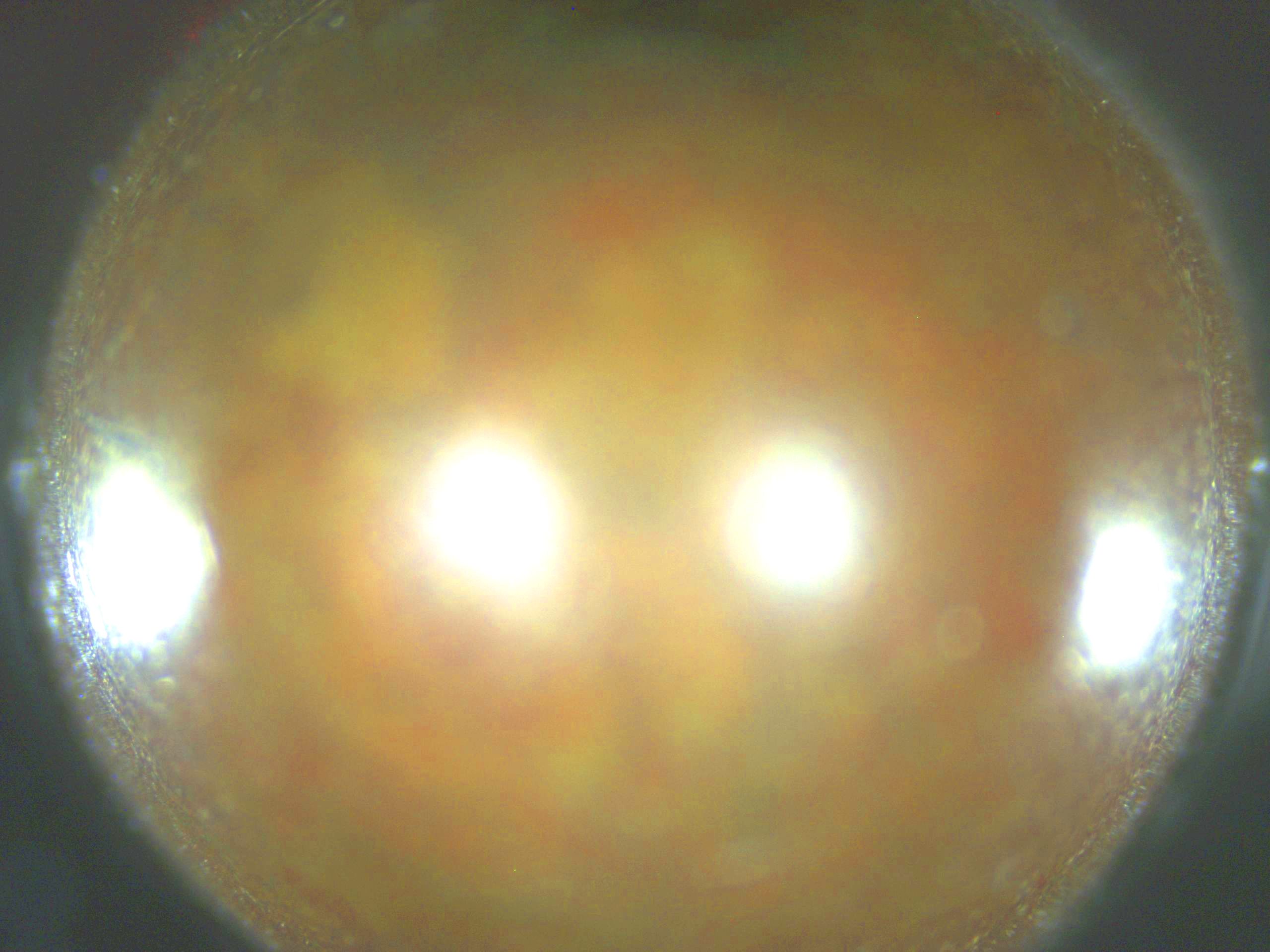

Supplement: S1 Dataset — (ZIP) [file pone.0191085.s001.zip › data set 1/FD279.jpg]

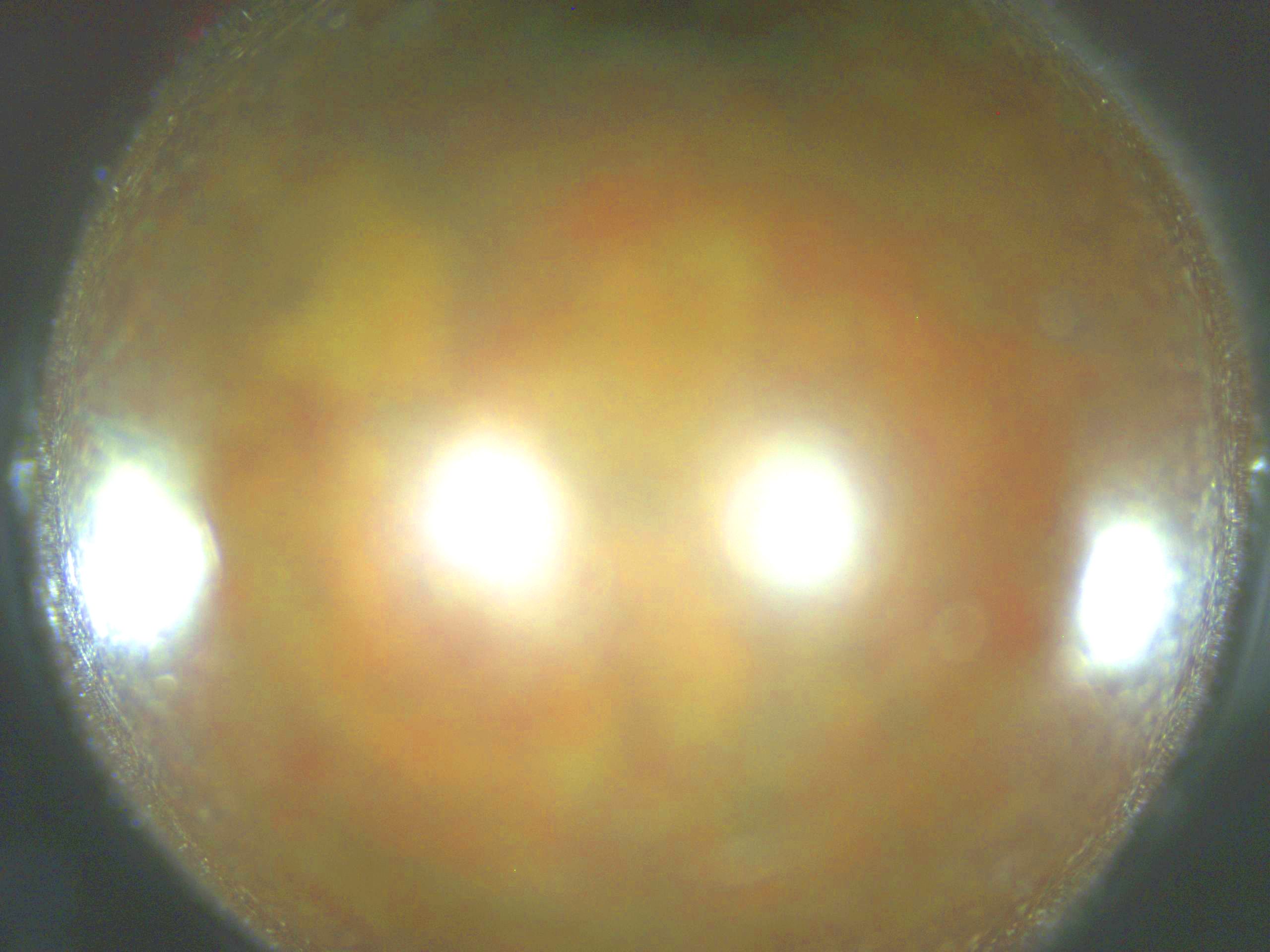

Supplement: S1 Dataset — (ZIP) [file pone.0191085.s001.zip › data set 1/FD280.jpg]

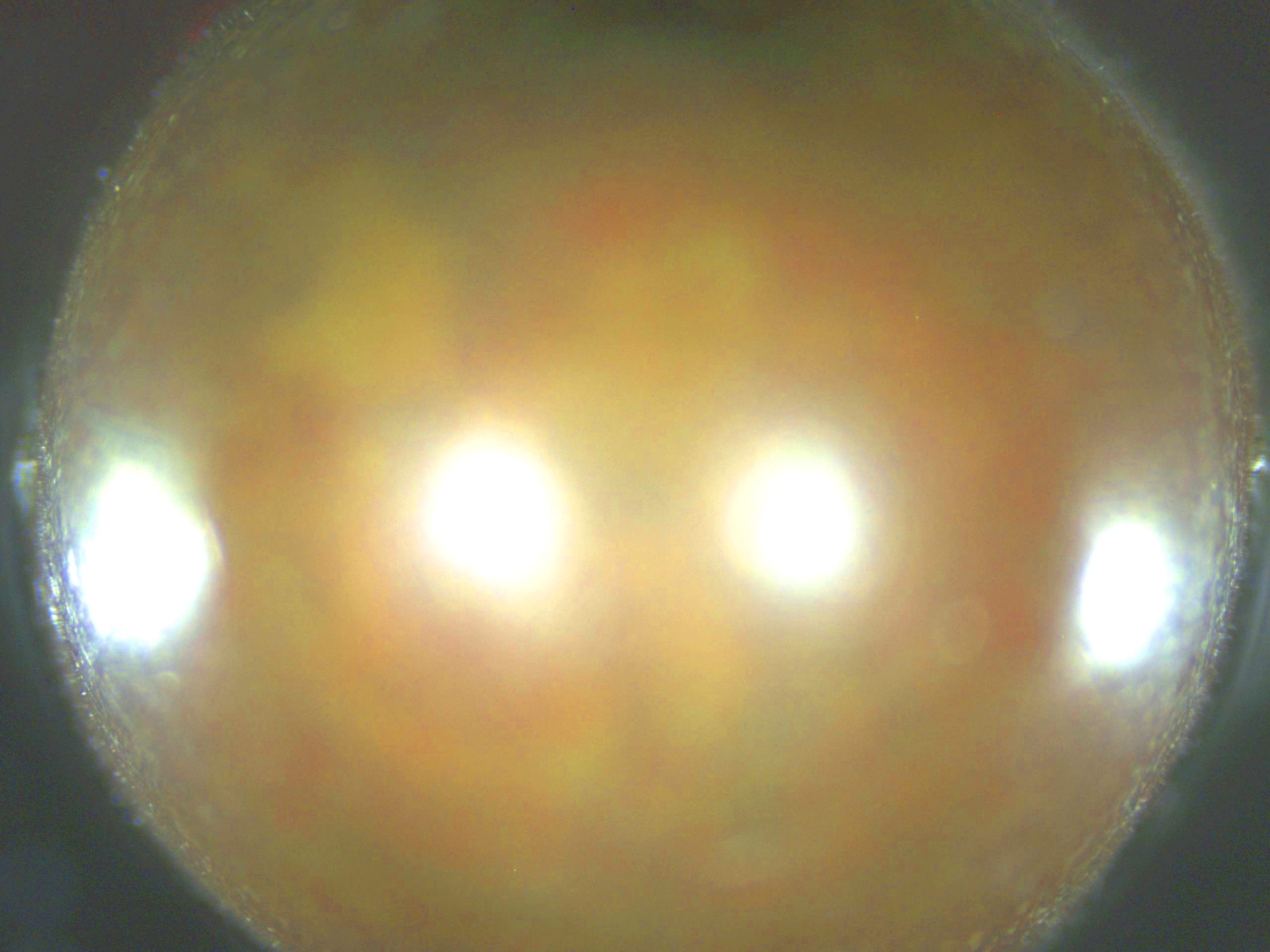

Supplement: S1 Dataset — (ZIP) [file pone.0191085.s001.zip › data set 1/FD281.jpg]

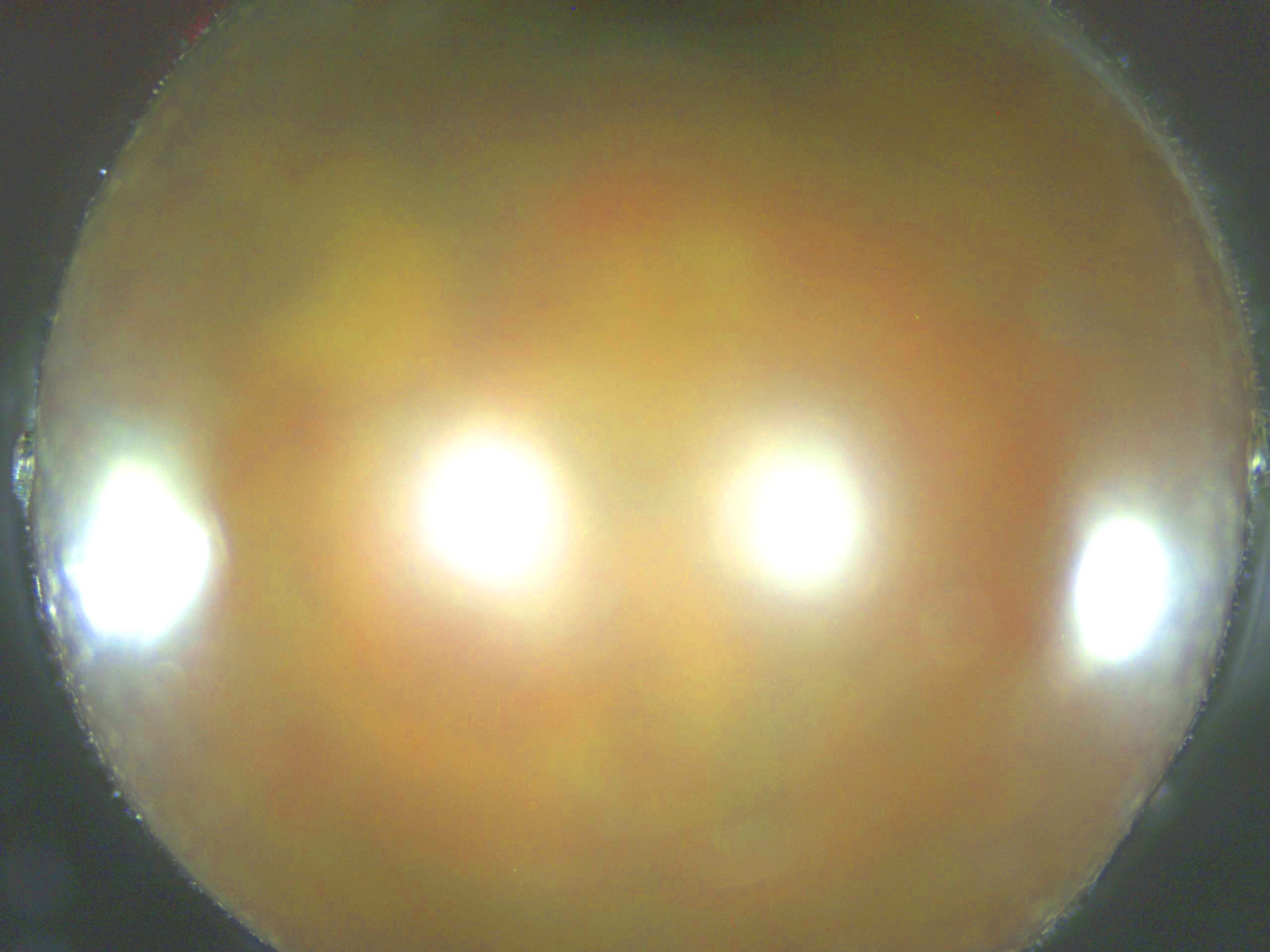

Supplement: S1 Dataset — (ZIP) [file pone.0191085.s001.zip › data set 1/FD283.jpg]

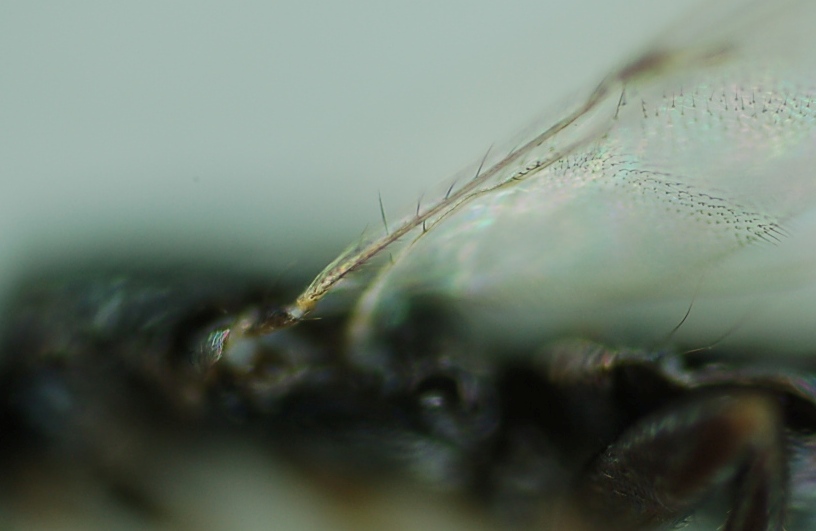

Supplement: S2 Dataset — (ZIP) [file pone.0191085.s002.zip › data set 2/Img10.jpg]

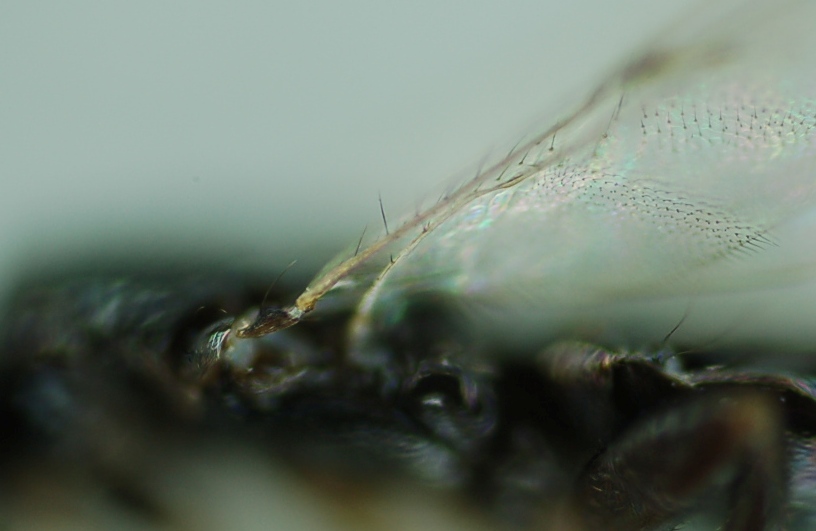

Supplement: S2 Dataset — (ZIP) [file pone.0191085.s002.zip › data set 2/Img11.jpg]

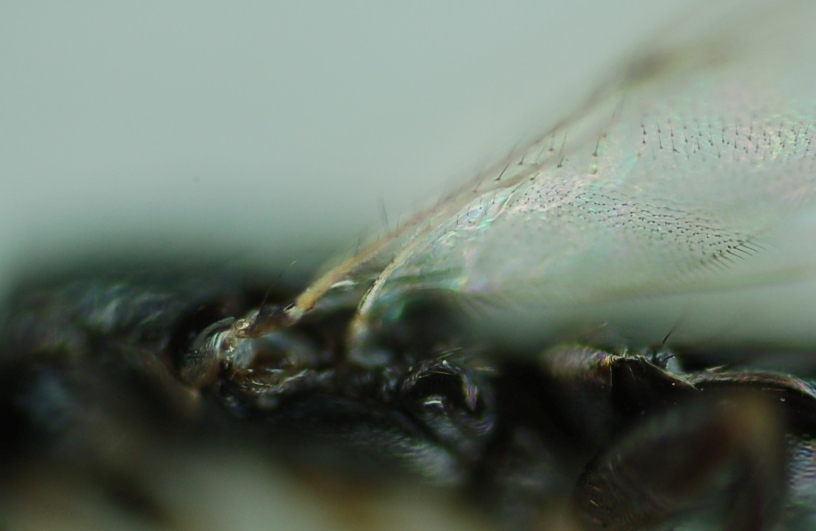

Supplement: S2 Dataset — (ZIP) [file pone.0191085.s002.zip › data set 2/Img12.jpg]

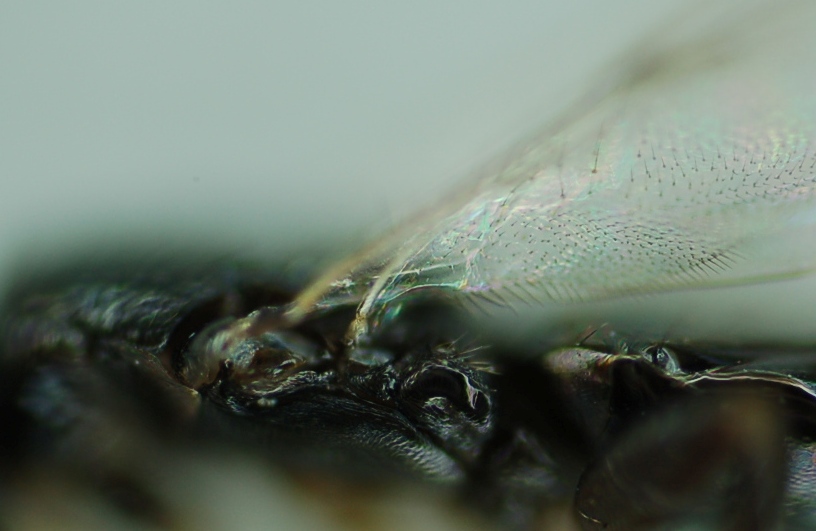

Supplement: S2 Dataset — (ZIP) [file pone.0191085.s002.zip › data set 2/Img13.jpg]

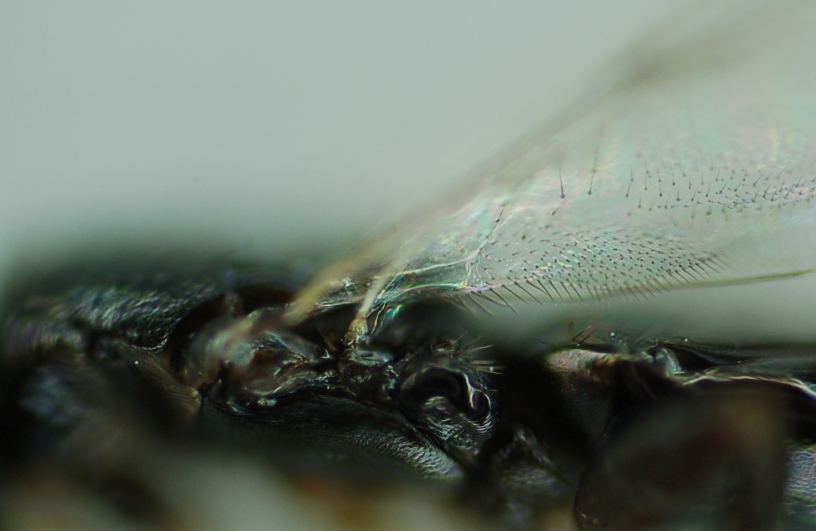

Supplement: S2 Dataset — (ZIP) [file pone.0191085.s002.zip › data set 2/Img14.jpg]

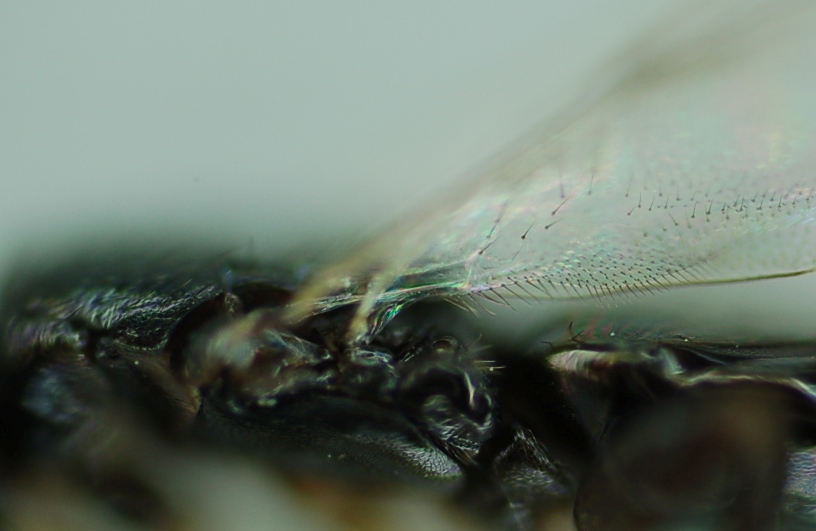

Supplement: S2 Dataset — (ZIP) [file pone.0191085.s002.zip › data set 2/Img15.jpg]

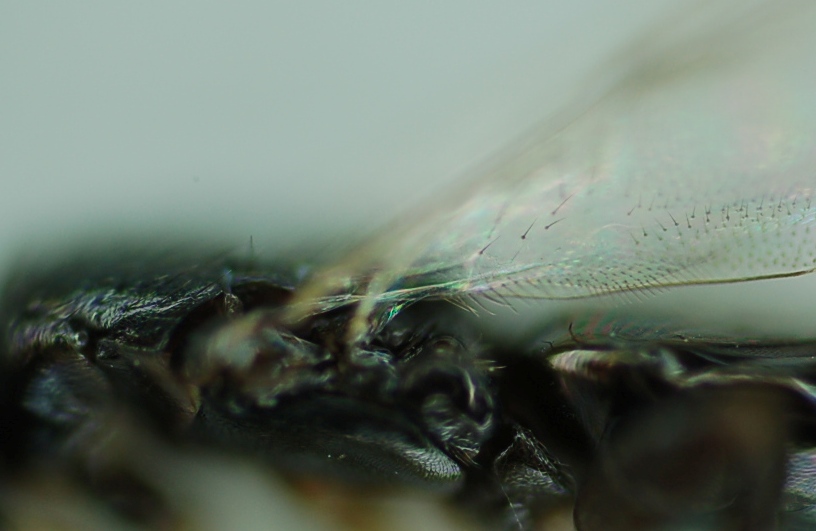

Supplement: S2 Dataset — (ZIP) [file pone.0191085.s002.zip › data set 2/Img16.jpg]

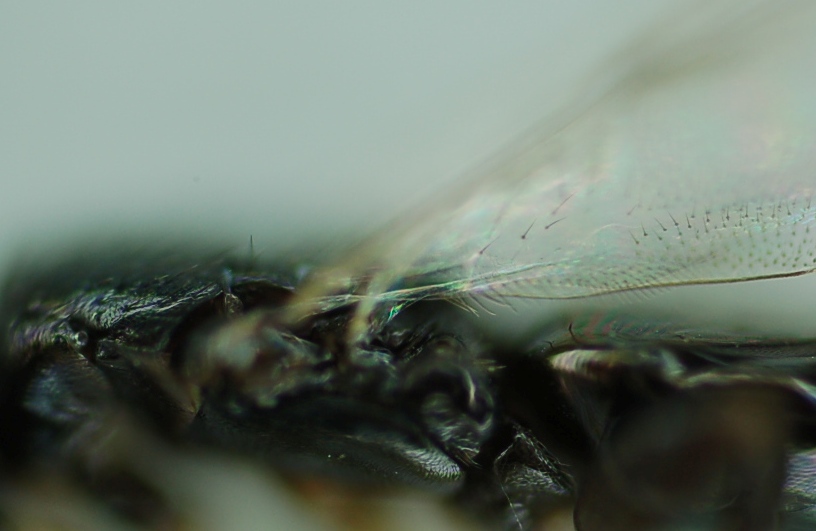

Supplement: S2 Dataset — (ZIP) [file pone.0191085.s002.zip › data set 2/Img17.jpg]

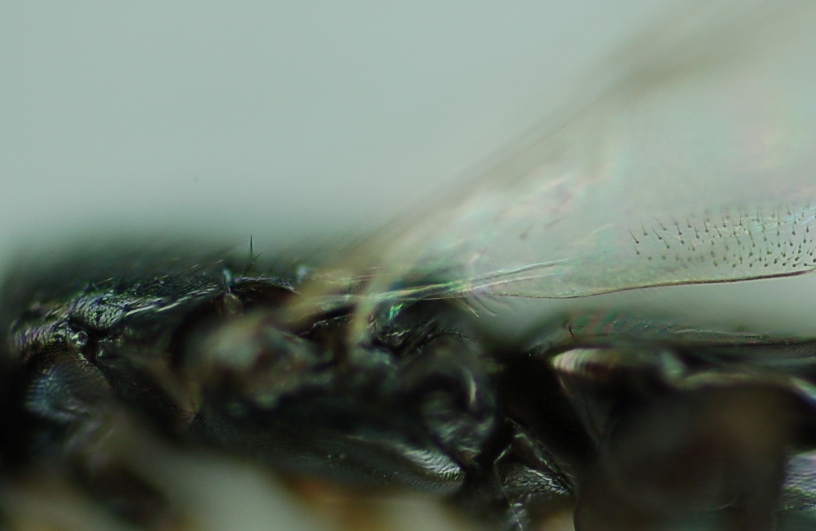

Supplement: S2 Dataset — (ZIP) [file pone.0191085.s002.zip › data set 2/Img18.jpg]

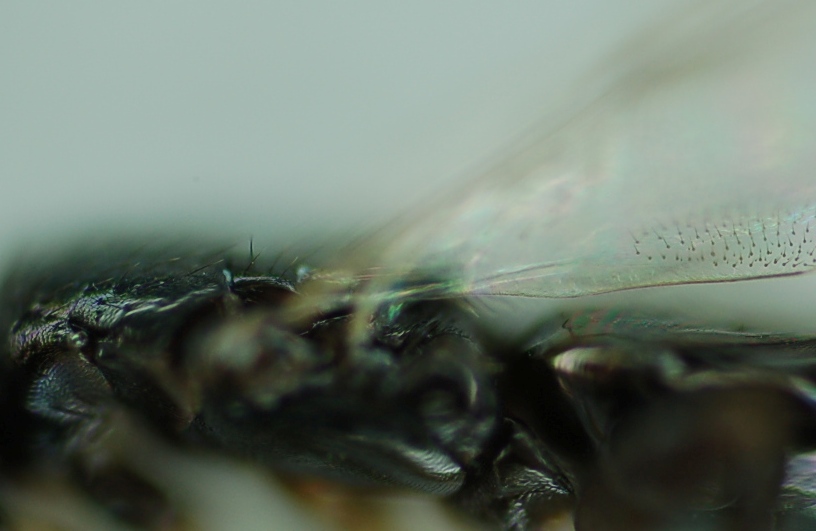

Supplement: S2 Dataset — (ZIP) [file pone.0191085.s002.zip › data set 2/Img19.jpg]

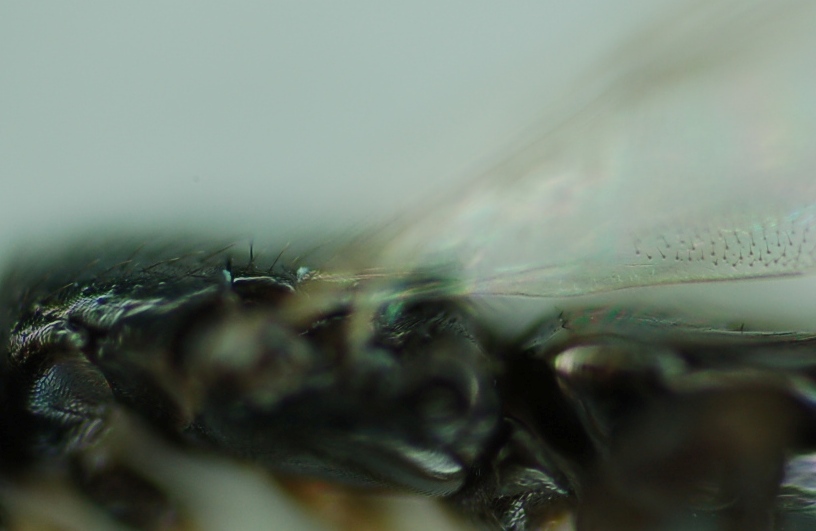

Supplement: S2 Dataset — (ZIP) [file pone.0191085.s002.zip › data set 2/Img20.jpg]

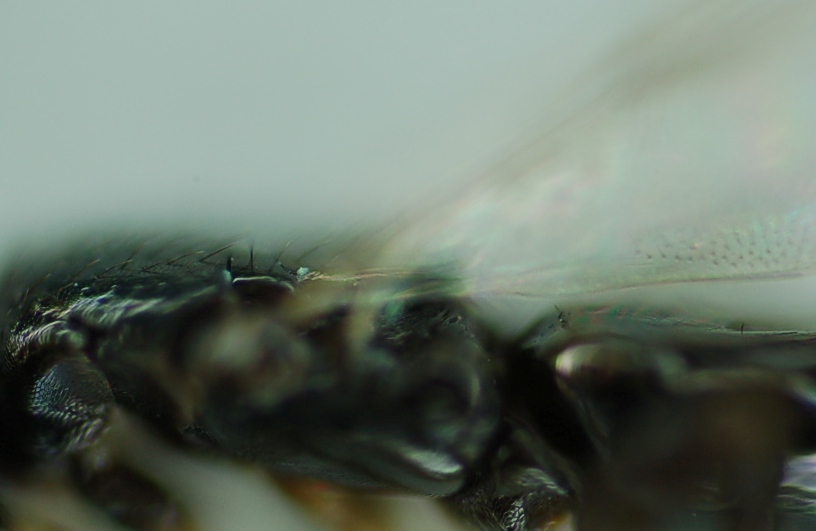

Supplement: S2 Dataset — (ZIP) [file pone.0191085.s002.zip › data set 2/Img21.jpg]

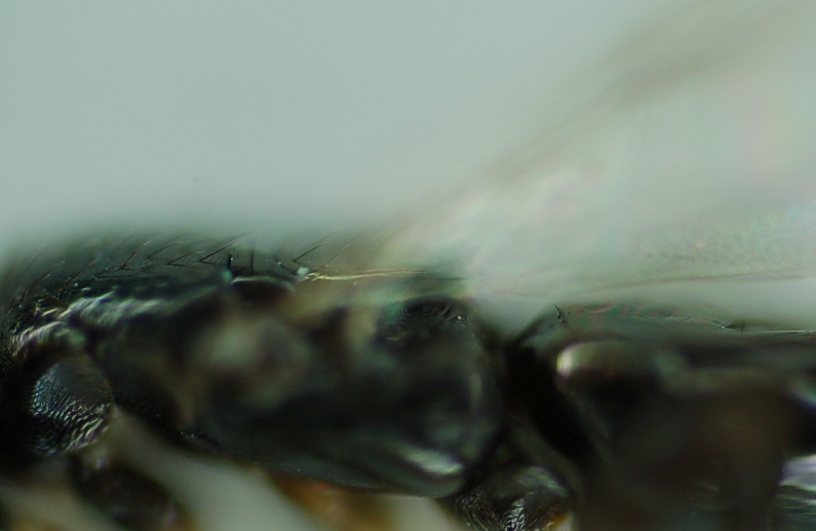

Supplement: S2 Dataset — (ZIP) [file pone.0191085.s002.zip › data set 2/Img22.jpg]

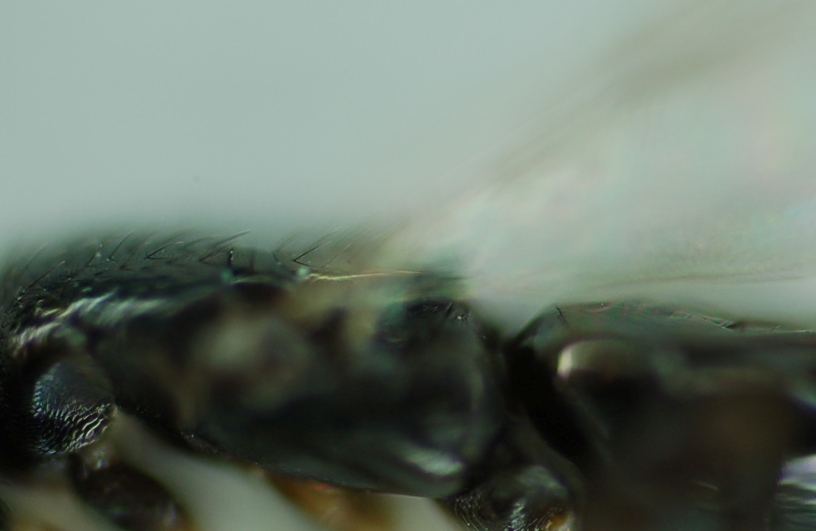

Supplement: S2 Dataset — (ZIP) [file pone.0191085.s002.zip › data set 2/Img23.jpg]

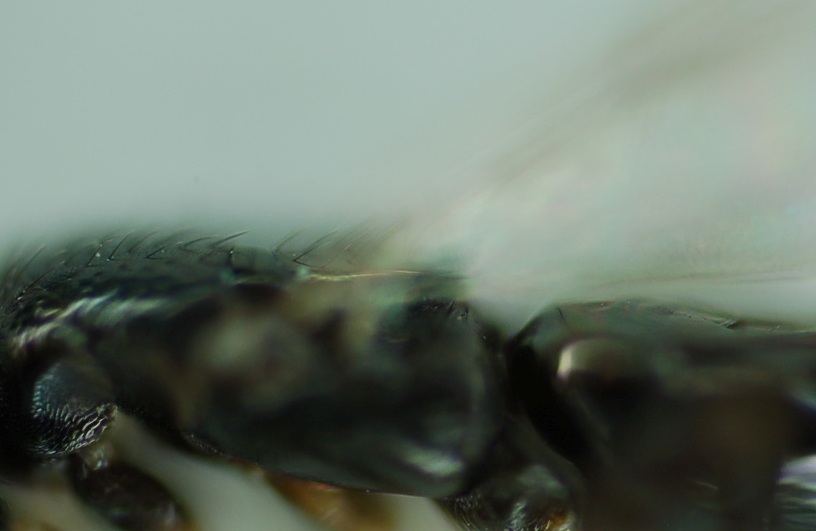

Supplement: S2 Dataset — (ZIP) [file pone.0191085.s002.zip › data set 2/Img24.jpg]

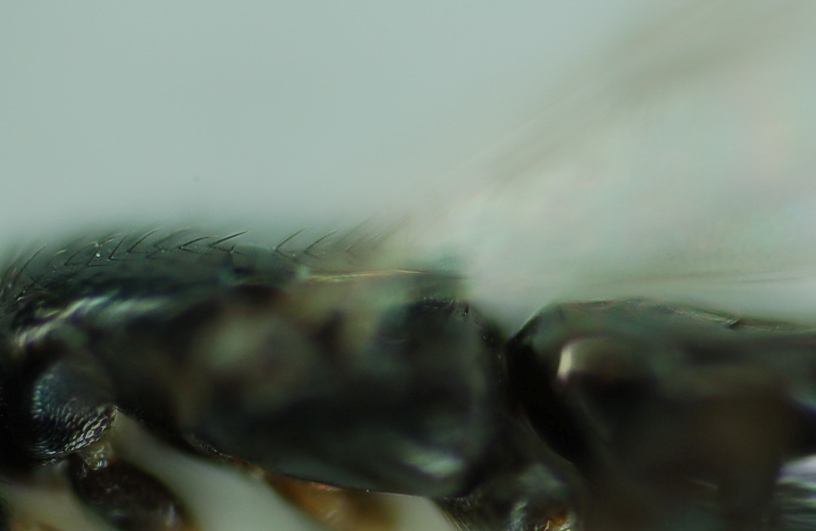

Supplement: S2 Dataset — (ZIP) [file pone.0191085.s002.zip › data set 2/Img25.jpg]

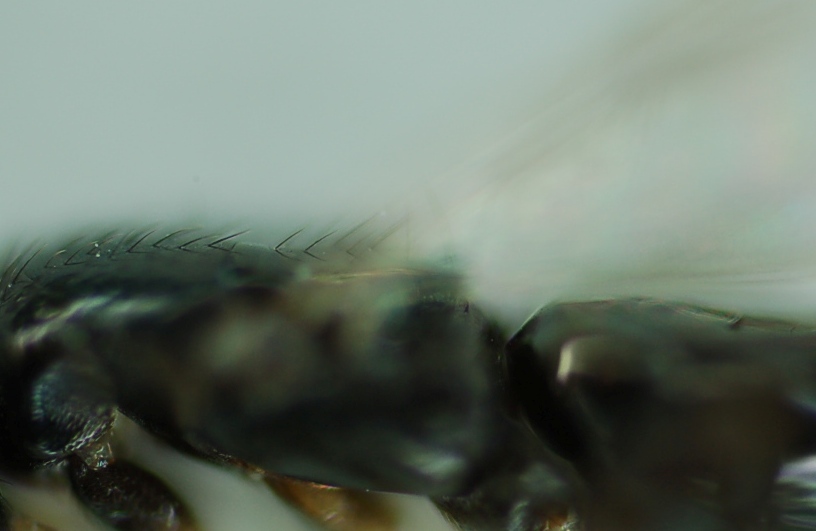

Supplement: S2 Dataset — (ZIP) [file pone.0191085.s002.zip › data set 2/Img26.jpg]

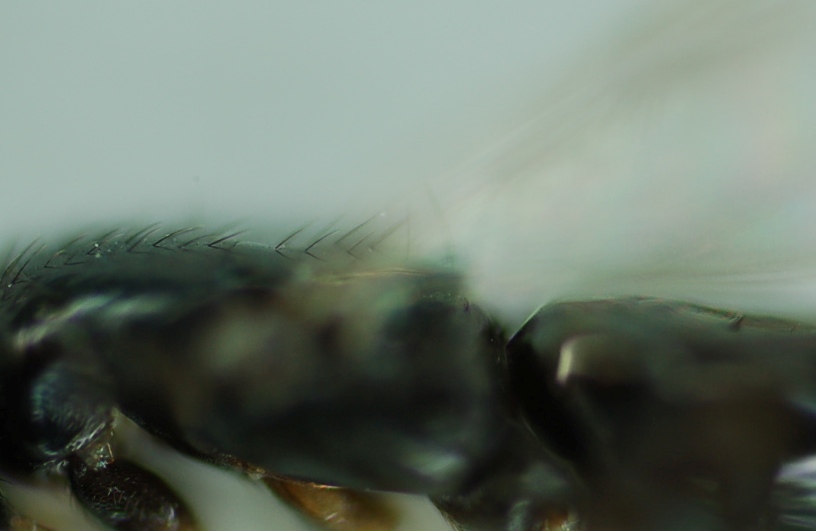

Supplement: S2 Dataset — (ZIP) [file pone.0191085.s002.zip › data set 2/Img27.jpg]

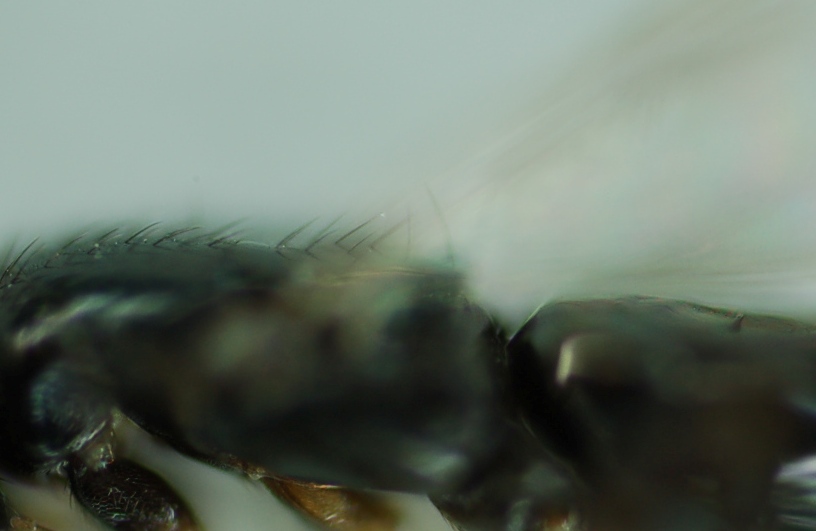

Supplement: S2 Dataset — (ZIP) [file pone.0191085.s002.zip › data set 2/Img28.jpg]

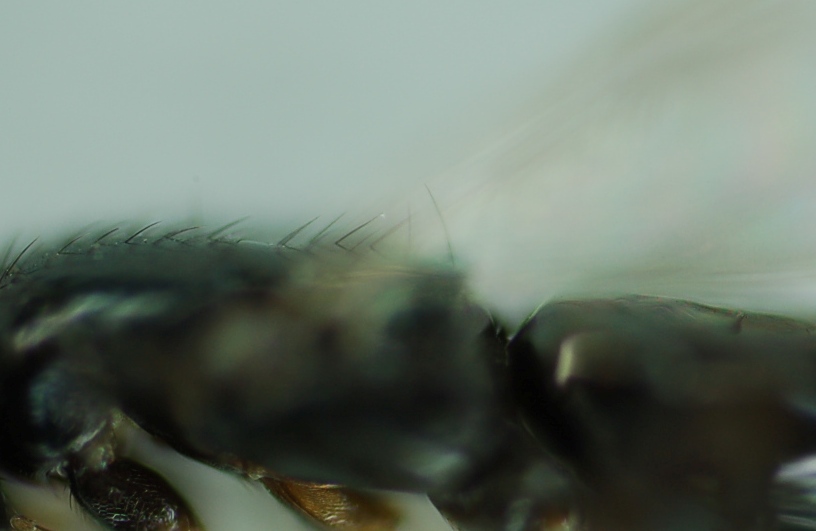

Supplement: S2 Dataset — (ZIP) [file pone.0191085.s002.zip › data set 2/Img29.jpg]

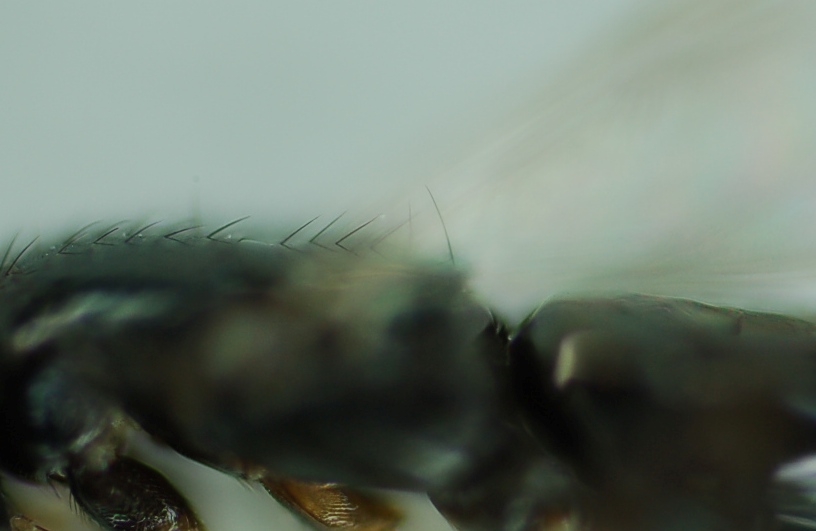

Supplement: S2 Dataset — (ZIP) [file pone.0191085.s002.zip › data set 2/Img30.jpg]

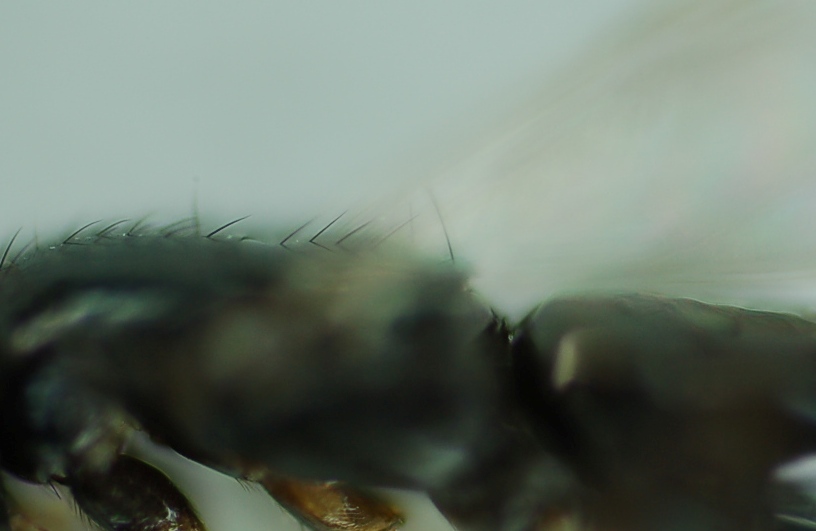

Supplement: S2 Dataset — (ZIP) [file pone.0191085.s002.zip › data set 2/Img31.jpg]

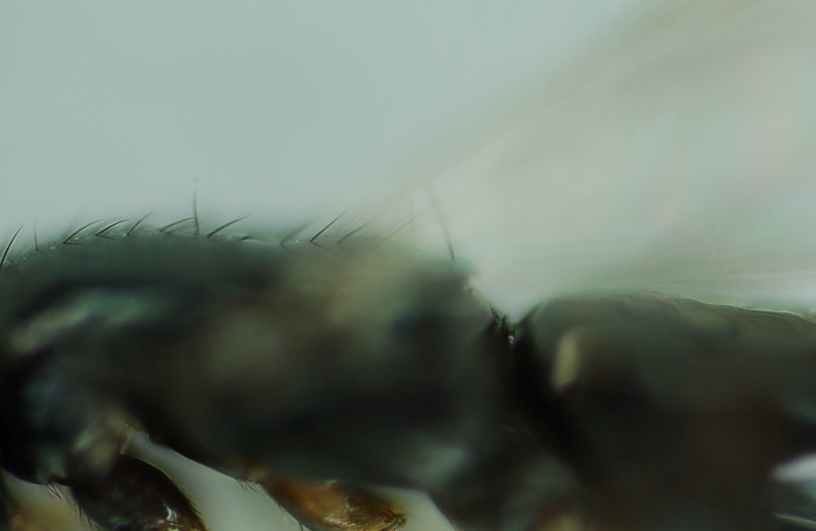

Supplement: S2 Dataset — (ZIP) [file pone.0191085.s002.zip › data set 2/Img32.jpg]

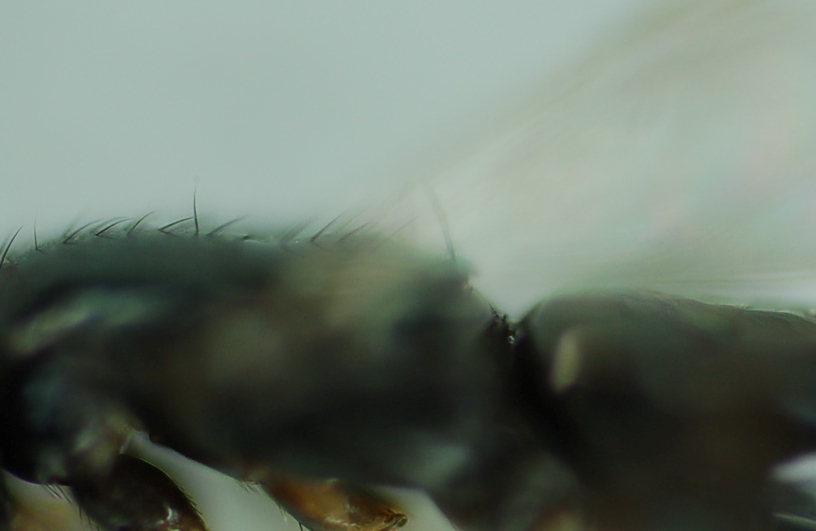

Supplement: S2 Dataset — (ZIP) [file pone.0191085.s002.zip › data set 2/Img33.jpg]

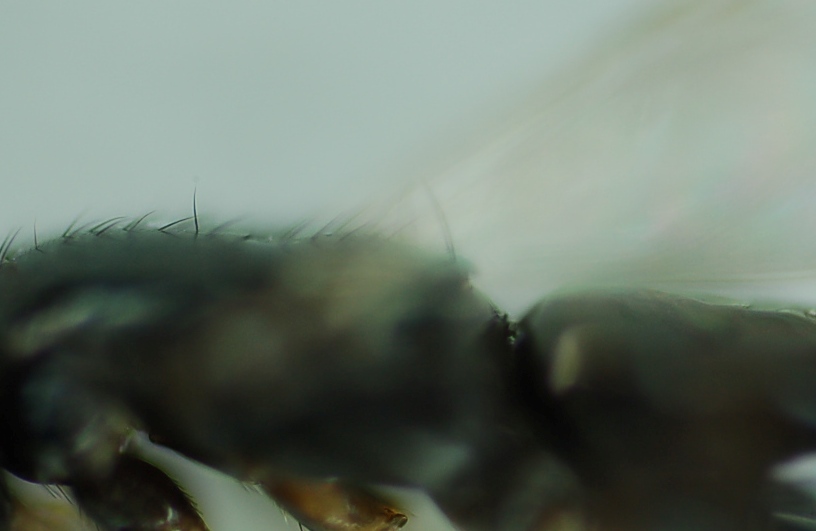

Supplement: S2 Dataset — (ZIP) [file pone.0191085.s002.zip › data set 2/Img34.jpg]

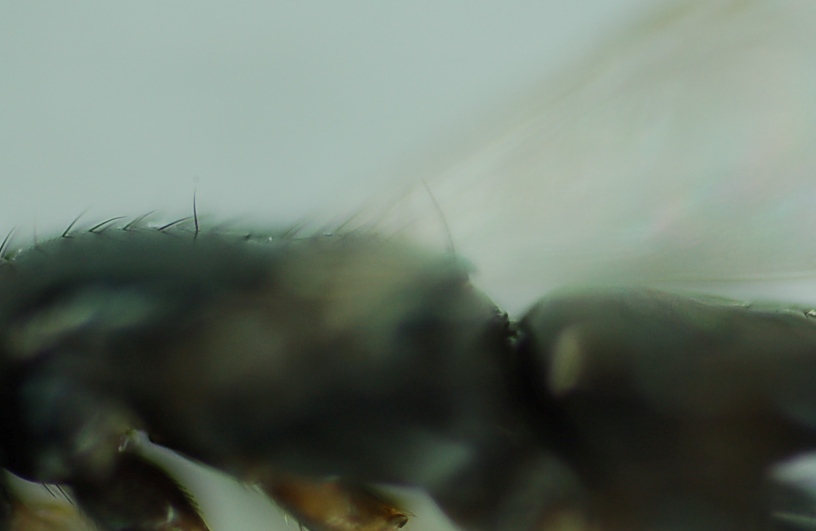

Supplement: S2 Dataset — (ZIP) [file pone.0191085.s002.zip › data set 2/Img35.jpg]

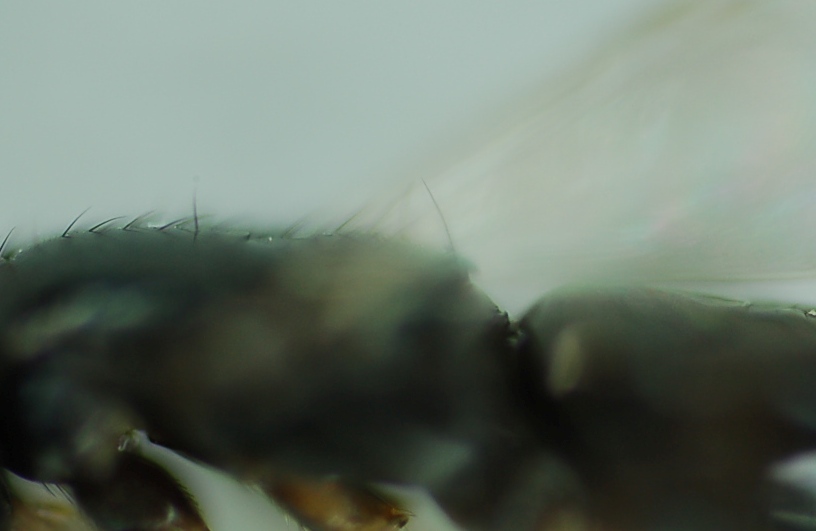

Supplement: S2 Dataset — (ZIP) [file pone.0191085.s002.zip › data set 2/Img36.jpg]

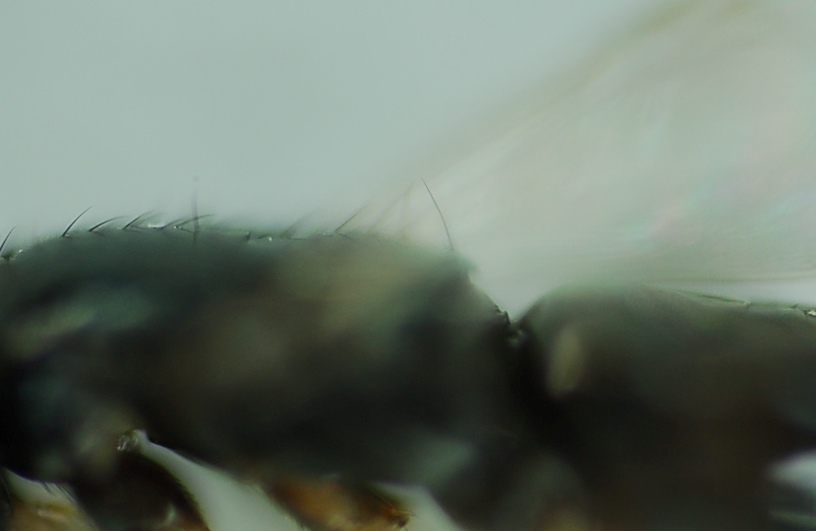

Supplement: S2 Dataset — (ZIP) [file pone.0191085.s002.zip › data set 2/Img37.jpg]

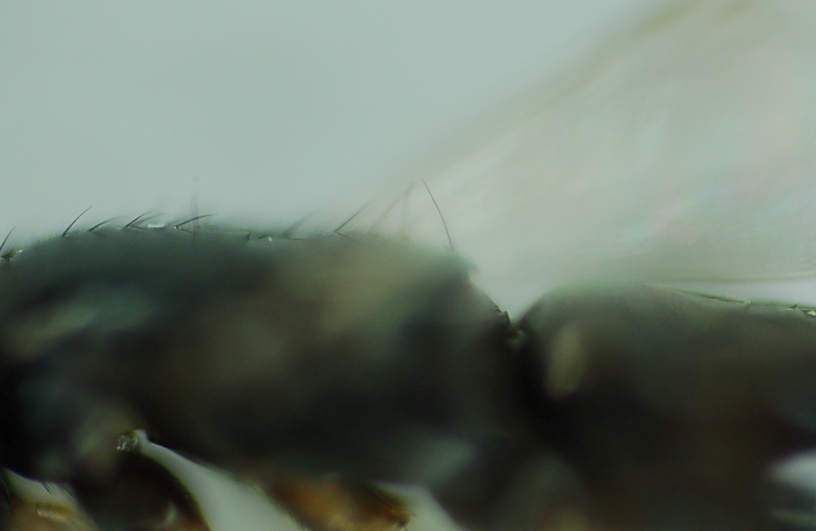

Supplement: S2 Dataset — (ZIP) [file pone.0191085.s002.zip › data set 2/Img38.jpg]

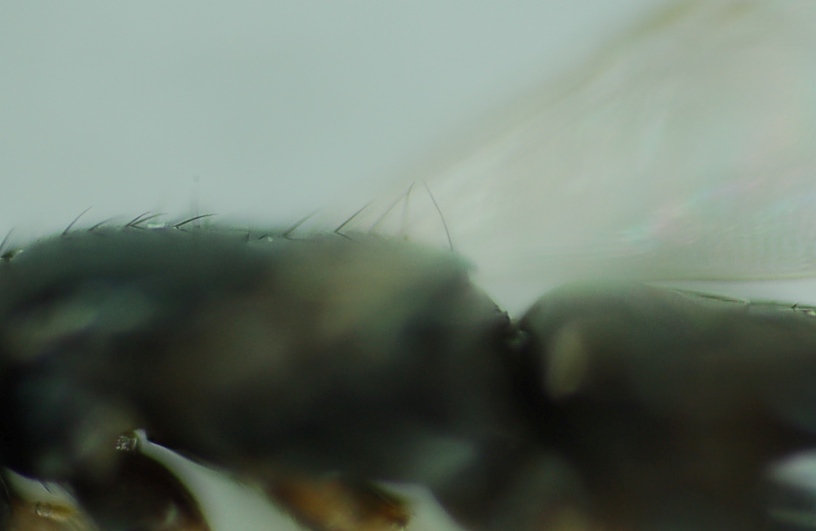

Supplement: S2 Dataset — (ZIP) [file pone.0191085.s002.zip › data set 2/Img39.jpg]

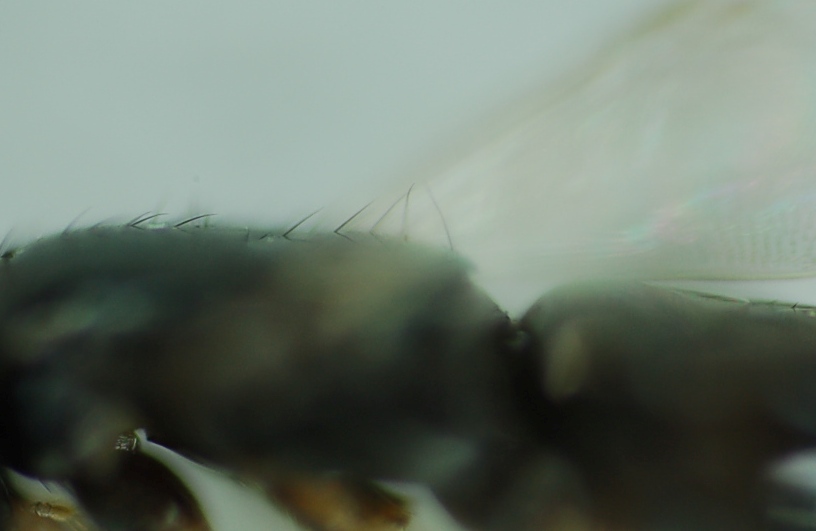

Supplement: S2 Dataset — (ZIP) [file pone.0191085.s002.zip › data set 2/Img40.jpg]

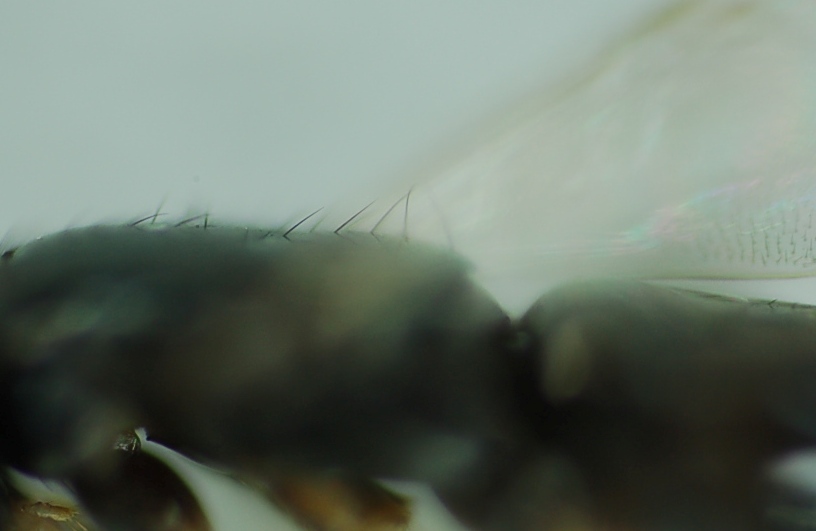

Supplement: S2 Dataset — (ZIP) [file pone.0191085.s002.zip › data set 2/Img41.jpg]

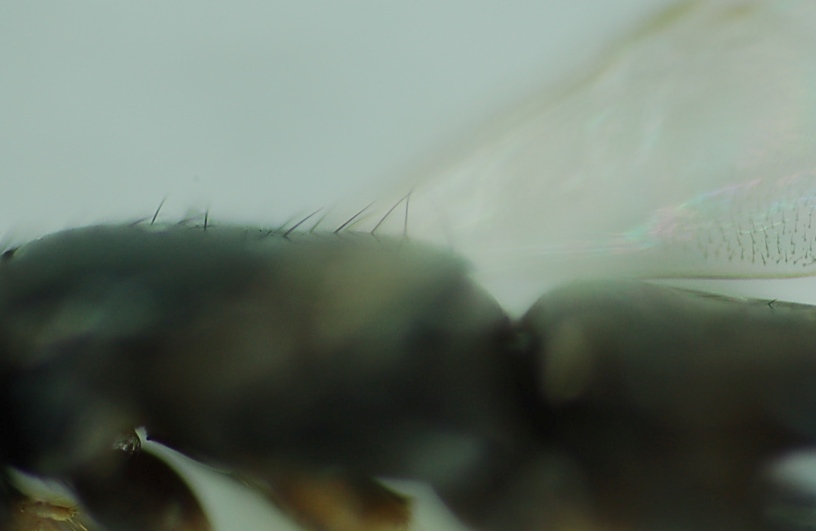

Supplement: S2 Dataset — (ZIP) [file pone.0191085.s002.zip › data set 2/Img42.jpg]

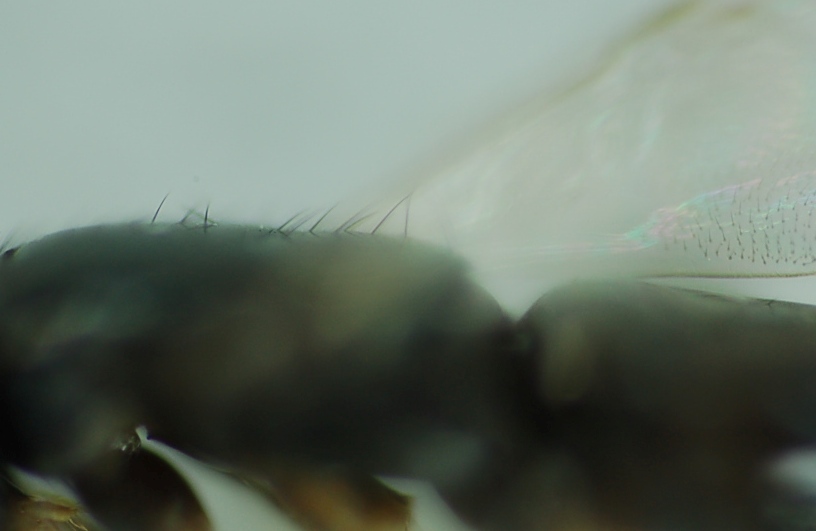

Supplement: S2 Dataset — (ZIP) [file pone.0191085.s002.zip › data set 2/Img43.jpg]

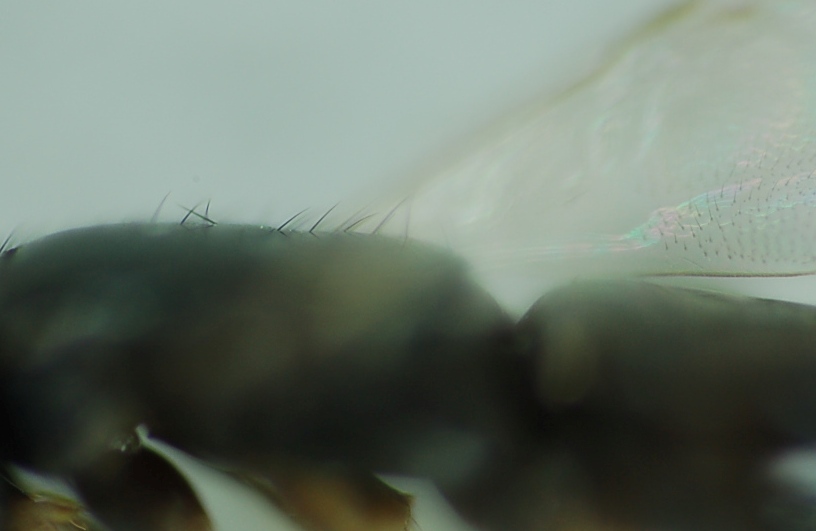

Supplement: S2 Dataset — (ZIP) [file pone.0191085.s002.zip › data set 2/Img44.jpg]

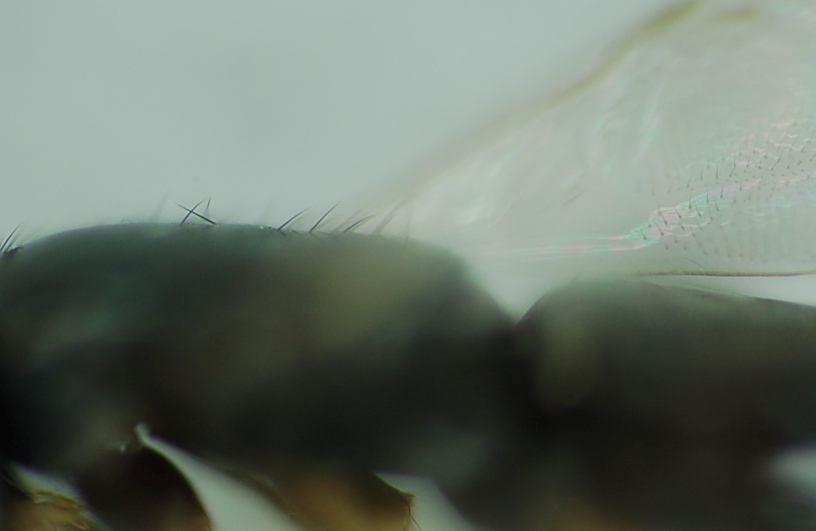

Supplement: S2 Dataset — (ZIP) [file pone.0191085.s002.zip › data set 2/Img45.jpg]

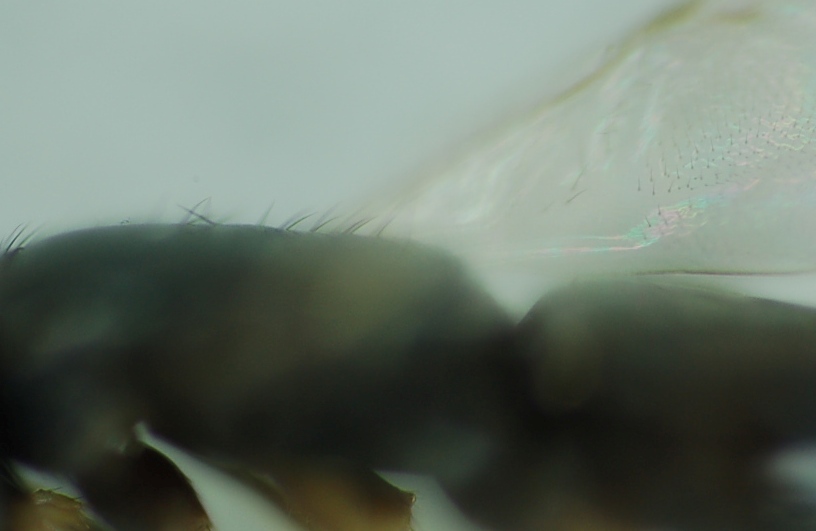

Supplement: S2 Dataset — (ZIP) [file pone.0191085.s002.zip › data set 2/Img46.jpg]

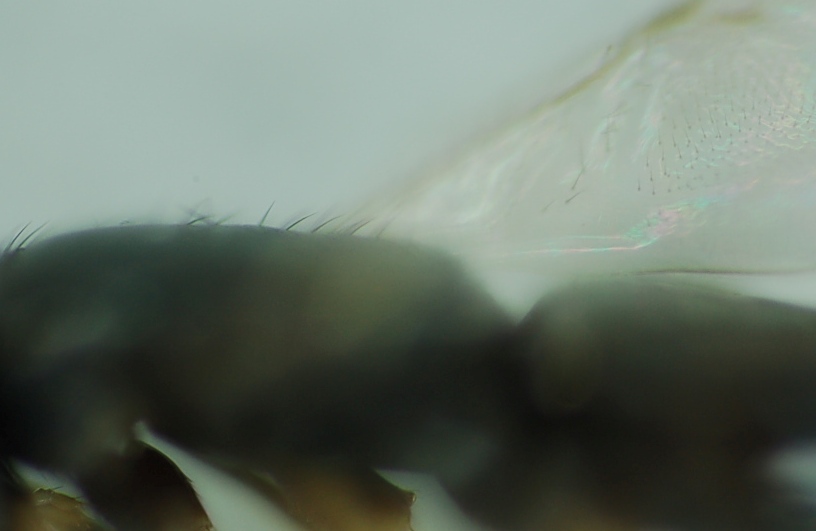

Supplement: S2 Dataset — (ZIP) [file pone.0191085.s002.zip › data set 2/Img47.jpg]

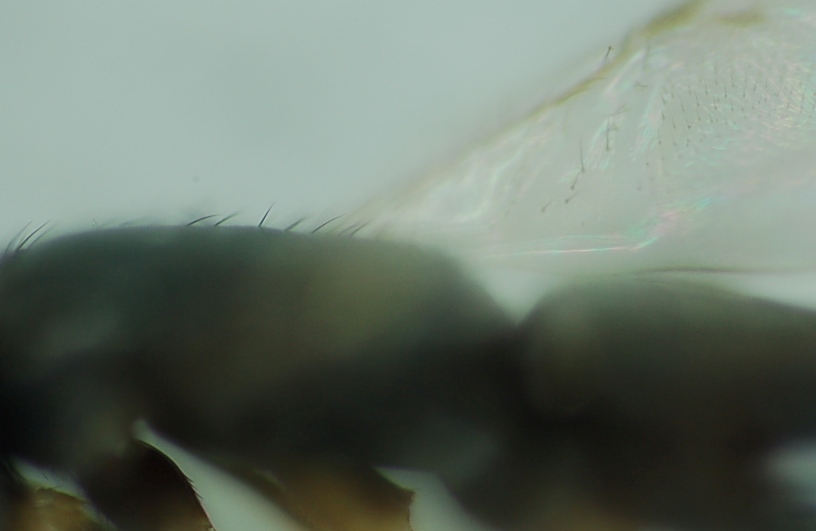

Supplement: S2 Dataset — (ZIP) [file pone.0191085.s002.zip › data set 2/Img48.jpg]

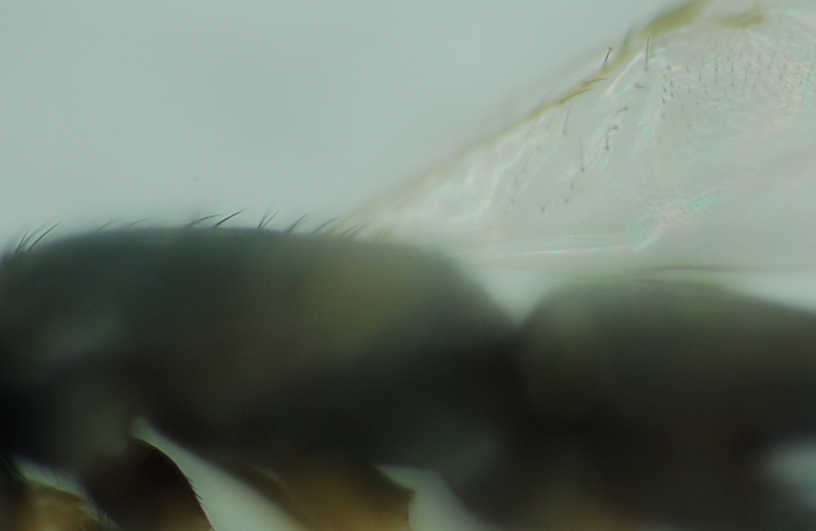

Supplement: S2 Dataset — (ZIP) [file pone.0191085.s002.zip › data set 2/Img49.jpg]

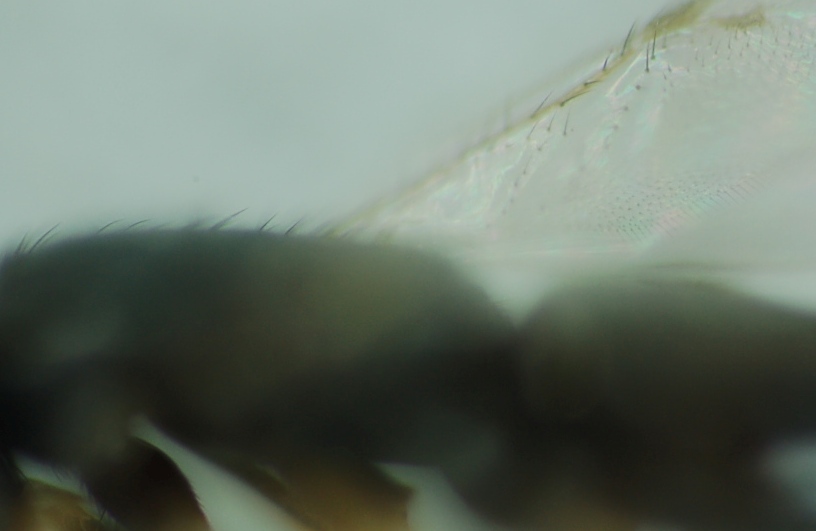

Supplement: S2 Dataset — (ZIP) [file pone.0191085.s002.zip › data set 2/Img50.jpg]

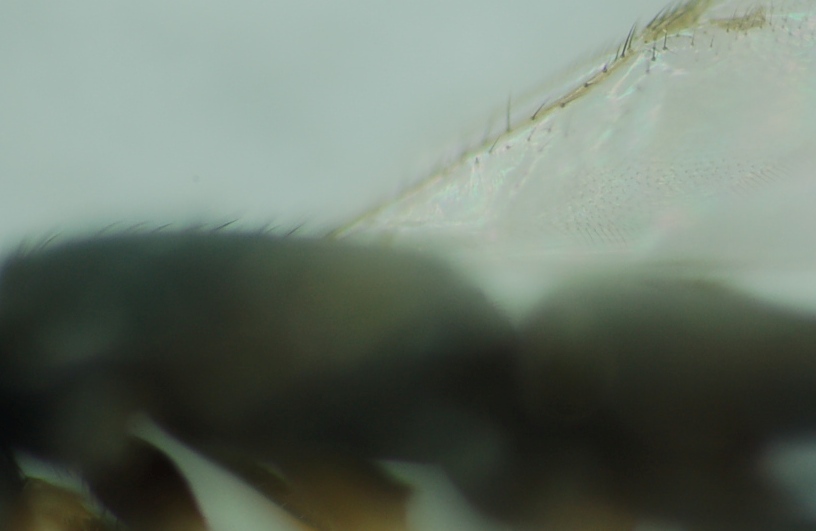

Supplement: S2 Dataset — (ZIP) [file pone.0191085.s002.zip › data set 2/Img51.jpg]

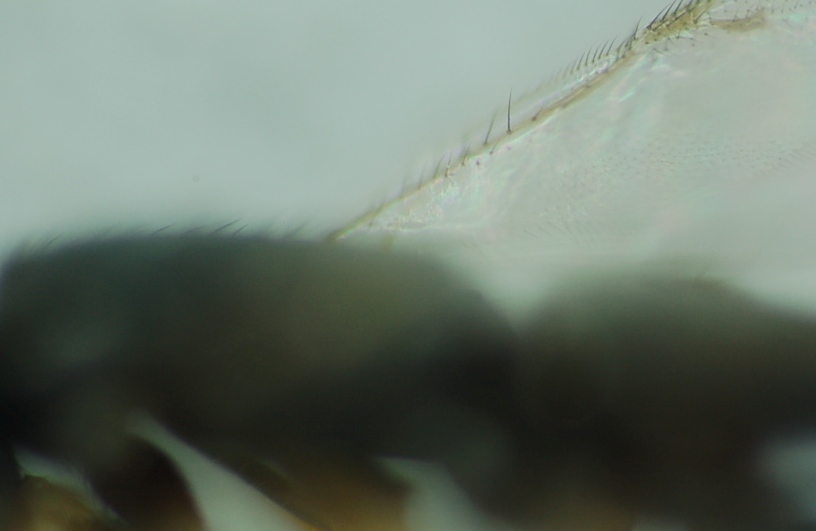

Supplement: S2 Dataset — (ZIP) [file pone.0191085.s002.zip › data set 2/Img52.jpg]

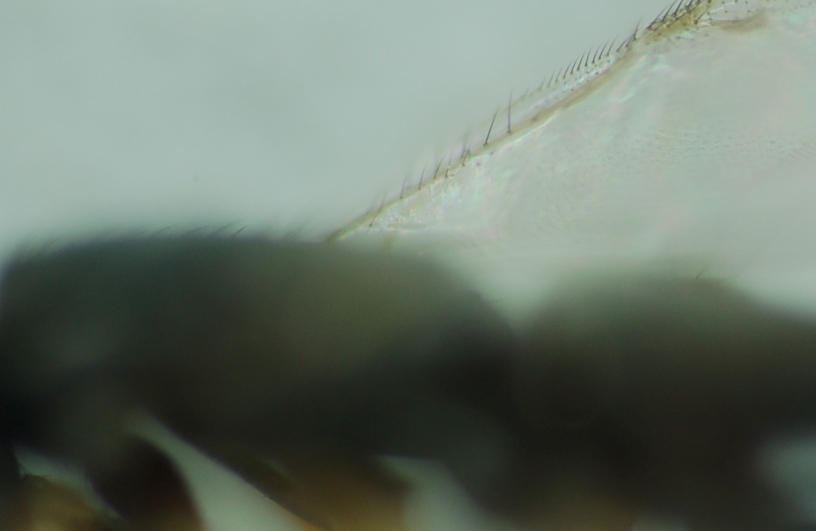

Supplement: S2 Dataset — (ZIP) [file pone.0191085.s002.zip › data set 2/Img53.jpg]

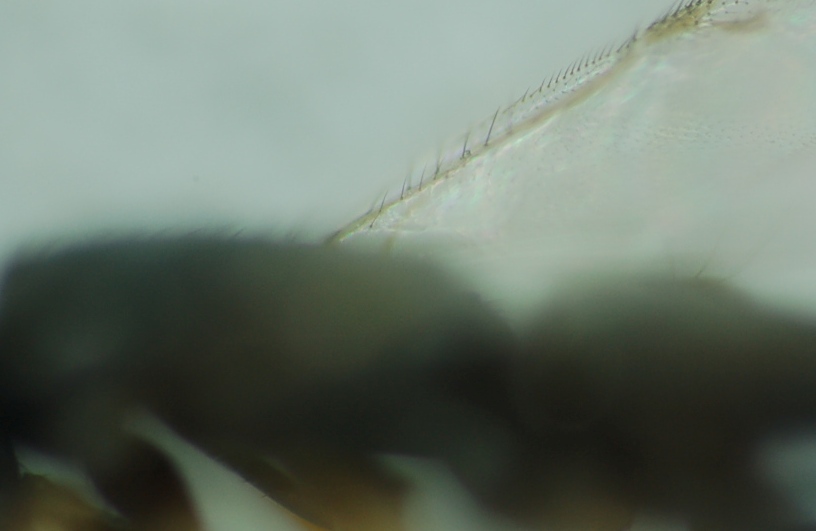

Supplement: S2 Dataset — (ZIP) [file pone.0191085.s002.zip › data set 2/Img54.jpg]

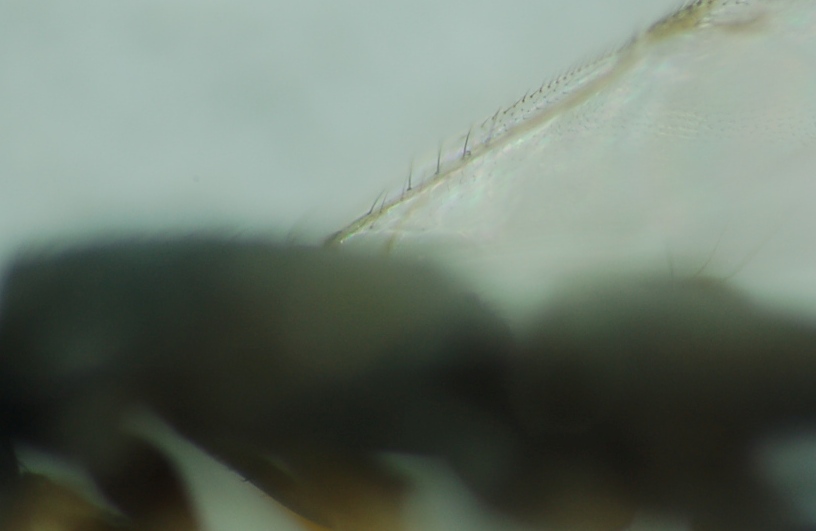

Supplement: S2 Dataset — (ZIP) [file pone.0191085.s002.zip › data set 2/Img55.jpg]

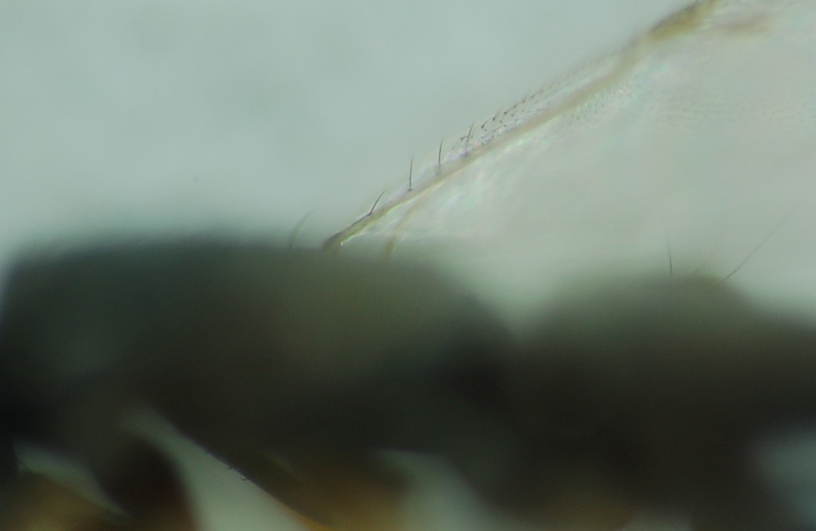

Supplement: S2 Dataset — (ZIP) [file pone.0191085.s002.zip › data set 2/Img56.jpg]

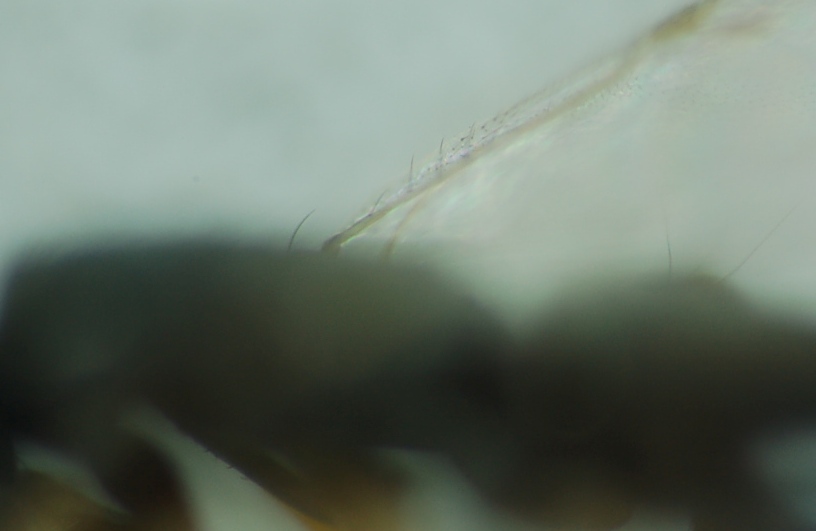

Supplement: S2 Dataset — (ZIP) [file pone.0191085.s002.zip › data set 2/Img57.jpg]

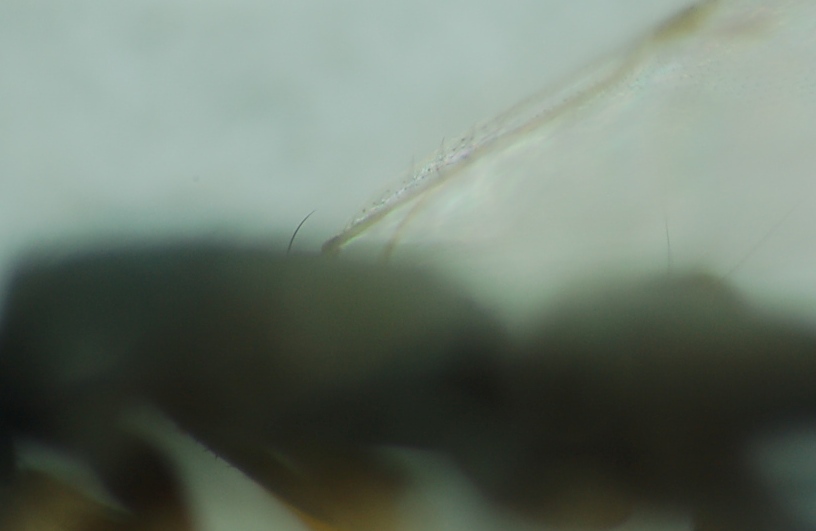

Supplement: S2 Dataset — (ZIP) [file pone.0191085.s002.zip › data set 2/Img58.jpg]

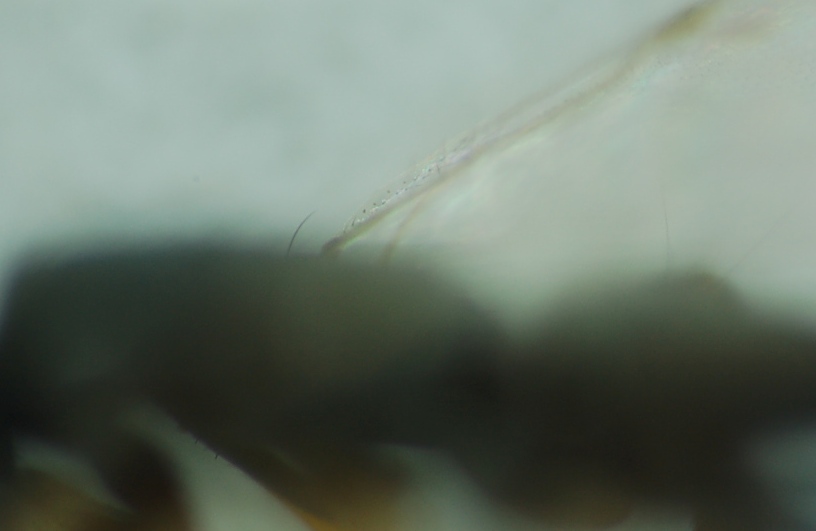

Supplement: S2 Dataset — (ZIP) [file pone.0191085.s002.zip › data set 2/Img59.jpg]

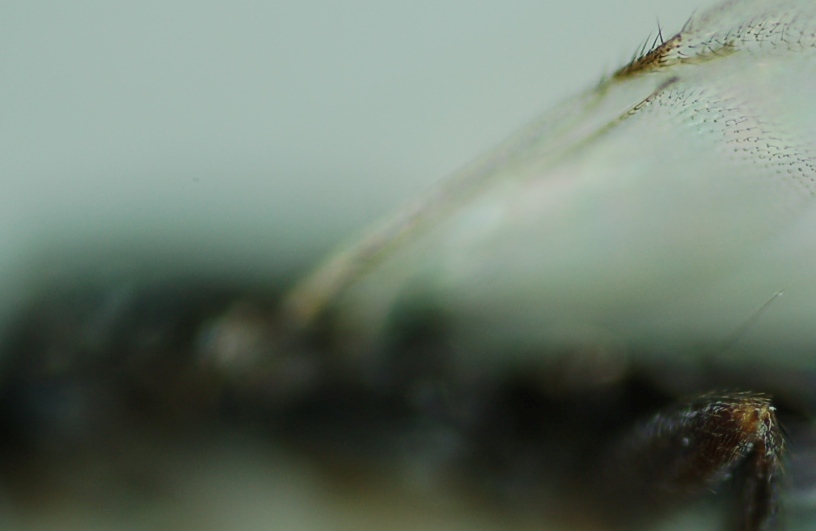

Supplement: S2 Dataset — (ZIP) [file pone.0191085.s002.zip › data set 2/Img6.jpg]

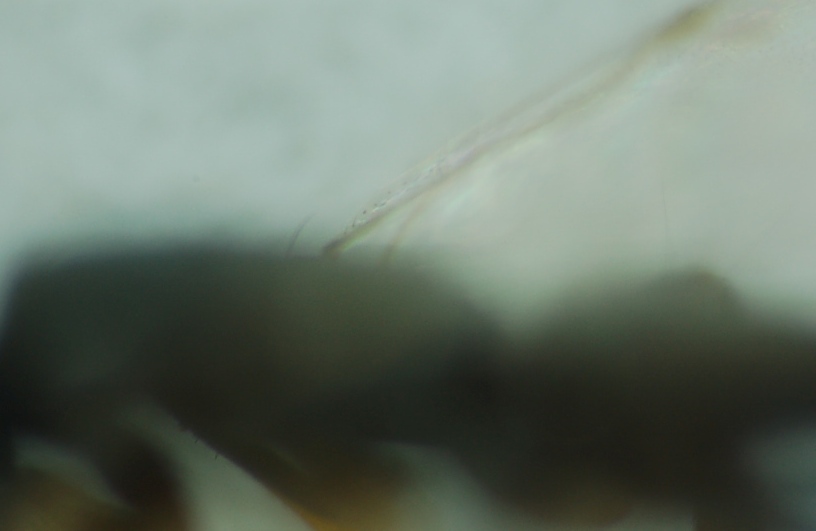

Supplement: S2 Dataset — (ZIP) [file pone.0191085.s002.zip › data set 2/Img60.jpg]

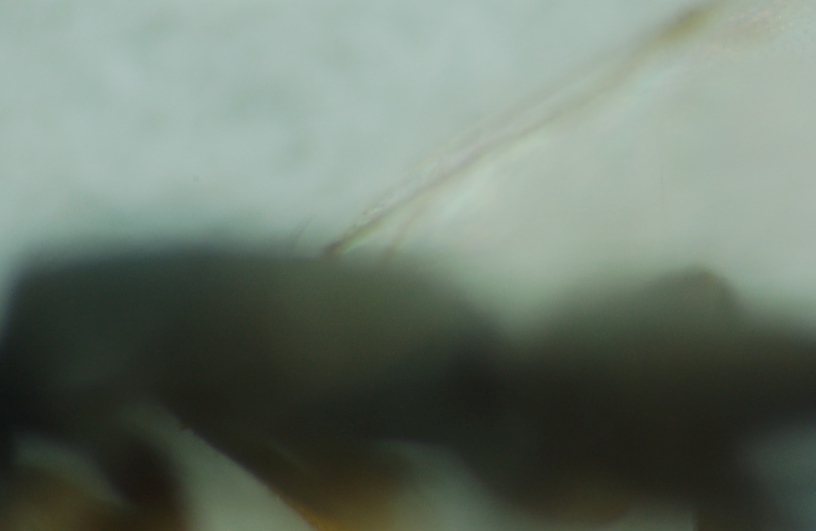

Supplement: S2 Dataset — (ZIP) [file pone.0191085.s002.zip › data set 2/Img61.jpg]

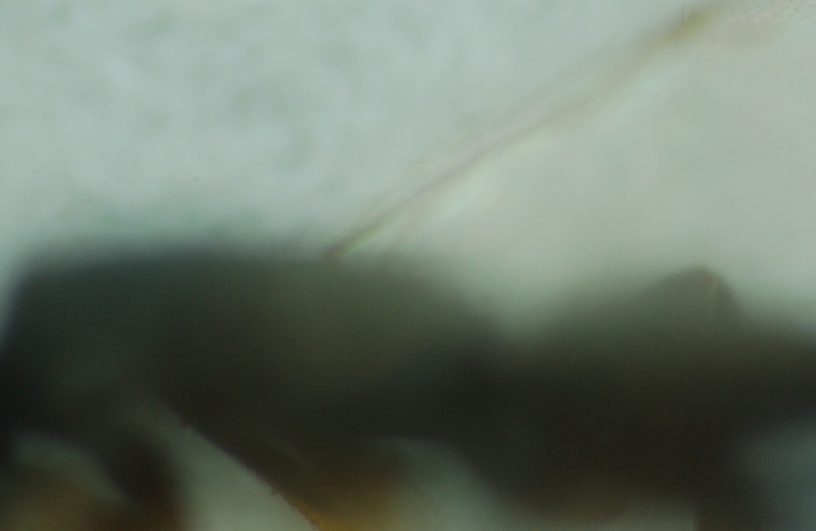

Supplement: S2 Dataset — (ZIP) [file pone.0191085.s002.zip › data set 2/Img62.jpg]

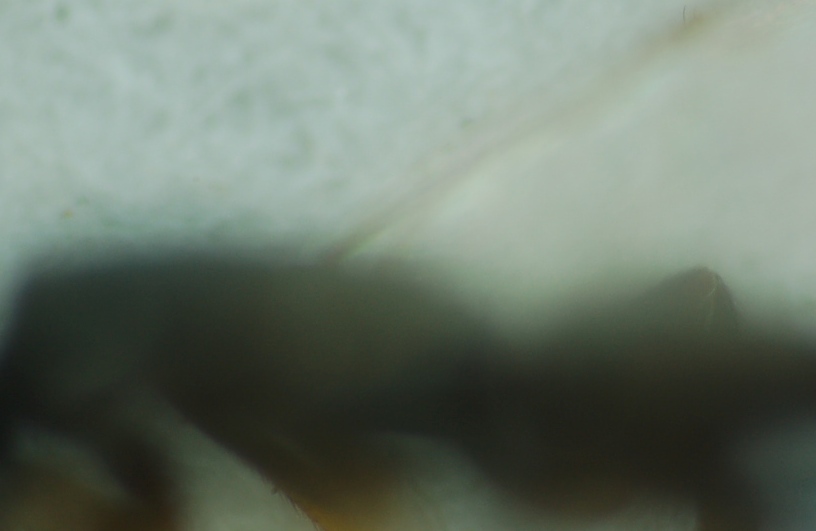

Supplement: S2 Dataset — (ZIP) [file pone.0191085.s002.zip › data set 2/Img63.jpg]

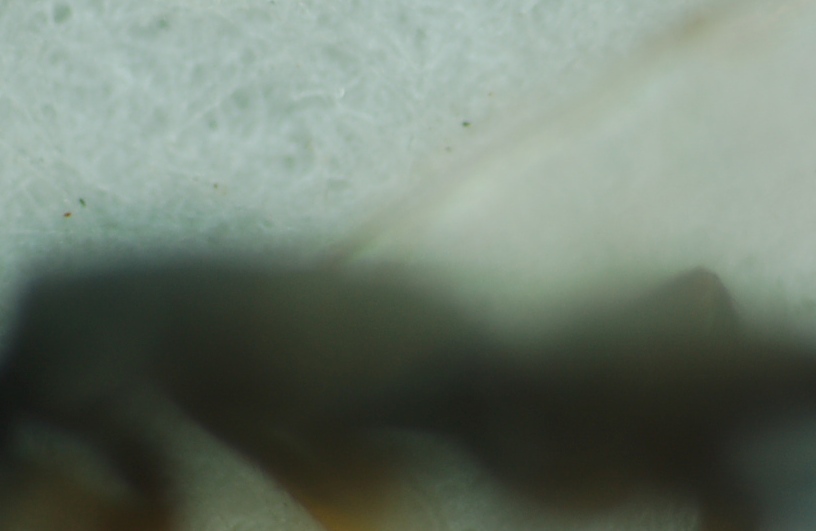

Supplement: S2 Dataset — (ZIP) [file pone.0191085.s002.zip › data set 2/Img64.jpg]

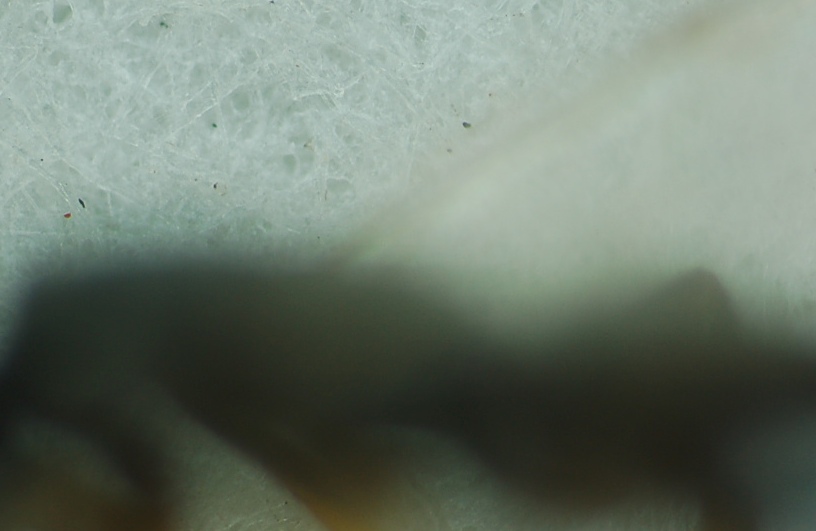

Supplement: S2 Dataset — (ZIP) [file pone.0191085.s002.zip › data set 2/Img65.jpg]
