# Supplementary figures and images for: A multi-focus image fusion method via region mosaicking on Laplacian pyramids (part 2 of 2)
Source: PLoS One. 2018 May 17;13(5):e0191085. doi: 10.1371/journal.pone.0191085 (PMC5957432; doi:10.1371/journal.pone.0191085)

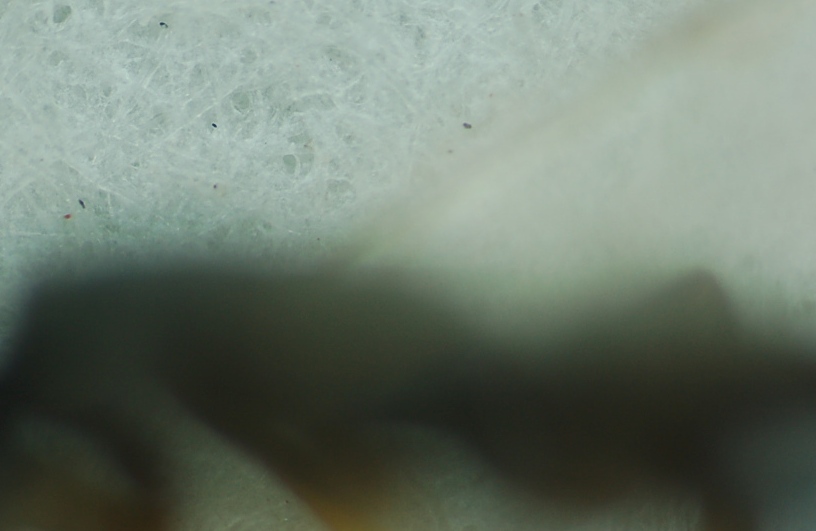

Supplement: S2 Dataset — (ZIP) [file pone.0191085.s002.zip › data set 2/Img66.jpg]

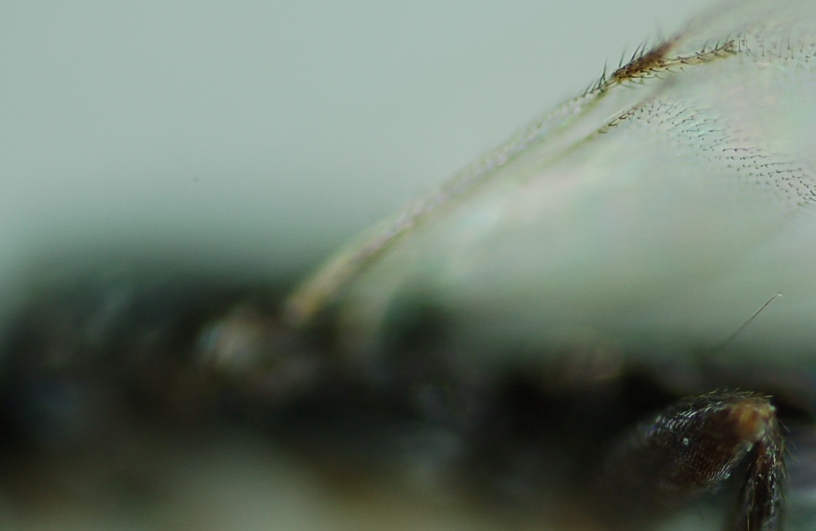

Supplement: S2 Dataset — (ZIP) [file pone.0191085.s002.zip › data set 2/Img7.jpg]

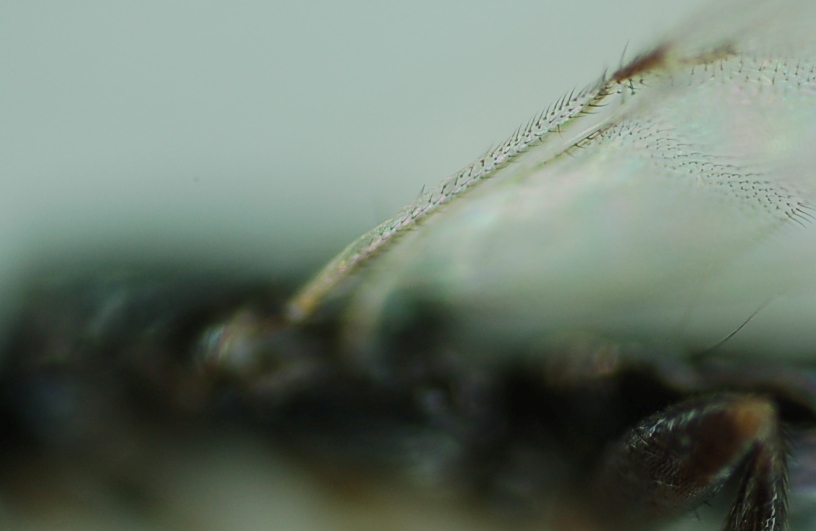

Supplement: S2 Dataset — (ZIP) [file pone.0191085.s002.zip › data set 2/Img8.jpg]

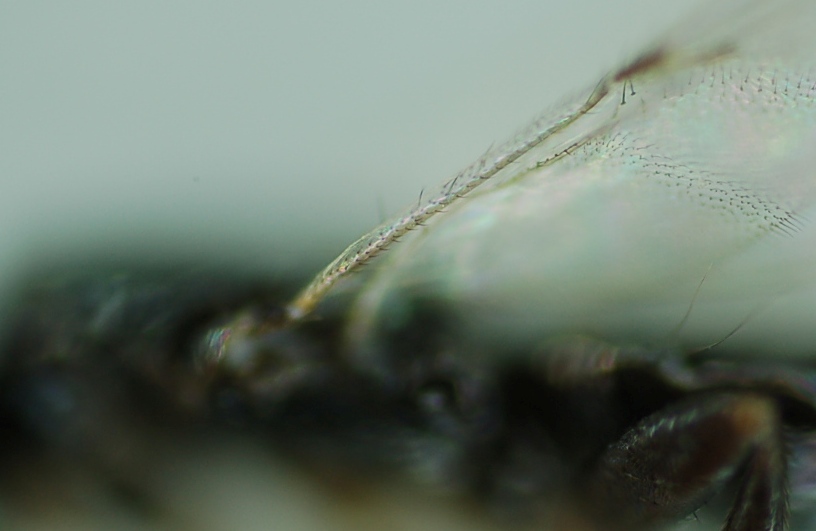

Supplement: S2 Dataset — (ZIP) [file pone.0191085.s002.zip › data set 2/Img9.jpg]

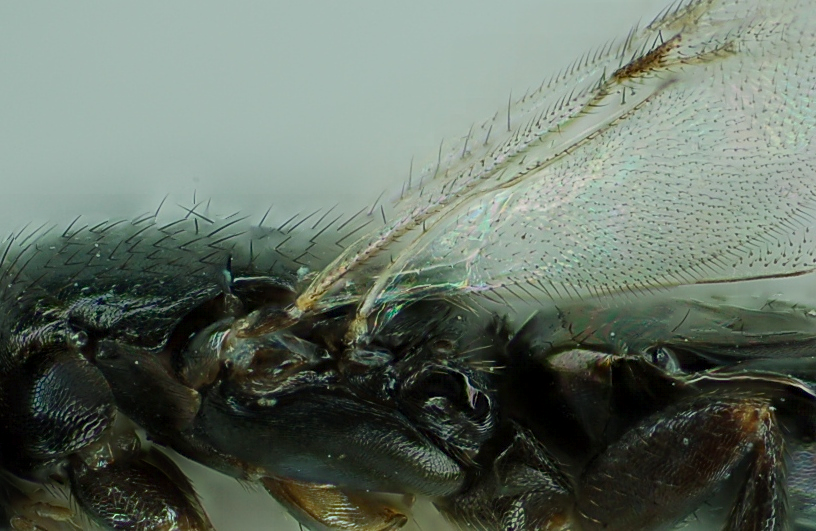

Supplement: S2 Dataset — (ZIP) [file pone.0191085.s002.zip › data set 2/img6.jpg_result.bmp]
